# Primary care risk stratification in COPD using routinely collected data: a secondary data analysis

**DOI:** 10.1038/s41533-019-0154-6

**Published:** 2019-12-04

**Authors:** Matthew Johnson, Lucy Rigge, David Culliford, Lynn Josephs, Mike Thomas, Tom Wilkinson

**Affiliations:** 1 MRC Lifecourse Epidemiology Unit, University of Southampton, Southampton, UK; 2 NIHR ARC Wessex Data Science Hub, Faculty of Health Sciences, University of Southampton, Southampton, UK; 3 NIHR ARC Wessex, University of Southampton, Southampton, UK; 4 NIHR Respiratory Biomedical Research Unit, Southampton General Hospital, Southampton, UK; 5 Department of Primary Care & Population Sciences, Faculty of Medicine, University of Southampton, Southampton, UK; 6 Clinical and Experimental Sciences, University of Southampton Faculty of Medicine, Southampton General Hospital, Southampton, UK; 7 Wessex Investigational Sciences Hub, University of Southampton Faculty of Medicine, Southampton General Hospital, Southampton, UK

**Keywords:** Chronic obstructive pulmonary disease, Epidemiology

## Abstract

Most clinical contacts with chronic obstructive pulmonary disease (COPD) patients take place in primary care, presenting opportunity for proactive clinical management. Electronic health records could be used to risk stratify diagnosed patients in this setting, but may be limited by poor data quality or completeness. We developed a risk stratification database algorithm using the DOSE index (Dyspnoea, Obstruction, Smoking and Exacerbation) with routinely collected primary care data, aiming to calculate up to three repeated risk scores per patient over five years, each separated by at least one year. Among 10,393 patients with diagnosed COPD, sufficient primary care data were present to calculate at least one risk score for 77.4%, and the maximum of three risk scores for 50.6%. Linked secondary care data revealed primary care under-recording of hospital exacerbations, which translated to a slight, non-significant cohort average risk score reduction, and an understated risk group allocation for less than 1% of patients. Algorithmic calculation of the DOSE index is possible using primary care data, and appears robust to the absence of linked secondary care data, if unavailable. The DOSE index appears a simple and practical means of incorporating risk stratification into the routine primary care of COPD patients, but further research is needed to evaluate its clinical utility in this setting. Although secondary analysis of routinely collected primary care data could benefit clinicians, patients and the health system, standardised data collection and improved data quality and completeness are also needed.

## Introduction

The diagnosed prevalence of chronic obstructive pulmonary disease (COPD) was recently placed at around one million people in England,^1^ although there may be an estimated two million more without formal diagnosis.^2^ It is the second most common reason for emergency National Health Service (NHS) hospital admission, representing a significant cost.^2^ Recognising the growing burden of long-term conditions on the health system, current NHS policy emphasises a need to move away from the traditional model of care, arguing instead for greater focus on the delivery of preventative care out of hospital, and concomitant shift in investment from secondary care to primary care and community services.^3–5^ Although a sudden and rapid worsening of symptoms (known as COPD exacerbation) may sometimes necessitate hospital attendance, most clinical contacts with diagnosed patients take place in the primary care setting,^6^ presenting opportunities to proactively manage the condition and reduce the risk of hospitalisation. Limited resources in primary care, however, dictate a need for methods to identify and target those patients at greatest risk. As a heterogeneous, progressive condition with multiple phenotypes,^7^ arguments for a range of predictors being more powerful than any single clinical marker alone have motivated the development of several multicomponent indices for prognosis and risk stratification in COPD.^8,9^

The DOSE index comprises Dyspnoea, airflow Obstruction, Smoking status and COPD Exacerbation components (Table 1),^10^ and has been shown to be associated with respiratory failure and healthcare utilisation,^10^ future exacerbation,^11^ mortality,^12,13^ physical activity and BMI,^14^ and worsening health status as measured by self-reported Clinical COPD Questionnaire.^15^ Several practical advantages further commend its use in primary care in preference to other available indices. First, its operational simplicity permits assessment within the time and space constraints of a primary care consultation, in which the 6-min walk test required for the BODE index (Body mass index, airflow Obstruction, Dyspnoea and Exercise capacity)^8^ would be impractical.^10,16^ In addition, the inclusion of Smoking and Exacerbation means that the DOSE index consists of components that are directly modifiable by clinical intervention, are *already* routinely assessed in primary care, and could often be retrievable from existing data without additional clinical investigation; further advantages over the ADO (Age, Dyspnoea and airflow Obstruction)^17^ and BODE indices. Regular clinical review of the DOSE score in this setting could therefore facilitate a proactive and personalised approach to patient care, prompting reflection on how best to address any deterioration in its constituent components when creating a management plan.^10,18^

**Table 1:** Scores allocated to individual DOSE Index components.

		Score allocation			
		0 points	1 point	2 points	3 points
Component	MRC dyspnoea scale (Dyspnoea)	0–1	2	3	4
	FEV_1_% predicted (Obstruction)	≥50%	30%–50%	≤30%	n/a
	Smoking status (Smoking)	Non-smoker	Smoker	n/a	n/a
	Number of exacerbations (Exacerbation)	0–1	2–3	≥3	n/a

Recent years have seen promising developments around the use of database algorithms to identify at-risk patients using routinely collected electronic health record (EHR) data.^19^ An important consideration, however, is that, to date, EHRs’ principal role has been to support the provision of patient care, which may give rise to systematic data omissions and inaccuracies, thereby limiting secondary applications beyond this purpose.^20^ Of particular relevance, segments of the patient pathway may be absent from the EHR if the patient underwent a period of treatment elsewhere.^21^ As NHS primary care practices rarely have direct access to live secondary care data, they are often reliant on retrospective recording of hospital attendances from discharge summaries.^19^ Recent studies examining exacerbation^22^ and community-acquired pneumonia,^23^ which may involve primary care or hospital presentation, observed a reduced incidence rate using stand-alone primary care (SaPC) data compared to linked primary care/secondary care (PC/SC) data, suggesting incomplete recording of hospital events in the primary care EHR. This may preclude algorithmic use of the DOSE index to identify at-risk patients in primary care, as any systematic omission of hospital exacerbations could limit its accuracy and predictive power.

Our overall aim was to develop a database algorithm based on the DOSE index for primary care risk stratification of patients with COPD. In addition, under the hypothesis that linkage to secondary care data would reveal exacerbations that were absent from the primary care EHR, we aimed to describe and compare their incidence using SaPC data and PC/SC data, and the extent to which algorithmically calculated DOSE scores varied by data type.

## Results

We identified 10,393 patients with COPD diagnosis (52.5% male; mean age 67.7, SD ± 10.5 years); most (55.9%) had sufficient Obstruction records present to qualify for entry into the algorithm at three separate instances during the 5-year study period, each separated by a minimum of 1 year (Table 2). Having qualified for entry, the presence of attendant Dyspnoea and Smoking records was consistently high, at over 90% across all instances. As a result, 8047 patients were found to have at least one complete DOSE score available during the study period; 87.4% of those qualifying for entry to the algorithm at the first instance or 77.4% of the cohort as a whole. Although only 7228 patients were found to have at least two complete scores and 5264 to have three, this represented an increase to 89.6% and 90.7% of those qualifying at the second and third instances, respectively. The proportion of patients with a non-zero Exacerbation score was found to differ by data type; PC/SC data generated an excess across all instances, to a maximum of an additional 157 patients gaining a non-zero score at the second instance.

**Table 2:** Availability of complete DOSE scores and individual components by instance.

	Available at instance		
	1^st^	2^nd^	3^rd^
Obstruction; *n* (%)^a^	9212 (88.6)	8063 (77.6)	5807 (55.9)
Dyspnoea; *n* (%)^b^	8445 (91.7)	7633 (94.7)	5556 (95.7)
Smoking; *n* (%)^b^	8674 (94.2)	7560 (93.8)	5460 (94.0)
Complete DOSE score; *n* (%)^b^	8047 (87.4)	7228 (89.6)	5264 (90.7)
Non-zero Exacerbation score; SaPC data, *n* (%)^b^	1644 (17.9)	1681 (20.9)	1252 (21.6)
Non-zero Exacerbation score; PC/SC data, *n* (%)^b^	1793 (19.5)	1838 (22.8)	1374 (23.7)

*SaPC* stand-alone primary care data, *PC/SC* linked primary care/secondary care data

^a^Of cohort, *N* = 10,393

^b^Of patients qualifying for entry into the DOSE algorithm at instance

Across all instances, complete DOSE scores were available for a greater proportion of patients in the two most deprived quintiles, but for a smaller proportion of those in the youngest and oldest age groups, or with one or no diagnosed comorbidities (Table 3). Assessment of baseline COPD severity was not possible for 26.6% of the cohort owing to absent FEV_1_% value within 1 year of the study start date (1 January 2010), a group of patients found to have markedly reduced DOSE score availability at all instances. Moderate airflow obstruction was the most common classification, but those with mild obstruction had the fewest DOSE scores available at all instances.

**Table 3:** Demographic characteristics of cohort at baseline, and complete DOSE score availability by instance.

	Complete DOSE score available at instance			
	*n* (%)^a^	1^st^ (%)^b^	2^nd^ (%)^b^	3^rd^ (%)^b^
Sex				
Male	5453 (52.5)	78.4	71.9	52.4
Female	4940 (47.5)	76.4	66.9	48.7
Age band				
59 years or younger	2134 (20.5)	74.9	64.3	43.1
60–69 years	3580 (34.5)	78.8	72.5	53.0
70–79 years	3338 (32.1)	79.2	72.5	55.4
80 years or older	1341 (12.9)	73.5	62.9	44.7
Deprivation quintile				
1 (most deprived)	1255 (12.1)	81.5	73.3	53.9
2	1963 (18.9)	78.4	70.9	54.4
3	1861 (17.9)	76.1	67.8	48.0
4	2153 (20.7)	76.0	68.3	49.3
5 (least deprived)	3155 (30.4)	77.1	69.3	49.6
Number of diagnosed comorbidities^c^				
None	1114 (10.7)	73.9	67.3	48.0
1	2447 (23.5)	76.3	67.9	49.4
2	2482 (23.9)	79.4	70.8	52.1
3	2002 (19.3)	77.4	71.6	50.5
4 or more	2348 (22.6)	78.3	69.3	51.8
Severity of airflow limitation in COPD (FEV_1_% predicted)				
Mild (≥80%)	1254 (12.1)	83.8	77.8	58.2
Moderate (≥50%–<80%)	4222 (40.6)	88.4	83.5	66.6
Severe (≥30%–<50%)	1823 (17.5)	89.3	88.3	64.5
Very severe (<30%)	331 (3.2)	86.7	79.8	60.4
Unknown	2763 (26.6)	48.8	34.1	12.5

^a^Of cohort, *N* = 10,393

^b^Within category

^c^Comorbidities considered were asthma, ischaemic heart disease, heart failure, cor pulmonale, hypertension, hyperlipidaemia, osteoporosis, cerebrovascular disease, dementia, gastro oesophageal reflux disease, peripheral vascular disease, connective tissue disease, anxiety/depression, lung cancer, chronic kidney disease, obstructive sleep apnoea, rhinosinusitis, pulmonary fibrosis, bronchiectasis and diabetes

Taking the study period as a whole, 55,410 exacerbations experienced by 8386 patients were identified using SaPC data, compared to 61,279 exacerbations by 8778 patients using PC/SC data; an excess of 5869 exacerbations, and 392 patients presenting to hospital only, with all events absent from the primary care EHR. This translated to a small proportion of individual patients with increased scores, up to 2.5% of patients with a score available at the second or third instances (Table 4). However, the Mann–Whitney test indicated a non-significant difference by data type at each instance (first, *z* = −0.749 *p* = 0.454; second, *z* = −0.955 *p* = 0.340; third, *z* = −0.741 *p* = 0.459). Most patients were classified to the lowest risk group but, again, using PC/SC data resulted in a small proportion of individual patients transitioning to a higher risk group, ranging from 0.5% of patients with a score available at the second instance, to 0.8% at the third instance.

**Table 4:** Comparison of stand-alone primary care data and linked primary/secondary care data DOSE scores/risk groups by instance.

	Instance					
	1^st^		2^nd^		3^rd^	
	SaPC	PC/SC	SaPC	PC/SC	SaPC	PC/SC
Patients with complete DOSE score available; *n* (%)^a^	8047 (77.4)	8047 (77.4)	7228 (69.5)	7228 (69.5)	5264 (50.6)	5264 (50.6)
Median DOSE score (IQR)	1 (1,2)	1 (1,2)	1 (1,2)	1 (1,3)	1 (1,3)	1 (1,3)
Patients with DOSE score change; *n* (%)^b^	–	166 (2.1)	–	179 (2.5)	–	132 (2.5)
Patients in low-risk group (≤3 points), *n* (%)^b^	7298 (90.7)	7258 (90.2)	6432 (89.0)	6405 (88.6)	4635 (88.1)	4602 (87.4)
Patients in moderate-risk group (4–5 points), *n* (%)^b^	680 (8.5)	710 (8.8)	715 (9.9)	730 (10.1)	560 (10.6)	583 (11.1)
Patients in high-risk group (≥6 points), *n* (%)^b^	69 (0.9)	79 (1.0)	81 (1.1)	93 (1.3)	69 (1.3)	79 (1.5)
Patients with risk group change, *n* (%)^b^	–	50 (0.6)	–	39 (0.5)	–	43 (0.8)

*SaPC* stand-alone primary care data, *PC/SC* linked primary care/secondary care data

^a^Of cohort, *N* = 10,393

^b^Of patients with complete DOSE score at instance

## Discussion

We aimed to develop a database algorithm based on the DOSE index to be used for primary care risk stratification of patients with COPD. Having successfully generated at least one DOSE score over a 5-year period for 8047 patients, we have demonstrated that, from a technical standpoint, algorithmic calculation of the index is possible using routinely collected primary care data. Furthermore, having generated three scores for 5264 patients, our results reveal regular recording of the complete suite of DOSE data items, but suggest that records were incomplete or inconsistent for almost half of our cohort of 10,393 patients. We also confirm previous research indicating incomplete recording of secondary care events in the primary care EHR,^22,23^ having observed a reduced exacerbation incidence from SaPC data compared to PC/SC data. However, the non-significant difference by data type suggests that, when considered at scale across a practice population, the DOSE index is robust to omission of a proportion of secondary care exacerbations. Nonetheless, their absence did curtail the accuracy of the algorithm for a small number of individual patients. Data linkage to reclaim missing events from secondary care data resulted in unchanged risk group allocation in most cases, although up to 0.8% of patients were found to have been misclassified and up to 2.5% of risk scores understated.

Risk stratification is appealing in the context of financial constraint in the NHS, having potential to facilitate more effective targeting of limited resources to those patients with the greatest need, whilst also guiding clinical decision-making to maintain a high quality of care for all. Widespread implementation to support primary care management of high prevalence chronic conditions could contribute to improved patient outcomes and reduced risk of hospitalisation, thereby underpinning recent NHS policies emphasising preventative care out of hospital.^3–5^ Among several multidimensional indices, the DOSE index was adopted for this study for its stated aim to guide the routine clinical management of COPD,^10^ its operational simplicity, ability to predict a range of outcomes and the clinical relevance of its components; a series of attributes marking it as particularly well-suited to real-world application in the primary care setting.^24^ Previous studies evaluating its predictive power in small primary and/or secondary care cohorts have shown encouraging results,^10–15^ but our study also demonstrates that it could form the basis of a risk stratification algorithm for use in the primary care sector.

The case for more proactive use of routinely collected clinical data is compelling. Indeed, the recent NHS Long Term Plan cites numerous benefits for clinicians, patients and the health system that may be achievable via use of innovative decision-support, predictive modelling and population health management applications.^5^ In principle, an automated risk stratification application based on the DOSE index could be a simple and practical means of incorporating risk stratification into the routine care of patients with COPD, evaluating data held in the primary care EHR to regularly output a risk score for all diagnosed patients. Seamless integration with the practice management system would have the additional advantages of ease of use for clinicians and enabling holistic assessment of other patient characteristics and risk factors alongside the core application.^25^

Nonetheless, our results underscore known limitations associated with the secondary use of clinical data. Individual records reflect clinician judgment on the best way to code clinical consultations, events, diagnoses and outcomes, with little standardisation or quality control at the point of entry.^26,27^ Previous studies have suggested that primary care coding is of sufficient quality to identify prevalent diagnosis of COPD^28^ and most chronic conditions,^29^ albeit variable across practices.^30^ We identified our patient cohort using an objectively defined set of diagnosis codes (sourced from the ‘Quality and Outcomes Framework’ incentive scheme^31,32^), but found that subsequent routine spirometry, dyspnoea and smoking status were inconsistent or absent from many patient records throughout the study period, suggesting a systematic influence upon data completeness. This may, for example, reflect a group of diagnosed patients having sporadic, little or no contact with their practice; recent research has posited a link between disease severity and data completeness, whereby the most severe patients’ more frequent encounters with the health system present more opportunity for clinical investigations and diagnoses.^33,34^ Equally, it may reveal a disparity in the relative simplicity of recording clinical diagnoses against greater variability in the recording of repeated routine care events, which may be obscured if recorded using free text,^20^ synonym codes or less specific alternatives.^26^ Terminological changes or changing patterns of clinical or coding behaviour may also drive variation over time.^21^ Ostensive absence from the record might reasonably be assumed to signify non-occurrence but, in reality, events may have been recorded using a non-standard and unanticipated method; it has been noted, for example, that even in primary care alone diverse strategies beyond the gold standard codes are used to record exacerbations.^35^ Moreover, although inpatient exacerbations are generally coded as the primary diagnosis in the hospital setting,^22^ any deviation may further complicate subsequent translation to the primary care EHR. Although others have recommended the use of a composite definition bridging primary and secondary care data to be confident of identifying all exacerbations,^22,36^ this approach is not viable when working exclusively within the primary care sector, where linked, patient-level secondary care data are rarely available.

A key strength of this study was the use of real-world clinical data generated by many users and practices using a selection of practice management systems. We therefore believe that our observations concerning data quality and completeness are likely to be generalisable to the wider primary care sector in England, and that our algorithm is reproducible using any similarly structured primary care database. Linkage to secondary care data was a further strength, without which it would not have been possible to quantify the shortfall of secondary care exacerbations in the primary care EHR nor demonstrate that, as the effect on risk scores is generally small, linked data are useful, but not essential for this purpose. However, we also recognise limitations arising from the inherently challenging nature of analysing data originally generated for patient care,^21^ where the principal objective of clinical recording is to best describe the patient’s condition so as to facilitate effective ongoing care. We benefitted from having both clinical and informatics expertise in our team but, all the same, were distant from the real-world clinical activity described by the data, limiting post-hoc understanding and interpretation of coded records. Having adopted an inclusive approach to specifying the algorithm to accommodate known data-quality issues, we may have introduced inaccuracies and false positives; we cannot exclude the possibility, for example, that our definitional criteria for primary care exacerbations may be met by a ‘rescue pack’ prescription for respiratory antibiotics and/or oral corticosteroids. Such issues could be alleviated by embedding routine secondary data analysis in primary care and directly involving clinicians throughout the process, an approach that would also facilitate resolution of specific data-quality issues via clinical review and audit of patient notes.

Our analysis was designed to assess the viability of the DOSE index in the context of data quality and completeness, rather than its clinical utility. Excluding patients known to have died during the study period is likely to have biased DOSE scores by also excluding many of the patients with greatest disease severity at baseline. While our analysis could be interpreted as describing the likelihood of access, at a given point in time, to a risk score of less than 1 year old, a direct measure of the recentness of the score may have more clinical relevance. Further pragmatic studies are also needed to evaluate the benefits of risk stratification in routine practice, determine whether risk scores are modifiable by clinical intervention, and clarify the optimal configuration of any primary care service developments that might utilise such an approach.

In conclusion, although our results show that a substantial *quantity* of data are already generated and held by the primary care sector and that, in general, regular recording of DOSE data items already occurs as a product of routine care, they also suggest a continued need to improve data *quality*. Irrespective of the cause, any systematic failure of data quality may threaten the integrity of complex algorithms based on multiple event-based predicates. The composition of the DOSE index, for example, carries the possibility that missing data could artificially depress risk scores which may, in turn, prompt inappropriate clinical action. A high standard of data quality and completeness in the EHR is, therefore, fundamental to any secondary application.^37^ We recommend that secondary use of primary care data be allied with the introduction of policies for improved and standardised data collection, regular within-practice audit of data quality, and a strengthening of the link between primary and secondary care data recording. Adoption of a comprehensive programme of data-quality improvement and secondary data analysis in the primary care sector would necessitate considerable upfront investment, to be sure, but may be offset by the opportunity for more cost-effective care in the longer term, in addition to the other benefits that could be achieved for clinicians, patients and the wider health system.

## Methods

### Study

This was a secondary analysis of data collected for a retrospective observational cohort study of patients with primary care diagnosis of COPD, using the Care and Health Information Analytics database (CHIA). Governance approval to access the source data was obtained from the Care and Health Information Exchange Information Governance Group (CHIEIGG). As this was a secondary analysis of anonymised data, written informed consent was not provided by study participants, and local research governance (University of Southampton Research, Integrity and Governance Team) confirmed at the time the study commenced that formal ethical approval was not required.

### Setting

CHIA is a patient-centric, anonymised database of routinely collected clinical data from participating healthcare providers in the counties of Hampshire and the Isle of Wight, southern England. At the time of this study, the database linked Read coded clinical entries describing patient care in the primary care setting^38^ to secondary care inpatient, outpatient and emergency department activity sourced from the Secondary Uses Service dataset.^39^ Data from 146 practices were available, representing around 1.4 million patients, or around 75% of the local practice and patient population. Missing practices were dispersed across the catchment area, with varied rural/urban classification, socioeconomic deprivation and patient composition. We are not aware of any systematic differences to those practices whose data were present.

### Participants

We identified a cohort of patients aged 18 or over with a Read coded primary care diagnosis of COPD as at 1 January 2010, and followed them across primary and secondary care settings over the 5 years to 31 December 2014. We excluded patients known to have died or relocated outside of the CHIA catchment area during the study period, as these events would inevitably result in a discontinuity in longitudinal data completeness.

### Identifying exacerbations

Definitional variations have been shown to impact upon observed exacerbation frequency.^40^ Their retrospective identification using routine clinical data adds further complexity in operationalising multiple diagnostic criteria^35^ and interpreting their longitudinal time course.^41^ We applied four definitions:

i. Acute exacerbation of COPD in the primary care record;
ii. COPD symptom or restated diagnosis in the primary care record within 7 days before or after a prescription for respiratory antibiotic or oral corticosteroid;
iii. COPD-related non-elective inpatient admission in the secondary care record;
iv. Respiratory-related emergency department attendance in the secondary care record.

Two separate exacerbation time series were created: the first consisting only of events identified using SaPC data under definitions (i) and (ii); the second of events identified using PC/SC data and all definitions. Importantly, definition (i) does not necessarily denote presentation to primary care; hospitalisations retrospectively recorded in the primary care EHR may also be identified in this manner.

All events recorded within a continuous 21-day period were interpreted as describing a single exacerbation, with the initial event taken as the incident date.

### Compiling the DOSE index

Further exposition of our methodology and a complete list of the codes used to identify relevant clinical events are included in the supplementary material. Briefly, Dyspnoea was assessed using the Medical Research Council (MRC) dyspnoea scale, Obstruction using the percent predicted forced expiratory volume in 1 s (FEV_1_%), Smoking was represented by reported smoking status, and Exacerbation by their number in the preceding year (defined as 365 days). Components were individually scored and aggregated to create the composite index, which has maximum range 0–8 points (Table 1).^10^ Scores were then stratified into three risk groups, at ≤ 3 (low risk), 4–5 (moderate risk) and ≥6 points (high risk).^18^

Using Microsoft SQL Server 2008 R2 (Microsoft, Redmond, WA, USA), we created an algorithm to assemble the relevant clinical records and calculate the DOSE score within the source database. The algorithm specification took Obstruction as the qualifying component. Patients required at least one valid FEV_1_% record for entry, which was then used as the index record against which the other DOSE components were benchmarked. The first DOSE score instance was associated with the earliest qualifying FEV_1_% record during the study period, and could take any date between 1 January 2010 and 31 December 2014. Subsequent instances were associated with the earliest qualifying FEV_1_% record a minimum of 1 year after the previous and a maximum of 4 years after the first. Each patient was limited to a maximum of three repeated instances over the study period. Thereafter, for Dyspnoea and Smoking components, any MRC dyspnoea scale and smoking status record occurring on the same date as the FEV_1_% record was selected. If absent, the closest record within the preceding year was used. For Exacerbation, the number of exacerbations recorded during the year preceding the FEV_1_% record was counted separately using SaPC and PC/SC data types. Thus, two DOSE scores were calculated at each instance, differentiated only by the data type used to identify exacerbations. Owing to the composition of the scoring system, the absence of secondary care exacerbations from the primary care EHR may have no effect, or may result in the final score calculated using PC/SC data being up to two points (and one risk group) greater than that using SaPC data.

### Analysis

The proportion of patients for whom Obstruction, Dyspnoea and Smoking components and the composite DOSE score were available was calculated at each instance. The proportion of patients with a non-zero Exacerbation score was compared by data type; as a valid input for this component, a zero value cannot preclude calculation of the DOSE index but could artificially reduce the final score.

Patients were characterised at baseline using categorical variables for patient age, sex, socioeconomic status (measured by index of multiple deprivation^42^), number of diagnosed comorbidities, and severity of airflow obstruction in COPD. As the distribution of diagnosed comorbidities was right skewed, category boundaries were set for clinical relevance and approximate equality of group sizes. Severity of airflow obstruction in COPD was represented by the closest FEV_1_% value within 1 year before or after 1 January 2010, and categorised by GOLD classification.^43^ Again, the proportion of patients in each category for whom a composite DOSE score was available was calculated at each instance.

As all DOSE score distributions were right skewed, the two-sided Mann–Whitney test was used to assess the statistical significance of differences by data type. The number and proportion of patients for whom the calculated DOSE score or risk group allocation changed by data type was reported.

Statistical analyses were undertaken using Stata SE Version 14.0 (StataCorp, College Station, TX, USA).

## Supplementary Information

Supplementary Information

## Data Availability

Individual level data used for this study are held within the Care and Health Information Analytics database safe haven environment and are not available to be shared. These governance rules also apply to derived individual data created during the analyses for this research. The approach taken for the study is detailed in the main text and supplementary information, and could be reproduced in any similarly structured linked database.

## Code availability

No custom software was developed; only standard use of existing routines within software for data manipulation and statistical analysis was employed. The approach taken for the development of the algorithm described in this study is based on publicly available guidance, as detailed in the main text and supplementary information.
