# Supplementary material for: Primary care risk stratification in COPD using routinely collected data: a secondary data analysis
Source: NPJ Prim Care Respir Med. 2019 Dec 4;29:42. doi: 10.1038/s41533-019-0154-6 (PMC6892877; doi:10.1038/s41533-019-0154-6)
Supplement: Supplementary file 1 — Supplementary Information [file 41533_2019_154_MOESM1_ESM.pdf]

Supplementary Material

Read codes

Read codes are widely used to describe routine clinical activity taking place in UK primary care, including diagnoses, symptoms, examination findings, investigations, administration, procedures and medications [1]. As our analysis used primary care data sourced from general practices recording clinical activity using the Read v2 coding system, all of the codes listed in this supplementary material are based on this system unless otherwise specified. A number of general practices included in this analysis use practice management systems provided by EMIS Health, within which a unique set of customised codes are used to record medications, most of which have an analogous Read v2 counterpart. Where appropriate we have listed both the Read v2 and EMIS codes used.

No practices using the CTV3 coding system were included in this analysis, and so no CTV3 codes are listed. However, code mapping tables are publicly available [2] to facilitate migration to this system, as well as future adoption of the SNOMED CT coding system in primary care.

Cohort definition

We identified our COPD cohort based on the presence of selected diagnostic Read codes in the patient's primary care record prior to 1st January 2010. We did not include any 'process of care' codes (for symptoms, signs, management and administration) nor prescribed medication codes consistent with COPD. We included three codes indicative of either acute exacerbation of COPD or COPD with acute lower respiratory tract infection (H3122, H3y0. and H3y1.) that are excluded by the 'Quality and Outcomes Framework' incentive scheme [3,4].

Code	Description
H3...	Chronic obstructive pulm.dis.
H31..	Chronic bronchitis
H310.	Simple chronic bronchitis
H3100	Chronic catarrhal bronchitis
H310z	Simple chronic bronchitis NOS
H311.	Mucopurulent chr.bronchitis
H3110	Purulent chronic bronchitis
H3111	Fetid chronic bronchitis
H311z	Mucopurulent chr.bronchit.NOS
H312.	Obstructive chronic bronchitis
H3120	Chronic asthmatic bronchitis
H3121	Emphysematous bronchitis
H3122	Acute exacerbation of COAD
H3123	Bronchiolitis obliterans
H312z	Obstructive chr.bronchitis NOS
H313.	Mixd simp+mucopur chron bronch
H31y.	Other chronic bronchitis
H31y1	Chronic tracheobronchitis
H31yz	Other chronic bronchitis NOS
H31z.	Chronic bronchitis NOS
H32..	Emphysema
H320.	Chronic bullous emphysema
H3200	Segmental bullous emphysema
H3201	Zonal bullous emphysema
H3202	Giant bullous emphysema
H3203	Bullous emphysema + collapse
H320z	Chronic bullous emphysema NOS

H321.	Panlobular emphysema
H322.	Centrilobular emphysema
H32y.	Other emphysema
H32y0	Acute vesicular emphysema
H32y1	Atrophic (senile) emphysema
H32y2	MacLeod's unilateral emphysema
H32yz	Other emphysema NOS
H32z.	Emphysema NOS
H36..	Mild chron obstr pulm disease
H37..	Mod chron obstr pulm disease
H38..	Sev chron obstr pulm disease
H39..	Very severe COPD
H3A..	End stag chron obst airway dis
H3y..	Chronic obstr.airway dis.OS
H3y0.	Chr obs pulm dis+ac l resp inf
H3y1.	Chr obs pulm dis+ac exac
H3z..	Chronic obstr.airway dis.NOS
H4640	Chronic chemical emphysema
H4641	Chemical obliter.bronchiolitis
Hyu30	[X]Other emphysema
Hyu31	[X]O spcf chron obs pulmon dis

Identifying COPD exacerbations

Four separate definitions bridging primary and secondary care data sources were used to identify COPD exacerbation events:

i) Acute COPD exacerbation in the patient's primary care record;
ii) COPD symptom or restated diagnosis in the patient's primary care record within seven days before or after a respiratory antibiotic or oral corticosteroid prescription;
iii) COPD-related non-elective inpatient admission in the patient's secondary care record;
iv) Respiratory-related emergency department attendance in the patient's secondary care record.

Secondary care non-elective inpatient and emergency department (ED) activity was sourced from the Secondary Uses Service dataset [5]. COPD-related diagnosis codes from the tenth revision of the International Classification of Diseases (ICD10) [6] were used to identify inpatient exacerbation events (where recorded in the primary or first, second or third secondary diagnosis positions) and some ED events. Non-elective admissions were defined as those with an admission method of '21', '22', '23', '24' or '2A' [7]. The remaining ED exacerbations were identified using the respiratory-related Accident and Emergency Diagnosis code '25' (where recorded in either first or second diagnosis positions) [8].

Two separate exacerbation time series were created: the first consisting only of events identified using stand-alone primary care (SaPC) data under definitions (i) and (ii); the second of events identified using linked primary care/secondary care (PC/SC) data and all definitions. Commencing from 1st January 2010, all events occurring within a continuous 21 day period were interpreted as describing a single exacerbation episode, with the initial event taken as the incident date. The next event occurring on or after the 22nd day was taken as the incident event of a new episode.

Code	Description	Definition
H3122	Acute exacerbation of COAD	(i) Acute COPD exacerbation
H3y1.	Chr obs pulm dis+ac exac	(i) Acute COPD exacerbation
16L..	Influenza-like symptoms	(ii) COPD symptom or diagnosis
171..	Cough	(ii) COPD symptom or diagnosis
1713.	Productive cough -clear sputum	(ii) COPD symptom or diagnosis

1714.	Productive cough -green sputum	(ii) COPD symptom or diagnosis
1715.	Productive cough-yellow sputum	(ii) COPD symptom or diagnosis
1716.	Productive cough NOS	(ii) COPD symptom or diagnosis
1717.	Night cough present	(ii) COPD symptom or diagnosis
1719.	Chesty cough	(ii) COPD symptom or diagnosis
171A.	Chronic cough	(ii) COPD symptom or diagnosis
171B.	Persistent cough	(ii) COPD symptom or diagnosis
171C.	Morning cough	(ii) COPD symptom or diagnosis
171D.	Evening cough	(ii) COPD symptom or diagnosis
171F.	Cough with fever	(ii) COPD symptom or diagnosis
171H.	Difficulty coughing up sputum	(ii) COPD symptom or diagnosis
171L.	Cough on exercise	(ii) COPD symptom or diagnosis
171Z.	Cough symptom NOS	(ii) COPD symptom or diagnosis
172..	Blood in sputum - haemoptysis	(ii) COPD symptom or diagnosis
173..	Breathlessness	(ii) COPD symptom or diagnosis
1732.	Breathless - moderate exertion	(ii) COPD symptom or diagnosis
1733.	Breathless - mild exertion	(ii) COPD symptom or diagnosis
1734.	Breathless - at rest	(ii) COPD symptom or diagnosis
1735.	Breathless - lying flat	(ii) COPD symptom or diagnosis
1737.	Wheezing	(ii) COPD symptom or diagnosis
1738.	Difficulty breathing	(ii) COPD symptom or diagnosis
1739.	Shortness of breath	(ii) COPD symptom or diagnosis
173b.	Unab compl sentence one breath	(ii) COPD symptom or diagnosis
173B.	Nocturnal cough / wheeze	(ii) COPD symptom or diagnosis
173C.	Short of breath on exertion	(ii) COPD symptom or diagnosis
173D.	Nocturnal dyspnoea	(ii) COPD symptom or diagnosis
173F.	SOB dressing/undressing	(ii) COPD symptom or diagnosis
173g.	Breathlessness csg diff eating	(ii) COPD symptom or diagnosis
173G.	Breathless - strenuous exertn	(ii) COPD symptom or diagnosis
173Z.	Breathlessness NOS	(ii) COPD symptom or diagnosis
189..	Worsening exercise tolerance	(ii) COPD symptom or diagnosis
1W0..	Possible influenza A vir H1N1	(ii) COPD symptom or diagnosis
2322.	O/E - dyspnoea	(ii) COPD symptom or diagnosis
2324.	O/E - respiratory distress	(ii) COPD symptom or diagnosis
2DE3.	O/E - respiratory obstruction	(ii) COPD symptom or diagnosis
41D4.	Sputum sample obtained	(ii) COPD symptom or diagnosis
4E...	Sputum examination	(ii) COPD symptom or diagnosis
4E1..	Sputum examination - general	(ii) COPD symptom or diagnosis
4E11.	Sputum sent for examination	(ii) COPD symptom or diagnosis
4E13.	Sputum examination: abnormal	(ii) COPD symptom or diagnosis
4E14.	Sputum - not infected	(ii) COPD symptom or diagnosis
4E1Z.	Sputum gen. exam. NOS	(ii) COPD symptom or diagnosis
4E2..	Sputum inspection	(ii) COPD symptom or diagnosis
4E21.	Sputum appears normal	(ii) COPD symptom or diagnosis
4E22.	Sputum: excessive - mucoid	(ii) COPD symptom or diagnosis
4E23.	Sputum: mucopurulent	(ii) COPD symptom or diagnosis
4E24.	Sputum: contains blood	(ii) COPD symptom or diagnosis
4E25.	Sputum: frothy/watery	(ii) COPD symptom or diagnosis
4E26.	Sputum: fetid/offensive	(ii) COPD symptom or diagnosis
4E27.	Clear sputum	(ii) COPD symptom or diagnosis
4E28.	Yellow sputum	(ii) COPD symptom or diagnosis
4E29.	Green sputum	(ii) COPD symptom or diagnosis
4E290	Dark green sputum	(ii) COPD symptom or diagnosis
4E291	Pale green sputum	(ii) COPD symptom or diagnosis
4E2A.	Sputum appearance	(ii) COPD symptom or diagnosis
4E2C.	Brown sputum	(ii) COPD symptom or diagnosis
4E2D.	White sputum	(ii) COPD symptom or diagnosis
4E2E.	Volume of sputum	(ii) COPD symptom or diagnosis

4E2E0	Copious sputum	(ii) COPD symptom or diagnosis
4E2E1	Moderate sputum	(ii) COPD symptom or diagnosis
4E2E3	Scanty sputum	(ii) COPD symptom or diagnosis
4E2F.	Grey sputum	(ii) COPD symptom or diagnosis
4E2G.	Bloodstained sputum	(ii) COPD symptom or diagnosis
4E2Z.	Sputum inspection NOS	(ii) COPD symptom or diagnosis
4E3..	Sputum microscopy	(ii) COPD symptom or diagnosis
4E36.	Sputum: pus cells present	(ii) COPD symptom or diagnosis
4E37.	Sputum: organism on gram stain	(ii) COPD symptom or diagnosis
4E3Z.	Sputum microscopy NOS	(ii) COPD symptom or diagnosis
4E4..	Sputum culture	(ii) COPD symptom or diagnosis
4EZ..	Sputum examination NOS	(ii) COPD symptom or diagnosis
4I1E.	Respiratory MC&S	(ii) COPD symptom or diagnosis
4I2F.	Lower respiratory sample	(ii) COPD symptom or diagnosis
4JF5.	Sputum sent for C/S	(ii) COPD symptom or diagnosis
6635.	Increasing exercise wheeze	(ii) COPD symptom or diagnosis
663F.	Oral steroids started	(ii) COPD symptom or diagnosis
663L.	Bronchodilators used > 1 /day	(ii) COPD symptom or diagnosis
66Yg.	COPD disturbs sleep	(ii) COPD symptom or diagnosis
8BP8.	AB therapy acute pulmon exacer	(ii) COPD symptom or diagnosis
8H2R.	Admit COPD emergency	(ii) COPD symptom or diagnosis
8H7j.	Refer respir rapid respon team	(ii) COPD symptom or diagnosis
H0...	Acute respiratory infections	(ii) COPD symptom or diagnosis
H05..	Other acute upper resp.infect.	(ii) COPD symptom or diagnosis
H051.	Acute up resp tract infection	(ii) COPD symptom or diagnosis
H05z.	Upper respiratory infect.NOS	(ii) COPD symptom or diagnosis
H06..	Acute bronchitis/bronchiolitis	(ii) COPD symptom or diagnosis
H060.	Acute bronchitis	(ii) COPD symptom or diagnosis
H0600	Acute fibrinous bronchitis	(ii) COPD symptom or diagnosis
H0601	Acute membranous bronchitis	(ii) COPD symptom or diagnosis
H0602	Acute pseudomembranous bronch.	(ii) COPD symptom or diagnosis
H0603	Acute purulent bronchitis	(ii) COPD symptom or diagnosis
H0604	Acute croupous bronchitis	(ii) COPD symptom or diagnosis
H0605	Acute tracheobronchitis	(ii) COPD symptom or diagnosis
H0606	Acute pneumococcal bronchitis	(ii) COPD symptom or diagnosis
H0607	Acute streptococcal bronchitis	(ii) COPD symptom or diagnosis
H0608	Acute H.influenzae bronchitis	(ii) COPD symptom or diagnosis
H060A	Ac bronch/mycoplasma pneumonia	(ii) COPD symptom or diagnosis
H060B	Acut bronch due coxsackievirus	(ii) COPD symptom or diagnosis
H060C	Acut bronch/parainfluenza vir	(ii) COPD symptom or diagnosis
H060D	Acut bronch/resp syncytial vir	(ii) COPD symptom or diagnosis
H060E	Acute bronchitis/rhinovirus	(ii) COPD symptom or diagnosis
H060F	Acute bronchitis/echovirus	(ii) COPD symptom or diagnosis
H060v	Subacute bronchitis unspecif.	(ii) COPD symptom or diagnosis
H060w	Acute viral bronchitis unspec.	(ii) COPD symptom or diagnosis
H060x	Acute bact.bronchitis unspec.	(ii) COPD symptom or diagnosis
H060z	Acute bronchitis NOS	(ii) COPD symptom or diagnosis
H062.	Acute low respitract infection	(ii) COPD symptom or diagnosis
H06z.	Acute bronchitis/bronchiol.NOS	(ii) COPD symptom or diagnosis
H06z0	Chest infection NOS	(ii) COPD symptom or diagnosis
H06z1	Lower resp tract infection	(ii) COPD symptom or diagnosis
H06z2	Recurrent chest infection	(ii) COPD symptom or diagnosis
H07..	Chest cold	(ii) COPD symptom or diagnosis
H0y..	Acute respiratory infectns.OS	(ii) COPD symptom or diagnosis
H0z..	Acute respiratory infectn.NOS	(ii) COPD symptom or diagnosis
H27..	Influenza	(ii) COPD symptom or diagnosis
H271.	Influenza + other resp.manif.	(ii) COPD symptom or diagnosis
H2710	Influenza + laryngitis	(ii) COPD symptom or diagnosis

H2711	Influenza + pharyngitis	(ii) COPD symptom or diagnosis
H271z	Influenza + resp.manifest.NOS	(ii) COPD symptom or diagnosis
H27y.	Influenza + other manifestat.	(ii) COPD symptom or diagnosis
H27y0	Influenza + encephalopathy	(ii) COPD symptom or diagnosis
H27y1	Influenza + GIT involvement	(ii) COPD symptom or diagnosis
H27yz	Influenza + other manifest.NOS	(ii) COPD symptom or diagnosis
H27z.	Influenza NOS	(ii) COPD symptom or diagnosis
H3...	Chronic obstructive pulm.dis.	(ii) COPD symptom or diagnosis
H30..	Bronchitis unspecified	(ii) COPD symptom or diagnosis
H300.	Tracheobronchitis NOS	(ii) COPD symptom or diagnosis
H301.	Laryngotracheobronchitis	(ii) COPD symptom or diagnosis
H302.	Wheezy bronchitis	(ii) COPD symptom or diagnosis
H30z.	Bronchitis NOS	(ii) COPD symptom or diagnosis
H31..	Chronic bronchitis	(ii) COPD symptom or diagnosis
H310.	Simple chronic bronchitis	(ii) COPD symptom or diagnosis
H3100	Chronic catarrhal bronchitis	(ii) COPD symptom or diagnosis
H3101	Smokers' cough	(ii) COPD symptom or diagnosis
H310z	Simple chronic bronchitis NOS	(ii) COPD symptom or diagnosis
H311.	Mucopurulent chr.bronchitis	(ii) COPD symptom or diagnosis
H3110	Purulent chronic bronchitis	(ii) COPD symptom or diagnosis
H3111	Fetid chronic bronchitis	(ii) COPD symptom or diagnosis
H311z	Mucopurulent chr.bronchit.NOS	(ii) COPD symptom or diagnosis
H312.	Obstructive chronic bronchitis	(ii) COPD symptom or diagnosis
H3120	Chronic asthmatic bronchitis	(ii) COPD symptom or diagnosis
H3121	Emphysematous bronchitis	(ii) COPD symptom or diagnosis
H3122	Acute exacerbation of COAD	(ii) COPD symptom or diagnosis
H3123	Bronchiolitis obliterans	(ii) COPD symptom or diagnosis
H312z	Obstructive chr.bronchitis NOS	(ii) COPD symptom or diagnosis
H313.	Mixd simp+mucopur chron bronch	(ii) COPD symptom or diagnosis
H31y.	Other chronic bronchitis	(ii) COPD symptom or diagnosis
H31y1	Chronic tracheobronchitis	(ii) COPD symptom or diagnosis
H31yz	Other chronic bronchitis NOS	(ii) COPD symptom or diagnosis
H31z.	Chronic bronchitis NOS	(ii) COPD symptom or diagnosis
H32..	Emphysema	(ii) COPD symptom or diagnosis
H320.	Chronic bullous emphysema	(ii) COPD symptom or diagnosis
H3200	Segmental bullous emphysema	(ii) COPD symptom or diagnosis
H3201	Zonal bullous emphysema	(ii) COPD symptom or diagnosis
H3202	Giant bullous emphysema	(ii) COPD symptom or diagnosis
H3203	Bullous emphysema + collapse	(ii) COPD symptom or diagnosis
H320z	Chronic bullous emphysema NOS	(ii) COPD symptom or diagnosis
H321.	Panlobular emphysema	(ii) COPD symptom or diagnosis
H322.	Centrilobular emphysema	(ii) COPD symptom or diagnosis
H32y.	Other emphysema	(ii) COPD symptom or diagnosis
H32y0	Acute vesicular emphysema	(ii) COPD symptom or diagnosis
H32y1	Atrophic (senile) emphysema	(ii) COPD symptom or diagnosis
H32y2	MacLeod's unilateral emphysema	(ii) COPD symptom or diagnosis
H32yz	Other emphysema NOS	(ii) COPD symptom or diagnosis
H32z.	Emphysema NOS	(ii) COPD symptom or diagnosis
H36..	Mild chron obstr pulm disease	(ii) COPD symptom or diagnosis
H37..	Mod chron obstr pulm disease	(ii) COPD symptom or diagnosis
H38..	Sev chron obstr pulm disease	(ii) COPD symptom or diagnosis
H39..	Very severe COPD	(ii) COPD symptom or diagnosis
H3A..	End stag chron obst airway dis	(ii) COPD symptom or diagnosis
H3y..	Chronic obstr.airway dis.OS	(ii) COPD symptom or diagnosis
H3y0.	Chr obs pulm dis+ac l resp inf	(ii) COPD symptom or diagnosis
H3y1.	Chr obs pulm dis+ac exac	(ii) COPD symptom or diagnosis
H3z..	Chronic obstr.airway dis.NOS	(ii) COPD symptom or diagnosis
H460.	Chemical bronchitis/pneumonit.	(ii) COPD symptom or diagnosis

H4600	Acute chemical bronchitis	(ii) COPD symptom or diagnosis
H460z	Chemical bronch/pneumonit NOS	(ii) COPD symptom or diagnosis
H4640	Chronic chemical emphysema	(ii) COPD symptom or diagnosis
H4641	Chemical obliter.bronchiolitis	(ii) COPD symptom or diagnosis
H59..	Respiratory failure	(ii) COPD symptom or diagnosis
H590.	Acute respiratory failure	(ii) COPD symptom or diagnosis
H591.	Chronic respiratory failure	(ii) COPD symptom or diagnosis
H592.	Chronic type 1 respir failure	(ii) COPD symptom or diagnosis
H593.	Chronic type 2 respir failure	(ii) COPD symptom or diagnosis
Hyu0.	[X]Ac upp respiratory infectns	(ii) COPD symptom or diagnosis
Hyu04	[X]Flu+o rsp manif	(ii) COPD symptom or diagnosis
Hyu05	[X]Flu+o manifst	(ii) COPD symptom or diagnosis
Hyu06	[X]Flu+o rsp manif	(ii) COPD symptom or diagnosis
Hyu07	[X]Flu+o maniftns	(ii) COPD symptom or diagnosis
Hyu1.	[X]Oth acute lowr resp infects	(ii) COPD symptom or diagnosis
Hyu10	[X]Ac bronchitis/o spcf orgnsm	(ii) COPD symptom or diagnosis
Hyu11	[X]Ac bronchltis/o spcf orgnsm	(ii) COPD symptom or diagnosis
Hyu3.	[X]Chron lowr respiratory dis	(ii) COPD symptom or diagnosis
Hyu30	[X]Other emphysema	(ii) COPD symptom or diagnosis
Hyu31	[X]O spcf chron obs pulmon dis	(ii) COPD symptom or diagnosis
R06..	[D]Respiratory/chest symptoms	(ii) COPD symptom or diagnosis
R0600	[D]Respiratory symptom unspec.	(ii) COPD symptom or diagnosis
R0601	[D]Hyperventilation	(ii) COPD symptom or diagnosis
R0602	[D]Orthopnoea	(ii) COPD symptom or diagnosis
R0603	[D]Tachypnoea	(ii) COPD symptom or diagnosis
R0606	[D]Respiratory distress	(ii) COPD symptom or diagnosis
R0607	[D]Respiratory insufficiency	(ii) COPD symptom or diagnosis
R0608	[D]Shortness of breath	(ii) COPD symptom or diagnosis
R0609	[D]Wheezing	(ii) COPD symptom or diagnosis
R060A	[D]Dyspnoea	(ii) COPD symptom or diagnosis
R060D	[D]Breathlessness	(ii) COPD symptom or diagnosis
R060z	[D]Respiratory abnormalit.NOS	(ii) COPD symptom or diagnosis
R062.	[D]Cough	(ii) COPD symptom or diagnosis
R063.	[D]Haemoptysis	(ii) COPD symptom or diagnosis
R0630	[D]Cough with haemorrhage	(ii) COPD symptom or diagnosis
R063z	[D]Haemoptysis NOS	(ii) COPD symptom or diagnosis
R064.	[D]Abnormal sputum	(ii) COPD symptom or diagnosis
R0640	[D]Sputum abnormal - amount	(ii) COPD symptom or diagnosis
R0641	[D]Sputum abnormal - colour	(ii) COPD symptom or diagnosis
R0642	[D]Sputum abnormal - odour	(ii) COPD symptom or diagnosis
R0643	[D]Abnormal sputum - tenacious	(ii) COPD symptom or diagnosis
R064z	[D]Abnormal sputum NOS	(ii) COPD symptom or diagnosis
R0658	[D]Chest tightness	(ii) COPD symptom or diagnosis
R06z.	[D]Resp./chest symptoms-other	(ii) COPD symptom or diagnosis
R06zz	[D]Resp./chest symptoms NOS	(ii) COPD symptom or diagnosis
R1531	[D]Positive culture - sputum	(ii) COPD symptom or diagnosis
R2y1.	[D]Respiratory failure	(ii) COPD symptom or diagnosis
R2y10	[D]Cardiorespiratory failure	(ii) COPD symptom or diagnosis
R2y1z	[D]Respiratory failure NOS	(ii) COPD symptom or diagnosis
SP132	Post operative chest infection	(ii) COPD symptom or diagnosis
AMCA123	Amoxil Capsules 250 mg	(ii) Respiratory antibiotic (EMIS)
AMCA124	Amoxil Capsules 500 mg	(ii) Respiratory antibiotic (EMIS)
AMCA141	Ampicillin Capsules 250 mg	(ii) Respiratory antibiotic (EMIS)
AMCA142	Ampicillin Capsules 500 mg	(ii) Respiratory antibiotic (EMIS)
AMCA17510NEMIS	Amoxicillin Capsules 250 mg	(ii) Respiratory antibiotic (EMIS)
AMCA17511NEMIS	Amoxicillin Capsules 500 mg	(ii) Respiratory antibiotic (EMIS)
AMCA9025BRIDL	Amoram Capsules 250 mg	(ii) Respiratory antibiotic (EMIS)
AMCA9027BRIDL	Amoram Capsules 500 mg	(ii) Respiratory antibiotic (EMIS)

AMDI125	Amoxil Dispersible tablets 500 mg	(ii) Respiratory antibiotic (EMIS)
AMFI18005EMIS	Amoxil Fiztabs 125 mg	(ii) Respiratory antibiotic (EMIS)
AMFI18006EMIS	Amoxil Fiztabs 250 mg	(ii) Respiratory antibiotic (EMIS)
AMFI18007EMIS	Amoxil Fiztabs 500 mg	(ii) Respiratory antibiotic (EMIS)
AMIN9033BRIDL	Amoram Injection 250 mg/amp	(ii) Respiratory antibiotic (EMIS)
AMIN9035BRIDL	Amoram Injection 500 mg/amp	(ii) Respiratory antibiotic (EMIS)
AMMI143	Ampicillin Mixture 125 mg/5 ml	(ii) Respiratory antibiotic (EMIS)
AMMI144	Ampicillin Mixture 250 mg/5 ml	(ii) Respiratory antibiotic (EMIS)
AMOR17515NEMIS	Amoxicillin Oral suspension 125 mg/5 ml	(ii) Respiratory antibiotic (EMIS)
AMOR17516NEMIS	Amoxicillin Oral suspension 250 mg/5 ml	(ii) Respiratory antibiotic (EMIS)
AMOR17518NEMIS	Amoxicillin Oral Powder Sachets Sugar Free 3 gram sachet	(ii) Respiratory antibiotic (EMIS)
AMOR17519NEMIS	Amoxicillin Oral Suspension Sugar Free 125 mg/5 ml	(ii) Respiratory antibiotic (EMIS)
AMOR17520NEMIS	Amoxicillin Oral Suspension Sugar Free 250 mg/5 ml	(ii) Respiratory antibiotic (EMIS)
AMPA129	Amoxil Paediatric suspension 125 mg/1.25 ml	(ii) Respiratory antibiotic (EMIS)
AMPA17517NEMIS	Amoxicillin Paediatric oral suspension 125 mg/1.25 ml	(ii) Respiratory antibiotic (EMIS)
AMPA4595	Ampicillin Paediatric suspension 125 mg/1.25 ml	(ii) Respiratory antibiotic (EMIS)
AMPA4596	Ampicillin Paediatric tablets 125 mg	(ii) Respiratory antibiotic (EMIS)
AMSA130	Amoxil Sachets sf 3 gm	(ii) Respiratory antibiotic (EMIS)
AMSA131	Amoxil Sachets sf 750 mg	(ii) Respiratory antibiotic (EMIS)
AMSY132	Amoxil Syrup sf 125 mg/5 ml	(ii) Respiratory antibiotic (EMIS)
AMSY133	Amoxil Syrup sf 250 mg/5 ml	(ii) Respiratory antibiotic (EMIS)
AMSY4597	Ampicillin Syrup 125 mg/5 ml	(ii) Respiratory antibiotic (EMIS)
AMSY4598	Ampicillin Syrup forte 250 mg/5 ml	(ii) Respiratory antibiotic (EMIS)
AMSY9029BRIDL	Amoram Syrup 125 ml/5 ml	(ii) Respiratory antibiotic (EMIS)
AMSY9031BRIDL	Amoram Syrup 250 mg/5 ml	(ii) Respiratory antibiotic (EMIS)
AUSU19802EMIS	Augmentin 125/31 Sf Sugar free suspension 125 mg/5 ml + 31 mg/5 ml	(ii) Respiratory antibiotic (EMIS)
AUSU19805EMIS	Augmentin 250/62 Sf Sugar free suspension 250 mg/5 ml + 62 mg/5 ml	(ii) Respiratory antibiotic (EMIS)
AUSU27887EMIS	Augmentin-Duo 400/57 Sugar-free oral suspension 400 mg/5 ml + 57 mg/5 ml	(ii) Respiratory antibiotic (EMIS)
AUTA23527EMIS	Augmentin 375 Mg Tablets	(ii) Respiratory antibiotic (EMIS)
AUTA23530EMIS	Augmentin 625 Mg Tablets	(ii) Respiratory antibiotic (EMIS)
AUTA33618EMIS	Augmentin 1 G Tablets	(ii) Respiratory antibiotic (EMIS)
AVTA15045NEMIS	Avelox Tablets 400 mg	(ii) Respiratory antibiotic (EMIS)
AZCA1005EMIS	Azithromycin Capsules 250 mg	(ii) Respiratory antibiotic (EMIS)
AZSU9381EMIS	Azithromycin Suspension 200 mg/5 ml	(ii) Respiratory antibiotic (EMIS)
AZTA23020NEMIS	Azithromycin Tablets 250 mg	(ii) Respiratory antibiotic (EMIS)
AZTA34169EMIS	Azithromycin Tablets 500 mg	(ii) Respiratory antibiotic (EMIS)
CECA17567NEMIS	Cefalexin Capsules 250 mg	(ii) Respiratory antibiotic (EMIS)
CECA17568NEMIS	Cefalexin Capsules 500 mg	(ii) Respiratory antibiotic (EMIS)
CECA17575NEMIS	Cefradine Capsules 250 mg	(ii) Respiratory antibiotic (EMIS)
CECA17576NEMIS	Cefradine Capsules 500 mg	(ii) Respiratory antibiotic (EMIS)
CECA20695EMIS	Cefaclor Capsules 500 mg	(ii) Respiratory antibiotic (EMIS)
CECA4686	Cefaclor Capsules 250 mg	(ii) Respiratory antibiotic (EMIS)
CECA4688	Cefadroxil Capsules 500 mg	(ii) Respiratory antibiotic (EMIS)
CECA529	Ceporex Capsules 250 mg	(ii) Respiratory antibiotic (EMIS)
CECA530	Ceporex Capsules 500 mg	(ii) Respiratory antibiotic (EMIS)
CEM/22447EMIS	Cefaclor M/R tablets 375 mg	(ii) Respiratory antibiotic (EMIS)
CEM/27778EMIS	Cefaclor M/R tablets 500 mg	(ii) Respiratory antibiotic (EMIS)
CEOR17571NEMIS	Cefalexin Oral suspension 125 mg/5 ml	(ii) Respiratory antibiotic (EMIS)
CEOR17572NEMIS	Cefalexin Oral suspension 250 mg/5 ml	(ii) Respiratory antibiotic (EMIS)
CEOR17573NEMIS	Cefalexin Oral suspension 500 mg/5 ml	(ii) Respiratory antibiotic (EMIS)
CEOR17579NEMIS	Cefradine Oral solution 250 mg/5 ml	(ii) Respiratory antibiotic (EMIS)
CEOR28319NEMIS	Cefalexin Oral Suspension (Sugar-Free) 250 mg/5 ml	(ii) Respiratory antibiotic (EMIS)
CEOR44053NEMIS	Cefalexin 125mg/5ml oral suspension sugar free	(ii) Respiratory antibiotic (EMIS)
CEOR44053NEMIS	Cefalexin Oral Suspension (Sugar-Free) 125 mg/5 ml	(ii) Respiratory antibiotic (EMIS)
CEPA27534EMIS	Cefpodoxime Proxetil Paediatric suspension 40 mg/5 ml	(ii) Respiratory antibiotic (EMIS)
CEPA531	Ceporex Paediatric drops 125 mg/1.25 ml	(ii) Respiratory antibiotic (EMIS)
CESA20465EMIS	Cefuroxime Axetil Sachets 125 mg/sachet	(ii) Respiratory antibiotic (EMIS)
CESU10328HILLI	Cefixime Paediatric suspension 100 mg/5 ml	(ii) Respiratory antibiotic (EMIS)

CESU18066EMIS	Cefuroxime Axetil Suspension 125 mg/5 ml	(ii) Respiratory antibiotic (EMIS)
CESU31410EMIS	Cefaclor Sugar-free suspension 125 mg/5 ml	(ii) Respiratory antibiotic (EMIS)
CESU31411EMIS	Cefaclor Sugar-free suspension 250 mg/5 ml	(ii) Respiratory antibiotic (EMIS)
CESU4687	Cefaclor Suspension 125 mg/5 ml	(ii) Respiratory antibiotic (EMIS)
CESU4689	Cefadroxil Suspension 125 mg/5 ml	(ii) Respiratory antibiotic (EMIS)
CESU532	Ceporex Suspension 125 mg/5 ml	(ii) Respiratory antibiotic (EMIS)
CESU533	Ceporex Suspension 250 mg/5 ml	(ii) Respiratory antibiotic (EMIS)
CESU6799	Cefadroxil Suspension 250 mg/5 ml	(ii) Respiratory antibiotic (EMIS)
CESU6800	Cefadroxil Suspension 500 mg/5 ml	(ii) Respiratory antibiotic (EMIS)
CESU6870	Cefaclor Suspension 250 mg/5 ml	(ii) Respiratory antibiotic (EMIS)
CESY534	Ceporex Syrup 125 mg/5 ml	(ii) Respiratory antibiotic (EMIS)
CESY535	Ceporex Syrup 250 mg/5 ml	(ii) Respiratory antibiotic (EMIS)
CESY536	Ceporex Syrup 500 mg/5 ml	(ii) Respiratory antibiotic (EMIS)
CETA10329HILLI	Cefixime Tablets 200 mg	(ii) Respiratory antibiotic (EMIS)
CETA10999BRIDL	Cefuroxime Tablets 250 mg	(ii) Respiratory antibiotic (EMIS)
CETA14215NEMIS	Cefuroxime Tablets 500 mg	(ii) Respiratory antibiotic (EMIS)
CETA17569NEMIS	Cefalexin Tablets 250 mg	(ii) Respiratory antibiotic (EMIS)
CETA17570NEMIS	Cefalexin Tablets 500 mg	(ii) Respiratory antibiotic (EMIS)
CETA22410EMIS	Cefpodoxime Proxetil Tablets 100 mg	(ii) Respiratory antibiotic (EMIS)
CETA537	Ceporex Tablets 250 mg	(ii) Respiratory antibiotic (EMIS)
CETA538	Ceporex Tablets 500 mg	(ii) Respiratory antibiotic (EMIS)
CETA7121	Cefuroxime Tablets 125 mg	(ii) Respiratory antibiotic (EMIS)
CETA9505BRIDL	Ceporex Tablets 1g	(ii) Respiratory antibiotic (EMIS)
CHCA602	Chlortetracycline Hydrochloride Capsules 250 mg	(ii) Respiratory antibiotic (EMIS)
CISU32102EMIS	Ciproxin Suspension 250 mg/5 ml	(ii) Respiratory antibiotic (EMIS)
CISU32104EMIS	Ciprofloxacin Suspension 250 mg/5 ml	(ii) Respiratory antibiotic (EMIS)
CITA22180EMIS	Ciproxin Tablets 500 mg	(ii) Respiratory antibiotic (EMIS)
CITA22182EMIS	Ciprofloxacin Tablets 500 mg	(ii) Respiratory antibiotic (EMIS)
CITA23557EMIS	Ciproxin Tablets 750 mg	(ii) Respiratory antibiotic (EMIS)
CITA23848EMIS	Ciprofloxacin Tablets 750 mg	(ii) Respiratory antibiotic (EMIS)
CITA31080EMIS	Ciprofloxacin Tablets 100 mg	(ii) Respiratory antibiotic (EMIS)
CITA31082EMIS	Ciproxin Tablets 100 mg	(ii) Respiratory antibiotic (EMIS)
CITA5645	Ciproxin Tablets 250 mg	(ii) Respiratory antibiotic (EMIS)
CITA6845	Ciprofloxacin Tablets 250 mg	(ii) Respiratory antibiotic (EMIS)
CLGR21384NEMIS	Clarithromycin Granules For Oral Suspension Straws 125 mg	(ii) Respiratory antibiotic (EMIS)
CLGR21385NEMIS	Clarithromycin Granules For Oral Suspension Straws 187.5 mg	(ii) Respiratory antibiotic (EMIS)
CLGR21386NEMIS	Clarithromycin Granules For Oral Suspension Straws 250 mg	(ii) Respiratory antibiotic (EMIS)
CLM/30085EMIS	Clarithromycin M/R tablets 500 mg	(ii) Respiratory antibiotic (EMIS)
CLPA22477EMIS	Clarithromycin Paediatric suspension 125 mg/5 ml	(ii) Respiratory antibiotic (EMIS)
CLPA770NEMIS	Clarithromycin Paediatric suspension 250 mg/5 ml	(ii) Respiratory antibiotic (EMIS)
CLSA31718EMIS	Clarithromycin Sachets 250 mg/sachet	(ii) Respiratory antibiotic (EMIS)
CLTA26172EMIS	Clarithromycin Tablets 500 mg	(ii) Respiratory antibiotic (EMIS)
CLTA9105EMIS	Clarithromycin Tablets 250 mg	(ii) Respiratory antibiotic (EMIS)
COAD4763	Co-Trimoxazole Adult suspension 480 mg/5 ml	(ii) Respiratory antibiotic (EMIS)
COAD689	Co-Trimoxazole Mixture 480 mg/5 ml	(ii) Respiratory antibiotic (EMIS)
CODI29221EMIS	Co-Amoxiclav 250/125 Dispersible tablets Sugar Free	(ii) Respiratory antibiotic (EMIS)
CODI690	Co-Trimoxazole Dispersible tablets 480 mg	(ii) Respiratory antibiotic (EMIS)
CODR4764	Co-Trimoxazole Drapsules 480 mg	(ii) Respiratory antibiotic (EMIS)
COFO4765	Co-Trimoxazole Forte tablets dispersible 960 mg	(ii) Respiratory antibiotic (EMIS)
COFO691	Co-Trimoxazole Tablets 960 mg	(ii) Respiratory antibiotic (EMIS)
COOR43494NEMIS	Co-amoxiclav 125mg/31mg/5ml oral suspension	(ii) Respiratory antibiotic (EMIS)
COOR43494NEMIS	Co-Amoxiclav 125/31 Oral suspension 125 mg/5 ml + 31 mg/5 ml	(ii) Respiratory antibiotic (EMIS)
COOR43495NEMIS	Co-Amoxiclav 250/62 Oral Suspension 250 mg/5 ml + 62 mg/5 ml	(ii) Respiratory antibiotic (EMIS)
COOR43495NEMIS	Co-amoxiclav 250mg/62mg/5ml oral suspension	(ii) Respiratory antibiotic (EMIS)
COPA10270BRIDL	Co-Trimoxazole Paediatric s/f dispersible tablets 120 mg	(ii) Respiratory antibiotic (EMIS)
COPA3325	Co-Trimoxazole Paediatric mixture 240 mg/5 ml	(ii) Respiratory antibiotic (EMIS)
COPA4767	Co-Trimoxazole Paediatric suspension 240 mg/5 ml	(ii) Respiratory antibiotic (EMIS)
COPA695	Co-Trimoxazole Paediatric tablets 120 mg	(ii) Respiratory antibiotic (EMIS)

COSO3326	Co-Trimoxazole Solution 96 mg/ml	(ii) Respiratory antibiotic (EMIS)
COSU10268BRIDL	Co-Trimoxazole Sugar free paediatric syrup 240 mg/5 ml	(ii) Respiratory antibiotic (EMIS)
COSU19812EMIS	Co-Amoxiclav 125/31 Sugar free suspension 125 mg/5 ml + 31 mg/5 ml	(ii) Respiratory antibiotic (EMIS)
COSU19815EMIS	Co-Amoxiclav 250/62 Sugar free suspension 250 mg/5 ml + 62 mg/5 ml	(ii) Respiratory antibiotic (EMIS)
COSU27890EMIS	Co-Amoxiclav 400/57 Sugar-free oral suspension 400 mg/5 ml + 57 mg/5 ml	(ii) Respiratory antibiotic (EMIS)
COTA23533EMIS	Co-Amoxiclav 250/125 Tablets	(ii) Respiratory antibiotic (EMIS)
COTA23536EMIS	Co-Amoxiclav 500/125 Tablets	(ii) Respiratory antibiotic (EMIS)
COTA33621EMIS	Co-Amoxiclav 875/125 Tablets	(ii) Respiratory antibiotic (EMIS)
COTA4768	Co-Trimoxazole Tablets forte 960 mg	(ii) Respiratory antibiotic (EMIS)
COTA696	Co-Trimoxazole Tablets 480 mg	(ii) Respiratory antibiotic (EMIS)
COTA697	Co-Trimoxazole Tablets 960 mg	(ii) Respiratory antibiotic (EMIS)
DECA4806	Demeclocycline Hydrochloride Capsules 150 mg	(ii) Respiratory antibiotic (EMIS)
DETA4807	Demeclocycline Hydrochloride Tablets 300 mg	(ii) Respiratory antibiotic (EMIS)
DICA20697EMIS	Distaclor Capsules 500 mg	(ii) Respiratory antibiotic (EMIS)
DICA940	Distaclor Capsules 250 mg	(ii) Respiratory antibiotic (EMIS)
DIM/22445EMIS	Distaclor Mr M/R tablets 375 mg	(ii) Respiratory antibiotic (EMIS)
DIM/27776EMIS	Distaclor Mr M/R tablets 500 mg	(ii) Respiratory antibiotic (EMIS)
DISU941	Distaclor Suspension 125 mg/5 ml	(ii) Respiratory antibiotic (EMIS)
DISU942	Distaclor Suspension 250 mg/5 ml	(ii) Respiratory antibiotic (EMIS)
DOCA17639NEMIS	Doxycycline Hyclate Capsules 100 mg	(ii) Respiratory antibiotic (EMIS)
DOCA32449EMIS	Doxycycline Hyclate Capsules 50 mg	(ii) Respiratory antibiotic (EMIS)
DOCA4868	Doxycycline Capsules 100 mg	(ii) Respiratory antibiotic (EMIS)
DOCA7112	Doxycycline Capsules 50 mg	(ii) Respiratory antibiotic (EMIS)
DODI17640NEMIS	Doxycycline Hyclate Dispersible tablets 100 mg	(ii) Respiratory antibiotic (EMIS)
DODI4869	Doxycycline Dispersible tablets 100 mg	(ii) Respiratory antibiotic (EMIS)
DOM/37467NEMIS	Doxycycline Monohydrate M/R Capsules 40 mg	(ii) Respiratory antibiotic (EMIS)
DOM/37467NEMIS	Doxycycline 40mg modified-release capsules	(ii) Respiratory antibiotic (EMIS)
DOOR74087NEMIS	Doxycycline 50mg/5ml oral suspension	(ii) Respiratory antibiotic (EMIS)
DOOR74087NEMIS	Doxycycline Oral Suspension 50 mg/5 ml	(ii) Respiratory antibiotic (EMIS)
DOSY4870	Doxycycline Syrup 50 mg/5 ml	(ii) Respiratory antibiotic (EMIS)
DOTA983	Doxycycline Tablets 100 mg	(ii) Respiratory antibiotic (EMIS)
e311.	AMOXICILLIN 250mg capsules	(ii) Respiratory antibiotic (Read v2)
e312.	AMOXICILLIN 500mg capsules	(ii) Respiratory antibiotic (Read v2)
e315.	AMOXIL 250mg capsules	(ii) Respiratory antibiotic (Read v2)
e316.	AMOXIL 500mg capsules	(ii) Respiratory antibiotic (Read v2)
e317.	AMOXIL 500mg disp tabs	(ii) Respiratory antibiotic (Read v2)
e318.	AMOXIL 125mg/5mL syrup	(ii) Respiratory antibiotic (Read v2)
e319.	AMOXIL SF 125mg/5mL syrup	(ii) Respiratory antibiotic (Read v2)
e31a.	AMOXIL SF 250mg/5mL syrup	(ii) Respiratory antibiotic (Read v2)
e31b.	AMOXIL 125mg/1.25mL suspension	(ii) Respiratory antibiotic (Read v2)
e31c.	AMOXIL SF 750mg sachets	(ii) Respiratory antibiotic (Read v2)
e31d.	AMOXIL SF 3g sachets	(ii) Respiratory antibiotic (Read v2)
e31h.	AUGMENTIN 375mg tablets	(ii) Respiratory antibiotic (Read v2)
e31i.	AUGMENTIN 375mg disp tabs	(ii) Respiratory antibiotic (Read v2)
e31j.	AUGMENTIN JUNIOR 125/62 in 5mL suspension	(ii) Respiratory antibiotic (Read v2)
e31k.	AUGMENTIN 125/31 in 5mL suspension	(ii) Respiratory antibiotic (Read v2)
e31M.	AMOXIL FIZTAB 125mg chew tabs	(ii) Respiratory antibiotic (Read v2)
e31N.	AMOXIL FIZTAB 250mg chew tabs	(ii) Respiratory antibiotic (Read v2)
e31O.	AMOXIL FIZTAB 500mg chew tabs	(ii) Respiratory antibiotic (Read v2)
e31P.	AUGMENTIN 250/62 in 5mL suspension	(ii) Respiratory antibiotic (Read v2)
e31Q.	CO-AMOXICLAV 125/31mg in 5mL suspension	(ii) Respiratory antibiotic (Read v2)
e31t.	CO-AMOXICLAV 375mg tablets	(ii) Respiratory antibiotic (Read v2)
e31T.	AUGMENTIN 625mg tablets	(ii) Respiratory antibiotic (Read v2)
e31u.	CO-AMOXICLAV 375mg disp tabs	(ii) Respiratory antibiotic (Read v2)
e31U.	CO-AMOXICLAV 625mg tablets	(ii) Respiratory antibiotic (Read v2)
e31v.	CO-AMOXICLAV 125mg/5mL suspension	(ii) Respiratory antibiotic (Read v2)
e31w.	CO-AMOXICLAV 125mg/mL suspension	(ii) Respiratory antibiotic (Read v2)
e31X.	CO-AMOXICLAV 400/57mg in 5mL s/f susp	(ii) Respiratory antibiotic (Read v2)

e31Y.	AUGMENTIN-DUO 400/57 in 5mL s/f susp	(ii) Respiratory antibiotic (Read v2)
e31z.	CO-AMOXICLAV 250/62 in 5mL suspension	(ii) Respiratory antibiotic (Read v2)
e321.	AMPICILLIN 250mg capsules	(ii) Respiratory antibiotic (Read v2)
e322.	AMPICILLIN 500mg capsules	(ii) Respiratory antibiotic (Read v2)
e323.	AMPICILLIN 125mg/5mL mixture	(ii) Respiratory antibiotic (Read v2)
e324.	AMPICILLIN 250mg/5mL mixture	(ii) Respiratory antibiotic (Read v2)
e32G.	AMPICILLIN 125mg/1.25mL suspension	(ii) Respiratory antibiotic (Read v2)
e32h.	PENBRITIN 250mg capsules	(ii) Respiratory antibiotic (Read v2)
e32H.	AMPICILLIN 125mg/5mL s/f susp	(ii) Respiratory antibiotic (Read v2)
e32i.	PENBRITIN 500mg capsules	(ii) Respiratory antibiotic (Read v2)
e32j.	PENBRITIN 125mg tablets	(ii) Respiratory antibiotic (Read v2)
e32J.	AMPICILLIN 250mg/5mL s/f susp	(ii) Respiratory antibiotic (Read v2)
e32k.	PENBRITIN 125mg/5mL syrup	(ii) Respiratory antibiotic (Read v2)
e32l.	PENBRITIN 250mg/5mL syrup	(ii) Respiratory antibiotic (Read v2)
e32m.	PENBRITIN 100mg/mL suspension	(ii) Respiratory antibiotic (Read v2)
e32z.	AMPICILLIN 125mg tablets	(ii) Respiratory antibiotic (Read v2)
e3z1.	AMORAM 250mg capsules	(ii) Respiratory antibiotic (Read v2)
e3z2.	AMORAM 500mg capsules	(ii) Respiratory antibiotic (Read v2)
e3z3.	AMORAM 125mg/5mL suspension	(ii) Respiratory antibiotic (Read v2)
e3z4.	AMORAM 250mg/5mL suspension	(ii) Respiratory antibiotic (Read v2)
e3z7.	GALENAMOX 250mg capsules	(ii) Respiratory antibiotic (Read v2)
e3z8.	GALENAMOX 500mg capsules	(ii) Respiratory antibiotic (Read v2)
e3z9.	GALENAMOX 125mg/5mL suspension	(ii) Respiratory antibiotic (Read v2)
e3za.	GALENAMOX 250mg/5mL suspension	(ii) Respiratory antibiotic (Read v2)
e3zA.	GALENAMOX TP 250mg capsules	(ii) Respiratory antibiotic (Read v2)
e3zb.	GALENAMOX 125mg/5mL s/f susp	(ii) Respiratory antibiotic (Read v2)
e3zB.	GALENAMOX TP 500mg capsules	(ii) Respiratory antibiotic (Read v2)
e3zc.	GALENAMOX 250mg/5mL s/f susp	(ii) Respiratory antibiotic (Read v2)
e3zE.	AMOXICILLIN 125mg/sachet s/f pdr	(ii) Respiratory antibiotic (Read v2)
e3zf.	RIMOXALLIN 125mg/5mL syrup	(ii) Respiratory antibiotic (Read v2)
e3zF.	AMOXIDENT 250mg capsules	(ii) Respiratory antibiotic (Read v2)
e3zg.	RIMOXALLIN 250mg capsules	(ii) Respiratory antibiotic (Read v2)
e3zG.	AMOXIDENT 500mg capsules	(ii) Respiratory antibiotic (Read v2)
e3zh.	RIMOXALLIN 500mg capsules	(ii) Respiratory antibiotic (Read v2)
e3zj.	RIMOXALLIN 250mg/5mL syrup	(ii) Respiratory antibiotic (Read v2)
e3zk.	AMOXICILLIN 125mg/5mL s/f susp	(ii) Respiratory antibiotic (Read v2)
e3zm.	AMOXICILLIN 125mg/5mL syrup	(ii) Respiratory antibiotic (Read v2)
e3zn.	AMOXICILLIN 250mg/5mL syrup	(ii) Respiratory antibiotic (Read v2)
e3zo.	AMOXICILLIN 125mg/1.25mL suspension	(ii) Respiratory antibiotic (Read v2)
e3zu.	AMOXICILLIN 250mg/5mL s/f susp	(ii) Respiratory antibiotic (Read v2)
e611.	DISTACLOR 250mg capsules	(ii) Respiratory antibiotic (Read v2)
e612.	DISTACLOR 125mg/5mL suspension	(ii) Respiratory antibiotic (Read v2)
e613.	DISTACLOR 250mg/5mL suspension	(ii) Respiratory antibiotic (Read v2)
e614.	CEFACLOR 250mg capsules	(ii) Respiratory antibiotic (Read v2)
e615.	CEFACLOR 125mg/5mL suspension	(ii) Respiratory antibiotic (Read v2)
e616.	CEFACLOR 250mg/5mL suspension	(ii) Respiratory antibiotic (Read v2)
e617.	DISTACLOR 500mg capsules	(ii) Respiratory antibiotic (Read v2)
e618.	CEFACLOR 500mg capsules	(ii) Respiratory antibiotic (Read v2)
e619.	DISTACLOR MR 375mg m/r tabs	(ii) Respiratory antibiotic (Read v2)
e61a.	CEFACLOR 375mg m/r tabs	(ii) Respiratory antibiotic (Read v2)
e61b.	DISTACLOR MR 500mg m/r tabs	(ii) Respiratory antibiotic (Read v2)
e61c.	CEFACLOR 500mg m/r tabs	(ii) Respiratory antibiotic (Read v2)
e61C.	CEFACLOR 125mg/5mL s/f susp	(ii) Respiratory antibiotic (Read v2)
e61D.	CEFACLOR 250mg/5mL s/f susp	(ii) Respiratory antibiotic (Read v2)
e625.	CEFADROXIL 125mg/5mL suspension	(ii) Respiratory antibiotic (Read v2)
e626.	CEFADROXIL 250mg/5mL suspension	(ii) Respiratory antibiotic (Read v2)
e627.	CEFADROXIL 500mg capsules	(ii) Respiratory antibiotic (Read v2)
e62w.	CEFADROXIL 500mg capsules	(ii) Respiratory antibiotic (Read v2)

e62x.	CEFADROXIL 500mg capsules	(ii) Respiratory antibiotic (Read v2)
e62z.	CEFADROXIL 500mg/5mL suspension	(ii) Respiratory antibiotic (Read v2)
e684.	ZINNAT 125mg tablets	(ii) Respiratory antibiotic (Read v2)
e685.	ZINNAT 250mg tablets	(ii) Respiratory antibiotic (Read v2)
e686.	CEFUROXIME 125mg tablets	(ii) Respiratory antibiotic (Read v2)
e687.	CEFUROXIME 250mg tablets	(ii) Respiratory antibiotic (Read v2)
e689.	ZINNAT 125mg/5mL suspension	(ii) Respiratory antibiotic (Read v2)
e68a.	CEFUROXIME 125mg/5mL suspension	(ii) Respiratory antibiotic (Read v2)
e68b.	ZINNAT 125mg/sachet suspension	(ii) Respiratory antibiotic (Read v2)
e68c.	CEFUROXIME 125mg/sach for susp	(ii) Respiratory antibiotic (Read v2)
e691.	CEFALEXIN 250mg capsules	(ii) Respiratory antibiotic (Read v2)
e692.	CEFALEXIN 500mg capsules	(ii) Respiratory antibiotic (Read v2)
e693.	CEFALEXIN 250mg tablets	(ii) Respiratory antibiotic (Read v2)
e694.	CEFALEXIN 500mg tablets	(ii) Respiratory antibiotic (Read v2)
e695.	CEFALEXIN 125mg/5mL mixture	(ii) Respiratory antibiotic (Read v2)
e696.	CEFALEXIN 250mg/5mL mixture	(ii) Respiratory antibiotic (Read v2)
e697.	CEFALEXIN 500mg/5mL syrup	(ii) Respiratory antibiotic (Read v2)
e698.	CEPOREX 250mg capsules	(ii) Respiratory antibiotic (Read v2)
e699.	CEPOREX 500mg capsules	(ii) Respiratory antibiotic (Read v2)
e69a.	CEPOREX 250mg tablets	(ii) Respiratory antibiotic (Read v2)
e69b.	CEPOREX 500mg tablets	(ii) Respiratory antibiotic (Read v2)
e69c.	CEPOREX 125mg/1.25mL drops	(ii) Respiratory antibiotic (Read v2)
e69d.	CEPOREX 125mg/5mL suspension	(ii) Respiratory antibiotic (Read v2)
e69e.	CEPOREX 250mg/5mL suspension	(ii) Respiratory antibiotic (Read v2)
e69f.	CEPOREX 125mg/5mL syrup	(ii) Respiratory antibiotic (Read v2)
e69g.	CEPOREX 250mg/5mL syrup	(ii) Respiratory antibiotic (Read v2)
e69h.	CEPOREX 500mg/5mL syrup	(ii) Respiratory antibiotic (Read v2)
e69i.	KEFLEX 250mg capsules	(ii) Respiratory antibiotic (Read v2)
e69j.	KEFLEX 500mg capsules	(ii) Respiratory antibiotic (Read v2)
e69k.	KEFLEX 250mg tablets	(ii) Respiratory antibiotic (Read v2)
e69l.	KEFLEX 500mg tablets	(ii) Respiratory antibiotic (Read v2)
e69m.	KEFLEX 125mg/5mL suspension	(ii) Respiratory antibiotic (Read v2)
e69n.	KEFLEX 250mg/5mL suspension	(ii) Respiratory antibiotic (Read v2)
e69o.	KEFLEX-C 125mg chew tabs	(ii) Respiratory antibiotic (Read v2)
e69p.	KEFLEX-C 250mg chew tabs	(ii) Respiratory antibiotic (Read v2)
e69q.	CEPOREX 1g tablets	(ii) Respiratory antibiotic (Read v2)
e69v.	CEFALEXIN 125mg/5mL syrup	(ii) Respiratory antibiotic (Read v2)
e69w.	CEFALEXIN 250mg/5mL syrup	(ii) Respiratory antibiotic (Read v2)
e6dv.	CEFRADINE 250mg capsules	(ii) Respiratory antibiotic (Read v2)
e6dw.	CEFRADINE 500mg capsules	(ii) Respiratory antibiotic (Read v2)
e6dx.	CEFRADINE 250mg/5mL syrup	(ii) Respiratory antibiotic (Read v2)
e6h1.	CEFIXIME 200mg tablets	(ii) Respiratory antibiotic (Read v2)
e6h2.	CEFIXIME 100mg/5mL suspension	(ii) Respiratory antibiotic (Read v2)
e6h3.	SUPRAX 200mg tablets	(ii) Respiratory antibiotic (Read v2)
e6h4.	SUPRAX 100mg/5mL suspension	(ii) Respiratory antibiotic (Read v2)
e6h5.	SUPRAX 100mg/5mL suspension	(ii) Respiratory antibiotic (Read v2)
e6h6.	SUPRAX 100mg/5mL suspension	(ii) Respiratory antibiotic (Read v2)
e6h7.	SUPRAX 100mg/5mL suspension	(ii) Respiratory antibiotic (Read v2)
e6k0.	CEFPODOXIME 100mg tablets	(ii) Respiratory antibiotic (Read v2)
e6k2.	CEFPODOXIME 40mg/5mL suspension	(ii) Respiratory antibiotic (Read v2)
e711.	TETRACYCLINE 250mg capsules	(ii) Respiratory antibiotic (Read v2)
e712.	TETRACYCLINE 250mg tablets	(ii) Respiratory antibiotic (Read v2)
e713.	TETRACYCLINE 125mg/5mL mixture	(ii) Respiratory antibiotic (Read v2)
e71p.	TETRACYCLINE 250mg m/r caps	(ii) Respiratory antibiotic (Read v2)
e71z.	COMPOUND TETRACYCLINE 300mg tablets	(ii) Respiratory antibiotic (Read v2)
e72y.	CHLORTETRACYCLINE 250mg capsules	(ii) Respiratory antibiotic (Read v2)
e74y.	DEMECLOCYCLINE HCL 150mg capsules	(ii) Respiratory antibiotic (Read v2)
e74z.	DEMECLOCYCLINE HCL 300mg tablets	(ii) Respiratory antibiotic (Read v2)

e754.	VIBRAMYCIN 50mg capsules	(ii) Respiratory antibiotic (Read v2)
e755.	VIBRAMYCIN 100mg capsules	(ii) Respiratory antibiotic (Read v2)
e756.	VIBRAMYCIN 50mg/5mL syrup	(ii) Respiratory antibiotic (Read v2)
e757.	VIBRAMYCIN-D 100mg disp tabs	(ii) Respiratory antibiotic (Read v2)
e758.	DOXYCYCLINE 100mg capsules	(ii) Respiratory antibiotic (Read v2)
e75s.	DOXYCYCLINE 40mg m/r caps	(ii) Respiratory antibiotic (Read v2)
e75v.	DOXYCYCLINE 100mg tablets	(ii) Respiratory antibiotic (Read v2)
e75w.	DOXYCYCLINE 100mg capsules	(ii) Respiratory antibiotic (Read v2)
e75x.	DOXYCYCLINE 50mg capsules	(ii) Respiratory antibiotic (Read v2)
e75y.	DOXYCYCLINE 50mg/5mL syrup	(ii) Respiratory antibiotic (Read v2)
e75z.	DOXYCYCLINE 100mg disp tabs	(ii) Respiratory antibiotic (Read v2)
e761.	TETRALYSAL 204mg capsules	(ii) Respiratory antibiotic (Read v2)
e762.	TETRALYSAL-300 408mg capsules	(ii) Respiratory antibiotic (Read v2)
e76y.	LYMECYCLINE 204mg capsules	(ii) Respiratory antibiotic (Read v2)
e76z.	LYMECYCLINE 408mg capsules	(ii) Respiratory antibiotic (Read v2)
e781.	OXYTETRACYCLINE 250mg capsules	(ii) Respiratory antibiotic (Read v2)
e782.	OXYTETRACYCLINE 250mg tablets	(ii) Respiratory antibiotic (Read v2)
e783.	OXYTETRACYCLINE 125mg/5mL mixture	(ii) Respiratory antibiotic (Read v2)
e789.	OXYMYCIN 250mg tablets	(ii) Respiratory antibiotic (Read v2)
e78y.	OXYTETRACYCLINE 125mg/5mL syrup	(ii) Respiratory antibiotic (Read v2)
e79z.	TETRACYCLINE+NYSTATIN 250mg/250ku tablets	(ii) Respiratory antibiotic (Read v2)
e911.	ERYTHROMYCIN 250mg e/c tabs	(ii) Respiratory antibiotic (Read v2)
e912.	ERYTHROMYCIN 500mg tablets	(ii) Respiratory antibiotic (Read v2)
e913.	ERYTHROMYCIN STEARATE 250mg tablets	(ii) Respiratory antibiotic (Read v2)
e914.	ERYTHROMYCIN STEARATE 500mg tablets	(ii) Respiratory antibiotic (Read v2)
e91a.	ERYMAX 250mg e/c grans	(ii) Respiratory antibiotic (Read v2)
e91A.	ERYTHROPED FORTE grans	(ii) Respiratory antibiotic (Read v2)
e91b.	ERYTHROCIN 250mg tablets	(ii) Respiratory antibiotic (Read v2)
e91B.	ERYTHROPED P.I. grans	(ii) Respiratory antibiotic (Read v2)
e91c.	ERYTHROCIN 500mg tablets	(ii) Respiratory antibiotic (Read v2)
e91C.	ERYTHROPED P.I. 125mg/5mL s/f susp	(ii) Respiratory antibiotic (Read v2)
e91D.	ERYTHROPED 250mg/5ml s/f susp	(ii) Respiratory antibiotic (Read v2)
e91E.	ERYTHROMYCIN 125mg/5mL s/f susp	(ii) Respiratory antibiotic (Read v2)
e91F.	ERYTHROMYCIN 250mg/5mL s/f susp	(ii) Respiratory antibiotic (Read v2)
e91I.	KERYMAX 250mg e/c grans	(ii) Respiratory antibiotic (Read v2)
e91j.	ERYTHROPED P.I. 125mg/5mL suspension	(ii) Respiratory antibiotic (Read v2)
e91k.	ERYTHROPED 250mg/5mL suspension	(ii) Respiratory antibiotic (Read v2)
e91l.	ERYTHROPED 250mg/sachet s/f grans	(ii) Respiratory antibiotic (Read v2)
e91L.	ERYTHROMYCIN 250mg capsules	(ii) Respiratory antibiotic (Read v2)
e91m.	ERYTHROPED FORTE 500mg/5mL suspension	(ii) Respiratory antibiotic (Read v2)
e91M.	ERYTHROMYCIN 125mg/sachet grans	(ii) Respiratory antibiotic (Read v2)
e91n.	ERYTHROPED A 500mg tablets	(ii) Respiratory antibiotic (Read v2)
e91N.	ERYTHROMYCIN 250mg/sachet grans	(ii) Respiratory antibiotic (Read v2)
e91P.	ERYTHROMYCIN 500mg/sachet grans	(ii) Respiratory antibiotic (Read v2)
e91Q.	ERYTHROMYCIN 1g/sachet grans	(ii) Respiratory antibiotic (Read v2)
e91R.	ERYTHROMYCIN 500mg/5mL s/f susp	(ii) Respiratory antibiotic (Read v2)
e91S.	TILORYTH 250mg e/c grans	(ii) Respiratory antibiotic (Read v2)
e91u.	ERYTHROMYCIN 125mg/5mL suspension	(ii) Respiratory antibiotic (Read v2)
e91v.	ERYTHROMYCIN 250mg/5mL suspension	(ii) Respiratory antibiotic (Read v2)
e91w.	ERYTHROMYCIN 500mg/5mL suspension	(ii) Respiratory antibiotic (Read v2)
e91x.	ERYTHROPED A 1g/sachet grans	(ii) Respiratory antibiotic (Read v2)
e91X.	ERYTHROMYCIN 250mg e/c grans	(ii) Respiratory antibiotic (Read v2)
e91y.	ERYMAX SPRINKLE 125mg capsules	(ii) Respiratory antibiotic (Read v2)
e91Y.	ERYTHROPED FORTE SF 500mg/5mL s/f susp	(ii) Respiratory antibiotic (Read v2)
e91z.	ERYTHROPED 250mg/sachet grans	(ii) Respiratory antibiotic (Read v2)
e921.	CLARITHROMYCIN 250mg tablets	(ii) Respiratory antibiotic (Read v2)
e922.	KLARICID 250mg tablets	(ii) Respiratory antibiotic (Read v2)
e923.	CLARITHROMYCIN 125mg/5mL suspension	(ii) Respiratory antibiotic (Read v2)

e924.	KLARICID 125mg/5mL suspension	(ii) Respiratory antibiotic (Read v2)
e927.	CLARITHROMYCIN 500mg tablets	(ii) Respiratory antibiotic (Read v2)
e928.	KLARICID 500mg tablets	(ii) Respiratory antibiotic (Read v2)
e929.	CLARITHROMYCIN 500mg m/r tabs	(ii) Respiratory antibiotic (Read v2)
e92A.	KLARICID XL 500mg m/r tabs	(ii) Respiratory antibiotic (Read v2)
e92B.	CLARITHROMYCIN 250mg/sachet grans	(ii) Respiratory antibiotic (Read v2)
e92C.	KLARICID 250mg/sachet grans	(ii) Respiratory antibiotic (Read v2)
e92D.	CLARITHROMYCIN 250mg/5mL suspension	(ii) Respiratory antibiotic (Read v2)
e92E.	KLARICID 250mg/5mL suspension	(ii) Respiratory antibiotic (Read v2)
e92F.	CLARITHROMYCIN 125mg grans	(ii) Respiratory antibiotic (Read v2)
e92H.	CLARITHROMYCIN 187.5mg grans	(ii) Respiratory antibiotic (Read v2)
e92J.	CLARITHROMYCIN 250mg grans	(ii) Respiratory antibiotic (Read v2)
e931.	AZITHROMYCIN 250mg capsules	(ii) Respiratory antibiotic (Read v2)
e932.	AZITHROMYCIN 40mg/mL suspension	(ii) Respiratory antibiotic (Read v2)
e933.	ZITHROMAX 250mg capsules	(ii) Respiratory antibiotic (Read v2)
e934.	ZITHROMAX 40mg/mL suspension	(ii) Respiratory antibiotic (Read v2)
e935.	ZITHROMAX 40mg/mL suspension	(ii) Respiratory antibiotic (Read v2)
e936.	ZITHROMAX 40mg/mL suspension	(ii) Respiratory antibiotic (Read v2)
e937.	AZITHROMYCIN 500mg tablets	(ii) Respiratory antibiotic (Read v2)
e938.	ZITHROMAX 500mg tablets	(ii) Respiratory antibiotic (Read v2)
e939.	CLAMELLE AZITHROMYCIN 500mg tablets	(ii) Respiratory antibiotic (Read v2)
ec11.	CO-TRIMOXAZOLE 480mg tablets	(ii) Respiratory antibiotic (Read v2)
ec12.	CO-TRIMOXAZOLE 480mg disp tabs	(ii) Respiratory antibiotic (Read v2)
ec13.	CO-TRIMOXAZOLE 960mg tablets	(ii) Respiratory antibiotic (Read v2)
ec14.	CO-TRIMOXAZOLE 960mg disp tabs	(ii) Respiratory antibiotic (Read v2)
ec15.	CO-TRIMOXAZOLE 120mg tablets	(ii) Respiratory antibiotic (Read v2)
ec16.	CO-TRIMOXAZOLE 480mg/5mL mixture	(ii) Respiratory antibiotic (Read v2)
ec17.	CO-TRIMOXAZOLE 240mg/5mL mixture	(ii) Respiratory antibiotic (Read v2)
ec1A.	CO-TRIMOXAZOLE 240mg/5mL s/f susp	(ii) Respiratory antibiotic (Read v2)
ec2l.	SEPTRIN 480mg tablets	(ii) Respiratory antibiotic (Read v2)
ec2m.	SEPTRIN 480mg disp tabs	(ii) Respiratory antibiotic (Read v2)
ec2n.	SEPTRIN FORTE 960mg tablets	(ii) Respiratory antibiotic (Read v2)
ec2o.	SEPTRIN PAEDIATRIC 120mg disp tabs	(ii) Respiratory antibiotic (Read v2)
ec2p.	SEPTRIN 480mg/5mL adult susp	(ii) Respiratory antibiotic (Read v2)
ec2q.	SEPTRIN 240mg/5mL suspension	(ii) Respiratory antibiotic (Read v2)
eg61.	CIPROXIN 250mg tablets	(ii) Respiratory antibiotic (Read v2)
eg64.	CIPROXIN 500mg tablets	(ii) Respiratory antibiotic (Read v2)
eg65.	CIPROXIN 750mg tablets	(ii) Respiratory antibiotic (Read v2)
eg67.	CIPROFLOXACIN 100mg tablets	(ii) Respiratory antibiotic (Read v2)
eg68.	CIPROXIN 100mg tablets	(ii) Respiratory antibiotic (Read v2)
eg69.	CIPROFLOXACIN 5g/100mL suspension	(ii) Respiratory antibiotic (Read v2)
eg6A.	CIPROXIN 5g/100mL suspension	(ii) Respiratory antibiotic (Read v2)
eg6v.	CIPROFLOXACIN 750mg tablets	(ii) Respiratory antibiotic (Read v2)
eg6w.	CIPROFLOXACIN 500mg tablets	(ii) Respiratory antibiotic (Read v2)
eg6x.	CIPROFLOXACIN 250mg tablets	(ii) Respiratory antibiotic (Read v2)
eg81.	OFLOXACIN 200mg tablets	(ii) Respiratory antibiotic (Read v2)
eg82.	TARIVID 200mg tablets	(ii) Respiratory antibiotic (Read v2)
eg87.	TARIVID 400mg tablets	(ii) Respiratory antibiotic (Read v2)
eg88.	OFLOXACIN 400mg tablets	(ii) Respiratory antibiotic (Read v2)
egC1.	LEVOFLOXACIN 250mg tablets	(ii) Respiratory antibiotic (Read v2)
egC2.	LEVOFLOXACIN 500mg tablets	(ii) Respiratory antibiotic (Read v2)
egC4.	TAVANIC 250mg tablets	(ii) Respiratory antibiotic (Read v2)
egC5.	TAVANIC 500mg tablets	(ii) Respiratory antibiotic (Read v2)
egD1.	MOXIFLOXACIN 400mg tablets	(ii) Respiratory antibiotic (Read v2)
egD2.	AVELOX 400mg tablets	(ii) Respiratory antibiotic (Read v2)
ERCA3531	Erymax Capsules (Gastro-Resistant) 250 mg	(ii) Respiratory antibiotic (EMIS)
ERCA4890	Erythromycin Capsules (Gastro-Resistant) 250 mg	(ii) Respiratory antibiotic (EMIS)
ERE/2023NEMIS	Erythromycin E/c tablets 250 mg	(ii) Respiratory antibiotic (EMIS)

ERGR10346BRIDL	Erythromycin Ethyl Succinate Granules 1 gram/sachet	(ii) Respiratory antibiotic (EMIS)
ERGR25258EMIS	Erythromycin Ethyl Succinate Granules 125 mg/sachet	(ii) Respiratory antibiotic (EMIS)
ERGR25259EMIS	Erythromycin Ethyl Succinate Granules 250 mg/sachet	(ii) Respiratory antibiotic (EMIS)
ERGR25260EMIS	Erythromycin Ethyl Succinate Granules 500 mg/sachet	(ii) Respiratory antibiotic (EMIS)
ERGR9480HILLI	Erythroped Granules forte 500 mg/sachet	(ii) Respiratory antibiotic (EMIS)
ERMI25261EMIS	Erythromycin Ethyl Succinate Mixture 125 mg/5 ml	(ii) Respiratory antibiotic (EMIS)
ERMI25262EMIS	Erythromycin Ethyl Succinate Mixture 250 mg/5 ml	(ii) Respiratory antibiotic (EMIS)
ERMI25263EMIS	Erythromycin Ethyl Succinate Mixture 500 mg/5 ml	(ii) Respiratory antibiotic (EMIS)
ERP.19608EMIS	Erythroped P.i. sachets 125 mg/sachet	(ii) Respiratory antibiotic (EMIS)
ERP.19609EMIS	Erythroped P.i. sugar-free suspension 125 mg/5 ml	(ii) Respiratory antibiotic (EMIS)
ERPA1102	Erythromycin Paediatric suspension 125 mg/5 ml	(ii) Respiratory antibiotic (EMIS)
ERPA1106	Erythroped P.i. suspension 125 mg/5 ml	(ii) Respiratory antibiotic (EMIS)
ERSA19604EMIS	Erythroped Sachets 250 mg/sachet	(ii) Respiratory antibiotic (EMIS)
ERSA8879BRIDL	Erythroped A Sachets 1 gram/sachet	(ii) Respiratory antibiotic (EMIS)
ERSP8479EGTON	Erymax Sprinkle capsules 125 mg	(ii) Respiratory antibiotic (EMIS)
ERST3539	Erythromycin Tablets 250 mg	(ii) Respiratory antibiotic (EMIS)
ERST3540	Erythromycin Stearate tablets 500 mg	(ii) Respiratory antibiotic (EMIS)
ERST4896	Erythromycin (As Stearate) Stearate tablets 250 mg	(ii) Respiratory antibiotic (EMIS)
ERSU1107	Erythroped Suspension 250 mg/5 ml	(ii) Respiratory antibiotic (EMIS)
ERSU1108	Erythroped Suspension forte Sugar Free 500 mg/5 ml	(ii) Respiratory antibiotic (EMIS)
ERSU19605EMIS	Erythroped Sugar-free suspension 250 mg/5 ml	(ii) Respiratory antibiotic (EMIS)
ERSU25264EMIS	Erythromycin Ethyl Succinate Sugar-free suspension 125 mg/5 ml	(ii) Respiratory antibiotic (EMIS)
ERSU25265EMIS	Erythromycin Ethyl Succinate Sugar-free suspension 250 mg/5 ml	(ii) Respiratory antibiotic (EMIS)
ERSU25266EMIS	Erythromycin Ethyl Succinate Suspension 125 mg/5 ml	(ii) Respiratory antibiotic (EMIS)
ERSU25267EMIS	Erythromycin Ethyl Succinate Suspension 250 mg/5 ml	(ii) Respiratory antibiotic (EMIS)
ERSU25268EMIS	Erythromycin Ethyl Succinate Suspension 500 mg/5 ml	(ii) Respiratory antibiotic (EMIS)
ERSU28989EMIS	Erythromycin Ethyl Succinate Sugar-free suspension 500 mg/5 ml	(ii) Respiratory antibiotic (EMIS)
ERSU3542	Erythroped Sugar free granules 250 mg/sachet	(ii) Respiratory antibiotic (EMIS)
ERSU4893	Erythromycin Sugar free granules 250 mg/sachet	(ii) Respiratory antibiotic (EMIS)
ERTA10453BRIDL	Erythromycin Ethyl Succinate Tablets 500 mg	(ii) Respiratory antibiotic (EMIS)
ERTA3533	Erythrocin Tablets 250 mg	(ii) Respiratory antibiotic (EMIS)
ERTA3534	Erythrocin Tablets 500 mg	(ii) Respiratory antibiotic (EMIS)
ERTA3541	Erythromycin Tablets 500 mg	(ii) Respiratory antibiotic (EMIS)
ERTA3543	Erythroped A Tablets 500 mg	(ii) Respiratory antibiotic (EMIS)
GACA10300BRIDL	Galenamox Capsules 250 mg	(ii) Respiratory antibiotic (EMIS)
GACA10302BRIDL	Galenamox Capsules 500 mg	(ii) Respiratory antibiotic (EMIS)
GASU10304BRIDL	Galenamox Suspension 250 mg/5 ml	(ii) Respiratory antibiotic (EMIS)
GASU10306BRIDL	Galenamox Suspension 125 mg/5 ml	(ii) Respiratory antibiotic (EMIS)
KECA1547	Keflex Capsules 250 mg	(ii) Respiratory antibiotic (EMIS)
KECA1548	Keflex Capsules 500 mg	(ii) Respiratory antibiotic (EMIS)
KECH1553	Keflex-C Chewable tablets 125 mg	(ii) Respiratory antibiotic (EMIS)
KECH1554	Keflex-C Chewable tablets 250 mg	(ii) Respiratory antibiotic (EMIS)
KESU1549	Keflex Suspension 125 mg/5 ml	(ii) Respiratory antibiotic (EMIS)
KESU1550	Keflex Suspension 250 mg/5 ml	(ii) Respiratory antibiotic (EMIS)
KETA1551	Keflex Tablets 250 mg	(ii) Respiratory antibiotic (EMIS)
KETA1552	Keflex Tablets 500 mg	(ii) Respiratory antibiotic (EMIS)
KLM/30083EMIS	Klaricid Xl M/R tablets 500 mg	(ii) Respiratory antibiotic (EMIS)
KLPA22475EMIS	Klaricid Paediatric suspension 125 mg/5 ml	(ii) Respiratory antibiotic (EMIS)
KLPA771NEMIS	Klaricid Paediatric suspension 250 mg/5 ml	(ii) Respiratory antibiotic (EMIS)
KLSA31716EMIS	Klaricid Sachets 250 mg/sachet	(ii) Respiratory antibiotic (EMIS)
KLTA26170EMIS	Klaricid Tablets 500 mg	(ii) Respiratory antibiotic (EMIS)
KLTA9107EMIS	Klaricid Tablets 250 mg	(ii) Respiratory antibiotic (EMIS)
LETA32269EMIS	Levofloxacin Tablets 250 mg	(ii) Respiratory antibiotic (EMIS)
LETA32270EMIS	Levofloxacin Tablets 500 mg	(ii) Respiratory antibiotic (EMIS)
LYCA5100	Lymecycline Capsules 204 mg	(ii) Respiratory antibiotic (EMIS)
LYCA5101	Lymecycline Capsules 408 mg	(ii) Respiratory antibiotic (EMIS)
MOTA15042NEMIS	Moxifloxacin Tablets 400 mg	(ii) Respiratory antibiotic (EMIS)
OFTA10706BRIDL	Ofloxacin Tablets 200 mg	(ii) Respiratory antibiotic (EMIS)

OFTA23520EMIS	Ofloxacin Tablets 400 mg	(ii) Respiratory antibiotic (EMIS)
OXCA5242	Oxytetracycline Hydrochloride Capsules 250 mg	(ii) Respiratory antibiotic (EMIS)
OXMI4104	Oxytetracycline Mixture 125 mg/5 ml	(ii) Respiratory antibiotic (EMIS)
OXSY5240	Oxytetracycline Syrup 125 mg/5 ml	(ii) Respiratory antibiotic (EMIS)
OXTA2146	Oxytetracycline Tablets 250 mg	(ii) Respiratory antibiotic (EMIS)
OXTA4102	Oxymycin Tablets 250 mg	(ii) Respiratory antibiotic (EMIS)
OXTA5241	Oxytetracycline Dihydrate Tablets 250 mg	(ii) Respiratory antibiotic (EMIS)
PECA2172	Penbritin Capsules 250 mg	(ii) Respiratory antibiotic (EMIS)
PECA2173	Penbritin Capsules 500 mg	(ii) Respiratory antibiotic (EMIS)
PEPA2176	Penbritin Paediatric suspension 125 mg/1.25 ml	(ii) Respiratory antibiotic (EMIS)
PEPA2177	Penbritin Paediatric tablets 125 mg	(ii) Respiratory antibiotic (EMIS)
PESY2178	Penbritin Syrup 125 mg/5 ml	(ii) Respiratory antibiotic (EMIS)
PESY2179	Penbritin Syrup forte 250 mg/5 ml	(ii) Respiratory antibiotic (EMIS)
SEAD2540	Septrin Adult suspension 480 mg/5 ml	(ii) Respiratory antibiotic (EMIS)
SEDI2541	Septrin Dispersible tablets 480 mg	(ii) Respiratory antibiotic (EMIS)
SEFO2542	Septrin Forte tablets 960 mg	(ii) Respiratory antibiotic (EMIS)
SEPA2545	Septrin Paediatric suspension 240 mg/5 ml	(ii) Respiratory antibiotic (EMIS)
SEPA2546	Septrin Paediatric tablets 120 mg	(ii) Respiratory antibiotic (EMIS)
SETA2547	Septrin Tablets 480 mg	(ii) Respiratory antibiotic (EMIS)
SETA2548	Septrin Forte Tablets 960 mg	(ii) Respiratory antibiotic (EMIS)
SUCA22413NEMIS	Suprax Capsules 400 mg	(ii) Respiratory antibiotic (EMIS)
SUPA10644BRIDL	Suprax Paediatric suspension 100 mg/5 ml	(ii) Respiratory antibiotic (EMIS)
SUTA8925EGTON	Suprax Tablets 200 mg	(ii) Respiratory antibiotic (EMIS)
TATA23518EMIS	Tarivid Tablets 400 mg	(ii) Respiratory antibiotic (EMIS)
TATA32263EMIS	Tavanic Tablets 250 mg	(ii) Respiratory antibiotic (EMIS)
TATA32264EMIS	Tavanic Tablets 500 mg	(ii) Respiratory antibiotic (EMIS)
TATA8954EMIS	Tarivid Tablets 200 mg	(ii) Respiratory antibiotic (EMIS)
TECA4411	Tetralysal 300 Capsules 408 mg	(ii) Respiratory antibiotic (EMIS)
TECA5420	Tetracycline Hydrochloride Capsules 250 mg	(ii) Respiratory antibiotic (EMIS)
TEMI5423	Tetracycline Hydrochloride Mixture 125 mg/5 ml	(ii) Respiratory antibiotic (EMIS)
TES/10779BRIDL	Tetracycline Hydrochloride S/r capsules 250 mg	(ii) Respiratory antibiotic (EMIS)
TESY5425	Tetracycline Hydrochloride Syrup 125 mg/5 ml	(ii) Respiratory antibiotic (EMIS)
TETA5426	Tetracycline Hydrochloride Tablets 250 mg	(ii) Respiratory antibiotic (EMIS)
TICA27975EMIS	Tiloryth Capsules 250 mg	(ii) Respiratory antibiotic (EMIS)
VICA4503	Vibramycin Capsules 100 mg	(ii) Respiratory antibiotic (EMIS)
VICA4504	Vibramycin Capsules 50 mg	(ii) Respiratory antibiotic (EMIS)
VISY4505	Vibramycin Syrup 50 mg/5 ml	(ii) Respiratory antibiotic (EMIS)
VITA3041	Vibramycin-D Dispersible tablets 100 mg	(ii) Respiratory antibiotic (EMIS)
ZICA1003EMIS	Zithromax Capsules 250 mg	(ii) Respiratory antibiotic (EMIS)
ZISA20463EMIS	Zinnat Sachets 125 mg/sachet	(ii) Respiratory antibiotic (EMIS)
ZISU9169EMIS	Zinnat Suspension 125 mg/5 ml	(ii) Respiratory antibiotic (EMIS)
ZISU9377EMIS	Zithromax Suspension 200 mg/5 ml	(ii) Respiratory antibiotic (EMIS)
ZITA10283BRIDL	Zinnat Tablets 250 mg	(ii) Respiratory antibiotic (EMIS)
ZITA14216NEMIS	Zinnat Tablets 500 mg	(ii) Respiratory antibiotic (EMIS)
ZITA34167EMIS	Zithromax Tablets 500 mg	(ii) Respiratory antibiotic (EMIS)
ZITA5627	Zinnat Tablets 125 mg	(ii) Respiratory antibiotic (EMIS)
fe61.	PREDNISOLONE 1mg tablets	(ii) Oral corticosteroid (Read v2)
fe62.	PREDNISOLONE 5mg tablets	(ii) Oral corticosteroid (Read v2)
fe6f.	PREDNESOL 5mg tablets	(ii) Oral corticosteroid (Read v2)
fe6h.	PREDNISOLONE 2.5mg e/c tabs	(ii) Oral corticosteroid (Read v2)
fe6i.	PREDNISOLONE 5mg e/c tabs	(ii) Oral corticosteroid (Read v2)
fe6j.	PREDNISOLONE 5mg sol.tabs	(ii) Oral corticosteroid (Read v2)
fe6k.	PREDNISOLONE 50mg tablets	(ii) Oral corticosteroid (Read v2)
fe6v.	PREDNISOLONE 2.5mg tablets	(ii) Oral corticosteroid (Read v2)
fe6w.	PREDNISOLONE 2.5mg tablets	(ii) Oral corticosteroid (Read v2)
fe6z.	PREDNISOLONE 25mg tablets	(ii) Oral corticosteroid (Read v2)
PREN8411EGTON	Prednisolone E/c tablets 2.5 mg	(ii) Oral corticosteroid (EMIS)
PREN8412EGTON	Prednisolone E/c tablets 5 mg	(ii) Oral corticosteroid (EMIS)

PRSO10225BRIDL	Prednisolone Soluble tablets 5 mg	(ii) Oral corticosteroid (EMIS)
PRTA18432EMIS	Prednisolone Tablets 25 mg	(ii) Oral corticosteroid (EMIS)
PRTA2326	Prednisolone Tablets 1 mg	(ii) Oral corticosteroid (EMIS)
PRTA2327	Prednisolone Tablets 2.5 mg	(ii) Oral corticosteroid (EMIS)
PRTA2328	Prednisolone Tablets 5 mg	(ii) Oral corticosteroid (EMIS)
PRTA4200	Prednesol Soluble tablets 5 mg	(ii) Oral corticosteroid (EMIS)
J40X	Bronchitis not specified as acute or chronic	(iii) COPD-related inpatient admission (ICD10)
J42X	Unspecified chronic bronchitis	(iii) COPD-related inpatient admission (ICD10)
J432	Centrilobular emphysema	(iii) COPD-related inpatient admission (ICD10)
J439	Emphysema unspecified	(iii) COPD-related inpatient admission (ICD10)
J440	Chronic obstructive pulmonary disease with acute lower respiratory infection	(iii) COPD-related inpatient admission (ICD10)
J441	Chronic obstructive pulmonary disease with acute exacerbation unspecified	(iii) COPD-related inpatient admission (ICD10)
J448	Other specified chronic obstructive pulmonary disease	(iii) COPD-related inpatient admission (ICD10)
J449	Chronic obstructive pulmonary disease unspecified	(iii) COPD-related inpatient admission (ICD10)

Compiling the DOSE index: Dyspnoea component

The Dyspnoea component was identified using Read codes in the patient's primary care record that were indicative of the Medical Research Council dyspnoea scale assessment.

Code	Description
173H.	MRC Breathless Scale: grade 1
173I.	MRC Breathless Scale: grade 2
173J.	MRC Breathless Scale: grade 3
173K.	MRC Breathless Scale: grade 4
173L.	MRC Breathless Scale: grade 5

Compiling the DOSE index: Obstruction component

The Obstruction component was primarily identified using Read codes in the patient's primary care record that were indicative of the percent predicted forced expiratory volume in one second (FEV_1%). FEV_1% values were taken as being out of valid clinical range (OOR) and excluded where less than 10% or greater than 140%.

Code	Description
339S.	Percent predicted FEV1
339S0	Percent pred FEV1 bronchodiln

To minimise data loss we calculated FEV_1% values from valid forced expiratory volume in one second values (FEV_1), only where they were identified on dates where FEV_1% records were absent or OOR. The equations used to convert FEV_1 to FEV_1% were [9]:

- **If patient male**; $FEV_1\% = \left\{\frac{FEV_1}{[(4.30 \times Patient\ height) - (0.029 \times Patient\ age) - 2.49]]}\right\} \times 100$

- **If patient female**; $FEV_1\% = \left\{\frac{FEV_1}{[(3.95 \times Patient\ height) - (0.025 \times Patient\ age) - 2.60]]}\right\} \times 100$

FEV1 assessment values were themselves taken as being OOR and excluded where less than 0.2 litres or greater than 7.0 litres. Patient height values were standardised into metres, and taken as being OOR and excluded where less than 1.219m (4'0'') or greater than 2.134m (7'0'').

Code	Description
339a.	FEV1 before bronchodilation
339b.	FEV1 after bronchodilation
339e.	FEV1 pre steroids
339f.	FEV1 post steroids
339O.	Forced expired volume in 1 sec
229..	O/E - height
2291.	O/E-height > 20% below average
2292.	O/E - height 10-20% < average
2293.	O/E -height within 10% average
2294.	O/E-height 10-20% over average
2295.	O/E -height > 20% over average
229Z.	O/E - height NOS
22Z..	Height and Weight

Compiling the DOSE index: Smoking component

The Smoking component was identified using Read codes in the patient's primary care record indicating their smoking status at the time of recording, aggregated to create three broad categories: current smoker, ex-smoker and never smoked. As only current smoking takes a non-zero score for calculation of the DOSE index, both ex- smoker and never smoked status were taken as non-smoker. For the same reason we determined prior to data analysis that if conflicting codes for both current and any other smoking status were recorded on the same date, the current smoker status would be selected.

Our definition of current smoking included those smoking cessation advice codes (including codes for medication used in smoking cessation support) which implied that the patient was an active smoker at the time of recording. We especially excluded those few non-specific smoking advice codes (that could equally be employed in counselling never smokers as smokers) from any smoking category definition. Our ex-smoker code list includes only codes that indicate that a patient has stopped smoking at the time of the record. We believe that we reduced the likelihood of misclassifying patients in the process of giving up smoking as ex-smokers by including all smoking-cessation support codes in the list of codes that define current smokers.

Code	Description	Defined as
137..	Tobacco consumption	Current Smoker
1372.	Trivial smoker - < 1 cig/day	Current Smoker
1373.	Light smoker - 1-9 cigs/day	Current Smoker
1374.	Moderate smoker - 10-19 cigs/d	Current Smoker
1375.	Heavy smoker - 20-39 cigs/day	Current Smoker
1376.	Very heavy smoker - 40+cigs/d	Current Smoker
137b.	Ready to stop smoking	Current Smoker
137c.	Thinking about stop smoking	Current Smoker
137C.	Keeps trying to stop smoking	Current Smoker
137d.	Not interested in stop smoking	Current Smoker
137D.	Admitted tobacco cons untrue ?	Current Smoker
137e.	Smoking restarted	Current Smoker
137E.	Tobacco consumption unknown	Current Smoker
137f.	Reason for restarting smoking	Current Smoker
137G.	Trying to give up smoking	Current Smoker
137h.	Min from wake to 1st tobac con	Current Smoker
137H.	Pipe smoker	Current Smoker
137J.	Cigar smoker	Current Smoker
137m.	Failed attempt to stop smoking	Current Smoker
137M.	Rolls own cigarettes	Current Smoker

137n.	Total time smoked	Current Smoker
137P.	Cigarette smoker	Current Smoker
137Q.	Smoking started	Current Smoker
137R.	Current smoker	Current Smoker
137V.	Smoking reduced	Current Smoker
13p0.	Negotiatd date cessatn smoking	Current Smoker
13p5.	Smoking cessn progm start date	Current Smoker
13p50	Practice smok cess pr strt dat	Current Smoker
67H6.	Brf intervention smoking cessn	Current Smoker
745H.	Smoking cessation therapy	Current Smoker
745H0	Nic rep thera us nicot ptches	Current Smoker
745H1	Nicot repl thera us nicot gum	Current Smoker
745H2	Nic repl thera us nic inhalatr	Current Smoker
745H3	Nic repl thera us nic lozenges	Current Smoker
745H4	Smoking cessation drug therapy	Current Smoker
745Hy	OS smoking cessation therapy	Current Smoker
745Hz	Smoking cessation therapy NOS	Current Smoker
8CAg.	Smoke cess advi prov com pharm	Current Smoker
8CAL.	Smoking cessation advice	Current Smoker
8CdB.	Stop smok serv opport signpost	Current Smoker
8H7i.	Referral: smok cessatn advisor	Current Smoker
8HBM.	Stop smoking fce to fce flw-up	Current Smoker
8HBP.	Smoking cesn 12 week follow-up	Current Smoker
8HkQ.	Refer to NHS stop smoking srvc	Current Smoker
8HTK.	Referl to stop-smoking clinic	Current Smoker
8IAj.	Smok cessation advice declined	Current Smoker
8IEK.	Smok cessation program declned	Current Smoker
8IEM.	Smoking cess drug therapy decl	Current Smoker
8IEo.	Ref smoking cessn serv declnd	Current Smoker
8T08.	Ref to smoking cessn service	Current Smoker
9hG..	Except repor: smoking qual ind	Current Smoker
9hG0.	Except smok qual ind: Pt uns	Current Smoker
9hG1.	Except smok qual ind: Inf diss	Current Smoker
9kc..	Smoking cessation - enh se adm	Current Smoker
9kc0.	Smok ces templt completd - ESA	Current Smoker
9kf1.	Ref COPD stru smok asses - ESA	Current Smoker
9kf2.	COPD stru smok ass decld - ESA	Current Smoker
9ko..	Currnt smokr annua reviw - ESA	Current Smoker
9N2k.	Seen by smoking cesstn advisor	Current Smoker
9N4M.	DNA - Smoking cessation clinic	Current Smoker
9Ndg.	Dec con f flw up by smk cess t	Current Smoker
9NdZ.	Dec con for smok cess data sha	Current Smoker
9NS02	Ref smoking cessat serv offer	Current Smoker
9OO..	Stop smoking monitoring admin.	Current Smoker
9OO1.	Attends stop smoking monitor.	Current Smoker
9OO2.	Refuses stop smoking monitor	Current Smoker
9OO3.	Stop smoking monitor default	Current Smoker
9OO4.	Stop smoking monitor 1st lettr	Current Smoker
9OO5.	Stop smoking monitor 2nd lettr	Current Smoker
9OO6.	Stop smoking monitor 3rd lettr	Current Smoker
9OO7.	Stop smoking monitor verb.inv.	Current Smoker
9OO8.	Stop smoking monitor phone inv	Current Smoker
9OO9.	Stop smoking monitoring delete	Current Smoker
9OOA.	Stop smoking monitor.chck done	Current Smoker
9OOB.	Stop smokg invtn SMS txt mssge	Current Smoker
9OOB0	Stop smoking ivtn 1st txt msge	Current Smoker
9OOB1	Stop smoking ivtn 2nd txt msge	Current Smoker
9OOB2	Stop smoking ivtn 3rd txt msge	Current Smoker

9OOZ.	Stop smoking monitor admin.NOS	Current Smoker
1377.	Ex-trivial smoker (<1/day)	Ex-Smoker
1378.	Ex-light smoker (1-9/day)	Ex-Smoker
1379.	Ex-moderate smoker (10-19/day)	Ex-Smoker
137A.	Ex-heavy smoker (20-39/day)	Ex-Smoker
137B.	Ex-very heavy smoker (40+/day)	Ex-Smoker
137F.	Ex-smoker - amount unknown	Ex-Smoker
137j.	Ex-cigarette smoker	Ex-Smoker
137K.	Stopped smoking	Ex-Smoker
137K0	Recently stopped smoking	Ex-Smoker
137l.	Ex roll-up cigarette smoker	Ex-Smoker
137L.	Current non-smoker	Ex-Smoker
137N.	Ex pipe smoker	Ex-Smoker
137O.	Ex cigar smoker	Ex-Smoker
137S.	Ex smoker	Ex-Smoker
137T.	Date ceased smoking	Ex-Smoker
13p4.	Smoking free weeks	Ex-Smoker
9km..	Ex-smoker annual review - ESA	Ex-Smoker
1371.	Never smoked tobacco	Never Smoked

Compiling the DOSE index: Algorithmic calculation

Patients required at least one valid Obstruction record to qualify for entry to the algorithm. Having selected a qualifying Obstruction record, the patient's clinical record was then reviewed (as detailed above) to identify and score attendant Dyspnoea, Smoking and Exacerbation records occurring during the preceding year (defined as 365 days). For Dyspnoea and Smoking components, any valid record occurring on the same date as the Obstruction record was selected, or, if absent, the closest record within the preceding year. For Exacerbation the total number of distinct incident episodes during the preceding year was counted.

Having identified a single Dyspnoea and Smoking record and the annual number of Exacerbations within the year preceding the qualifying Obstruction record, the composite index score was calculated by totalling the associated component scores according to the table below [10]. The composite score had maximum range 0 to 8 points, and was stratified into three risk groups at ≤3 (low risk), 4-5 (moderate risk) and ≥6 points (high risk) [11].

		Score allocation			
		**0 points**	**1 point**	**2 points**	**3 points**
**Component**	**MRC dyspnoea scale** (Dyspnoea)	0-1	2	3	4
	**FEV_1% predicted** (Obstruction)	≥ 50%	30%-50%	≤ 30%	n/a
	**Smoking status** (Smoking)	Non-smoker	Smoker	n/a	n/a
	**Number of exacerbations** (Exacerbation)	0-1	2-3	≥ 3	n/a

The first DOSE score instance was associated with the earliest qualifying Obstruction record during the study period, and could take any date between 1st January 2010 and 31st December 2014. Subsequent instances were associated with the earliest qualifying Obstruction record a minimum of one year after the previous and a maximum of four years after the first.

Comorbidities

Unlike the approach to defining the cohort, our comorbidity code lists included both diagnostic and 'process of care' Read codes. This was to prioritise sensitivity over specificity, thereby increasing our confidence that patients without codes for the comorbidity had not received this diagnosis. Code lists were created independently by three clinicians, with the final list derived by consensus, justifying inclusion of each code if it alone suggested a very high likelihood that the patient had the comorbid disease in question. Codes for medication specific to dementia (Donepezil/Aricept) and idiopathic pulmonary fibrosis (Pirfenidone/Esbriet) were included in code lists for these comorbidities, as their use is specific to these conditions.

Code	Description
**Asthma**	
173A.	Exercise induced asthma
173c.	Occupational asthma
173d.	Work aggravated asthma
1780.	Aspirin induced asthma
1781.	Asthma trigger - pollen
1782.	Asthma trigger - tobacco smoke
1783.	Asthma trigger - warm air
1784.	Asthma trigger - emotion
1785.	Asthma trigger - damp
1786.	Asthma trigger - animals
1787.	Asthma trigger - seasonal
1788.	Asthma trigger - cold air
1789.	Asthma trigger respiratory inf
178A.	Asthma trigger - airborne dust
178B.	Asthma trigger - exercise
1O2..	Asthma confirmed
388t.	RCP asthma assessment
38DL.	Asthma control test
38DT.	Asthma control questionnaire
38DV.	Mini asthma QOL questionnaire
661M1	Asthma self-manage plan agreed
661N1	Asthma self-manage plan review
663d.	Emerg asthm adm since lst appt
663e.	Asthma restricts exercise
663e000	Asthma sometime restr exercise
663e100	Asthma severely restr exercise
663f.	Asthma never restrcts exercise
663j.	Asthma - currently active
663m.	Asth A&E attend since last vis
663n.	Asth treat compliance satisfac
663N.	Asthma disturbing sleep
663N0	Asthma causing night waking
663N1	Asthma disturbs sleep weekly
663N2	Asthma disturbs sleep freqntly
663O.	Asthma not disturbing sleep
663O0	Asthma never disturbs sleep
663p.	Asth treat compliance unsatisf
663P.	Asthma limiting activities
663P0	Asthma limit act 1-2 time mth
663P1	Asth limit activ 1 - 2 time wk
663P2	Asthma limits activit most day
663q.	Asthma daytime symptoms
663Q.	Asthma not limiting activities
663r.	Asthma night symp 1-2 per mth

663s.	Asthma never causes day symps
663t.	Asthma day symp 1-2 per mth
663u.	Asthma day symp 1-2 per week
663U.	Asthma management plan given
663v.	Asthma daytime symps most days
663V.	Asthma severity
663V0	Occasional asthma
663V1	Mild asthma
663V2	Moderate asthma
663V3	Severe asthma
663w.	Asthm limits walk hills/stairs
663x.	Asthma limits walking on flat
663y.	Num asthm exacs in past year
66Y5.	Change in asthma managemt plan
66Y9.	Step up chnge asthm managmt pl
66YA.	Step down chnge asthm manag pl
66YC.	Absent work/schl due to asthma
66YJ.	Asthma annual review
66YK.	Asthma follow-up
66Yp.	Asthma review RCP 3 questions
66YP.	Asthma night-time symptoms
66Yq.	Asthma night symptom 1 to 2 wk
66YQ.	Asthma monitoring by nurse
66Yr.	Asthma cause sympt most nights
66YR.	Asthma monitoring by doctor
66Ys.	Asthma never caus night symptm
66Yu.	Num dy abs sch asthma pst 6 mn
8793.	Asthma control step 0
8794.	Asthma control step 1
8795.	Asthma control step 2
8796.	Asthma control step 3
8797.	Asthma control step 4
8798.	Asthma control step 5
8B3j.	Asthma medication review
8CMA0	Pat writt asthma pers act plan
8CR0.	Asthma clin management plan
8H2P.	Emergency admission
9hA..	Except report: asthma qual ind
9hA1.	Except asthma qual ind: Pt uns
9hA2.	Excep asthma qual ind: Inf dis
9OJA.	Asthma monitoring check done
H3120	Chronic asthmatic bronchitis
H33..	Asthma
H330.	Extrinsic (atopic) asthma
H3300	Extrinsic asthma - no status
H3301	Extrinsic asthma + status
H330z	Extrinsic asthma NOS
H331.	Intrinsic asthma
H3310	Intrinsic asthma - no status
H3311	Intrinsic asthma + status
H331z	Intrinsic asthma NOS
H332.	Mixed asthma
H333.	Acute exacerbation of asthma
H334.	Brittle asthma
H335.	Chron asthm w fix airflw obstr
H33z.	Asthma unspecified
H33z0	Status asthmaticus NOS
H33z1	Asthma attack

H33z2	Late-onset asthma
H33zz	Asthma NOS
H35y6	Sequoiosis (red-cedar asthma)
H35y7	Wood asthma
H47y0	Detergent asthma
**Ischaemic Heart Disease**	
14A3.	H/O: myocardial infarct <60
14A4.	H/O: myocardial infarct >60
14AH.	H/O: Myoc infarct in last year
14AT.	H/O: myocardial infarction
14AW.	H/O acute coronary syndrome
187..	Frequency of angina
323..	ECG: myocardial infarction
3232.	ECG: old myocardial infarction
3233.	ECG: antero-septal infarct.
3234.	ECG:posterior/inferior infarct
3235.	ECG: subendocardial infarct
3236.	ECG: lateral infarction
323Z.	ECG: myocardial infarct NOS
3889.	Euroscore for angina
388E.	Canad Card Soc classif angina
388F.	CLASP angina score
661M0	Angina self-manage plan agreed
661N0	Angina self-manage plan review
662K.	Angina control
662K0	Angina control - good
662K1	Angina control - poor
662K2	Angina control - improving
662K3	Angina control - worsening
662K4	Angina self manage pln commenc
662K5	Angina self manage pln complet
662Kz	Angina control NOS
662N.	CHD monitoring
6A2..	Corony heart dis annual review
6A4..	Coronary heart disease review
7920.	Saphen v graft repl coronary a
79200	Saphen v graft repl 1 cor art
79201	Saphen v graft repl 2 cor art
79202	Saphen v graft repl 3 cor art
79203	Saphen v graft repl 4+ cor art
7920y	Saph vein graft repl cor a OS
7920z	Saph vein graft repl cor a NOS
7921.	Other autograft rep coronary a
79210	Autograft rep 1 coronary a NEC
79211	Autograft rep 2 coronary a NEC
79212	Autograft rep 3 coronary a NEC
79213	Autogr repl 4+ coronary a NEC
7921y	Other autograft repl cor a OS
7921z	Other autograft repl cor a NOS
7922.	Allograft replac coronary art
79220	Allograft replac 1 coronary a
79221	Allograft replac 2 coronary a
79222	Allograft replac 3 coronary a
79223	Allograft replac 4+ coronary a
7922y	Allograft replac coronary a OS
7922z	Allograft replac coronry a NOS
7923.	Prosth replac coronary artery
79230	Prosth replac 1 coronary art

79231	Prosth replac 2 coronary art
79232	Prosth replac 3 coronary art
79233	Prosth replac 4+ coronary art
7923y	Prosth replac coronary art OS
7923z	Prosth replac coronary art NOS
7924.	Revision bypass coronary art
79240	Revision bypass 1 coronary art
79241	Revision bypass 2 coronary art
79242	Revision bypass 3 coronary art
79243	Revision bypass 4 coronary art
79244	Rev conn thoracic to coronary
79245	Rev implant thorac a in heart
7924y	Revision bypass coronary a OS
7924z	Revision bypass coronary a NOS
7925.	Connct mammary to coronary art
79250	Double anast mamm/coronary art
79251	Doub implant mamm/coronary art
79252	Sing anast mamm l ant desc cor
79253	Single anast mamm/coronary NEC
79254	Single implant mamm/coronary a
7925y	Connect mammary coronary a OS
7925z	Connect mammary coronary NOS
7926.	Connect oth thor art coron art
79260	Double anast thor/cor art NEC
79261	Double implant thor/cor a NEC
79262	Single anast thor/cor art NEC
79263	Single implant thor/cor a NEC
7926y	Connect oth thor/coron art OS
7926z	Connect oth thor/coron art NOS
79275	Open angioplasty coronary art
7928.	Translum balloon angiop coro a
79280	Percut balloon angiopl 1 cor a
79281	PC balloon angiopl mult cor ar
79282	PC balloon angiopl cor a graft
79283	Per tran cut bal angio cor art
7928y	TL balloon angiopl coron a OS
7928z	TL balloon angiopl coron a NOS
79290	PC TL laser coronary angioplas
79291	Streptok PC TL cor thrombolys
79292	PC TL inj therap sub cor a NEC
79293	Rotary coronary angioplasty
79294	Insert coronary artery stent
79295	Insert drug-elut cor art stent
79296	Percut translum ather cor art
792B0	Endarterectomy coronary a NEC
792D.	Other bypass coronary artery
792Dy	Other bypass coronary art OS
792Dz	Other bypass coronary art NOS
792E.	Percutan coronary interventn
792E0	Emrgncy percut coronry intrvnt
793G.	Perc tran bal ang sten cor art
793G0	P t ba an in 1-2 dr el st co a
793G1	P t b an in 3 mo dr el st co a
793G2	Pe tr bal an in 1-2 ste co art
793G3	Pe co ba ang 3 mo st co ar NEC
793Gy	OS per tra bal ang ste cor art
793Gz	Per tra bal ang ste cor ar NOS
8B27.	Antianginal therapy

8B3k.	CHD medication review
8CMP.	Coronry hart disease care plan
8H2V.	Admit isch heart dis emergency
8IEY.	Rf Ang Pln slf-mgt prg decld
8T04.	Ref Angina Plan self-mgt prg
9Ob0.	Attends CHD monitoring
9Ob1.	Refuses CHD monitoring
9Ob8.	CHD monitoring check done
G3...	Ischaemic heart disease
G30..	Acute myocardial infarction
G300.	Acute anterolateral infarction
G301.	Anterior myocard. infarct OS
G3010	Acute anteroapical infarction
G3011	Acute anteroseptal infarction
G301z	Anterior myocard.infarct NOS
G302.	Acute inferolateral infarction
G303.	Acute inferoposterior infarct
G304.	Posterior myocard.infarct NOS
G305.	Lateral myocardial infarct NOS
G306.	True posterior myocard.infarct
G307.	Acute subendocardial infarct
G3070	Acute non-Q wave infarction
G3071	Acute non-ST seg elevation mi
G308.	Inferior myocard. infarct NOS
G309.	Acute Q-wave infarct
G30B.	Acute posterol myocard infarct
G30X.	Ac transmur MI unspec site
G30X0	Acute ST segment elevation mi
G30y.	Other acute myocardial infarct
G30y0	Acute atrial infarction
G30y1	Acute papillary muscle infarct
G30y2	Acute septal infarction
G30yz	Other acute myocardial inf.NOS
G30z.	Acute myocardial infarct. NOS
G31..	Other acute/subacute IHD
G311.	Preinfarction syndrome
G3110	Myocardial infarction aborted
G3111	Unstable angina
G3112	Angina at rest
G3113	Refractory angina
G3114	Worsening angina
G3115	Acute coronary syndrome
G311z	Preinfarction syndrome NOS
G312.	Coron thromb/not result in MI
G31y.	Other acute/subacute IHD
G31y0	Acute coronary insufficiency
G31y1	Microinfarction of heart
G31y2	Subendocardial ischaemia
G31y3	Transient myocardial ischaemia
G31yz	Other acute/subacute IHD NOS
G32..	Old myocardial infarction
G33..	Angina pectoris
G330.	Angina decubitus
G3300	Nocturnal angina
G330z	Angina decubitus NOS
G33z.	Angina pectoris NOS
G33z0	Status anginosus
G33z1	Stenocardia

G33z2	Syncope anginosa
G33z3	Angina on effort
G33z4	Ischaemic chest pain
G33z5	Post infarct angina
G33z6	New onset angina
G33z7	Stable angina
G33zz	Angina pectoris NOS
G34..	Other chr.ischaemic heart dis.
G340.	Coronary atherosclerosis
G3400	Single coronary vessel disease
G3401	Double coronary vessel disease
G342.	Atherosclerotic cardiovasc dis
G343.	Ischaemic cardiomyopathy
G344.	Silent myocardial ischaemia
G34y.	Other specif. chronic IHD
G34y0	Chronic coronary insufficiency
G34y1	Chronic myocardial ischaemia
G34yz	Other specif.chronic IHD NOS
G34z.	Other chronic IHD NOS
G34z0	Asymptomatic CHD
G35..	Subseqnt myocardial infarction
G350.	Subsqnt myocrd infarc/ant wall
G351.	Subsqnt myocrd infarc/inf wall
G353.	Subseq myo infarct other sites
G35X.	Subseq MI of unspec site
G36..	Certain curnt comp fol acut MI
G360.	Haempericrd/cur comp fol ac MI
G361.	Atrl sept def/c comp fol ac MI
G362.	Vent sep def/c comp fol ac MI
G363.	Rup cd w w't h'mpc/cmp f ac MI
G364.	Rp chord tend/c cmp fol ac MI
G365.	Rp papilr musc/c cmp fol ac MI
G366.	Thrm/atr
G38..	Postoperative MI
G380.	Postop transmur MI inf wall
G381.	Postop transm MI inferior wall
G382.	Postop transm MI other sites
G383.	Postop transm MI unspec site
G384.	Postop subendocardial MI
G38z.	Postop MI
G39..	Coronary microvascular disease
G3y..	Ischaemic heart disease OS
G3z..	Ischaemic heart disease NOS
G501.	Post infarction pericarditis
Gyu3.	[X]Ischaemic heart diseases
Gyu30	[X]Other forms/angina pectoris
Gyu31	[X]Oth currnt comp follw ac MI
Gyu32	[X]Oth form/ac ischaem hrt dis
Gyu33	[X]O form/chron ischmc hrt dis
Gyu34	[X]Ac transmur MI unspec site
Gyu35	[X]Subseq myocard inf oth site
Gyu36	[X]Subseq MI of unspec site
SP076	Coronary art bypass graft occl
ZV457	[V]Pres/aortcoronry bypss grft
ZV458	[V]Pres/coron angiopl impl+gft
ZV45K	[V]Pres coron art bypass graft
ZV45L	[V]Stat foll coron angiopl NOS
**Heart Failure**	

14A6.	H/O: heart failure
14AM.	H/O:Heart failure in last year
183B.	Worsening pulmonary oedema
1O1..	Heart failure confirmed
23E1.	O/E - pulmonary oedema
33BA.	Impaired left ventricular func
388D.	NYHA classif heart fail symps
585f.	Echocardiogram shows LVSDF
585g.	Echocardiogram shows LVDDF
661M5	Heart fail slf-manag pln agree
661N5	Heart fail self-manag pln revw
662f.	NYHA classification - class I
662g.	NYHA classification - class II
662h.	NYHA classification- class III
662i.	NYHA classification - class IV
662p.	Heart failure 6 month review
662T.	Congestive heart failure monit
662W.	Heart failure annual review
679W1	Educatn deterior heart failure
679X.	Heart failure education
67D4.	Heart failure info given to pt
7936J	Imp intr biventric car pace sy
79379	Implant bivent card pacemak sy
8B29.	Cardiac failure therapy
8CeC.	Pref pl cre nxt exacerbatin HF
8CL3.	HF care plan discussed with pt
8CMK.	Has heart failure manage plan
8CMW8	Heart failure clinical pathway
8H2S.	Admit heart failure emergency
8HBE.	Heart failure follow-up
8HHb.	Referral to heart failure nurs
8HHz.	Ref to heart failur exerc prog
8Hk0.	Refd - heart fail educat group
8HTL.	Referral heart failure clinic
8HTL0	Ref rapid acess hert fail clnc
8IE0.	Ref heart fail eductn grp dcld
8IE1.	Ref heart failur exer prog dec
9hH1.	Ex heart fai qual ind: Inf dis
9N0k.	Seen in heart failure clinic
9N2p.	Seen by comm heart failur nurs
9Or0.	Heart failure review completed
G1yz1	Rheumatic left ventric.failure
G2101	Malig.hypert.heart dis.-+ CCF
G2111	Benign hypert.heart dis-+ CCF
G234.	Hyp ht&ren d+both(con)h&r fail
G58..	Heart failure
G580.	Congestive heart failure
G5800	Acute congestive heart failure
G5801	Chroncongestive heart failure
G5802	Decompensated cardiac failure
G5803	Compensated cardiac failure
G5804	Cong heart fail due valv dis
G581.	Left ventricular failure
G5810	Acute left ventricular failure
G582.	Acute heart failure
G583.	Heart failure norm eject frac
G58z.	Heart failure NOS
G5yy9	Left ventricul systol dysfunc

G5yyA	Left ventric diastolic dysfunc
G5yyC	Diastolic dysfunction
G5yyD	Lft ventr cardiac dysfunction
Q48y1	Congenital cardiac failure
R2y10	[D]Cardiorespiratory failure
SP111	Cardiac insuffic.comp.of care
SP112	Cardioresp.fail.comp.of care
ZV45M	[V]Biventric pacemaker in situ
**Cor pulmonale**	
G400.	Acute cor pulmonale
G41z.	Chronic pulmon. heart dis. NOS
G584.	Right ventricular failure
G5yyB	Right ventric diastol dysfunct
G5yyE	Rt ventrc systolic dysfunction
**Hypertension**	
6627.	Good hypertension control
6628.	Poor hypertension control
662b.	Moderate hypertension control
662c.	Hypertension six month review
662d.	Hypertension annual review
662O.	On treatment for hypertension
662P0	Hypertension 9 month review
8BL0.	Pt on max tol antihypert ther
G2...	Hypertensive disease
G20..	Essential hypertension
G200.	Malignant essential hypertens.
G201.	Benign essential hypertension
G202.	Systolic hypertension
G203.	Diastolic hypertension
G20z.	Essential hypertension NOS
G21..	Hypertensive heart disease
G210.	Malignant hypertens.heart dis.
G2100	Malig.hypert.heart dis.-no CCF
G2101	Malig.hypert.heart dis.-+ CCF
G210z	Malig.hypertens.heart dis. NOS
G211.	Benign hypertensive heart dis.
G2110	Benign hypert.heart dis-no CCF
G2111	Benign hypert.heart dis-+ CCF
G211z	Benign hypertens.heart dis.NOS
G21z.	Hypertensive heart disease NOS
G21z0	Hypertens.heart dis.NOS-no CCF
G21z1	Hypertens.heart dis.NOS- + CCF
G21zz	Hypertensive heart disease NOS
G22..	Hypertensive renal disease
G220.	Malignant hypertens.renal dis.
G221.	Benign hypertensive renal dis.
G222.	Hypertens renal dis+renal fail
G22z.	Hypertensive renal disease NOS
G23..	Hypertensive heart+renal dis.
G230.	Malig.hypert.heart+renal dis.
G231.	Benign hypert.heart+renal dis.
G232.	Hypert ht&ren d+(congs)ht fail
G233.	Hypertn hrt&ren dis+renal fail
G234.	Hyp ht&ren d+both(con)h&r fail
G23z.	Hypertens.heart+renal dis.NOS
G24..	Secondary hypertension
G240.	Secondary malignant hypertens.
G2400	Second.malig.renovasc.hypert.

G240z	Secondary malign.hypertens.NOS
G241.	Secondary benign hypertension
G2410	Second.benign renovasc.hypert.
G241z	Secondary benign hypertens.NOS
G244.	Hypertens 2ndry endocrin disor
G24z.	Secondary hypertension NOS
G24z0	Secondary renovasc.hypert. NOS
G24zz	Secondary hypertension NOS
G25..	Stge 1 hypertensin (NICE 2011)
G250.	Stage 1 hyp wo ev end org dmge
G251.	Stage 1 hyp wi ev end org dmge
G26..	Severe hypertensin (NICE 2011)
G27..	Hypertnsn resistnt to drg ther
G28..	Stge 2 hypertensin (NICE 2011)
G2y..	Hypertensive disease OS
G2z..	Hypertensive disease NOS
G672.	Hypertensive encephalopathy
Gyu2.	[X]Hypertensive diseases
Gyu20	[X]Oth secondary hypertension
Gyu21	[X]Hyperten
**Hyperlipidaemia**	
44O4.	Serum lipids high
44P3.	Serum cholesterol raised
44P4.	Serum cholesterol very high
44Q3.	Serum triglycerides raised
662X.	Target cholesterol level
8BAG1	Cholesterol red progm attended
8BAG2	Cholesterol red progm declined
8BL1.	Pt on max tol lipid low ther
8CR3.	Hyperlipidaemia clin man plan
8HT1.	Referral to lipid clinic
9N0I.	Seen in lipid clinic
9N0J.	Seen in cholesterol clinic
9N4K.	DNA cholesterol clinic
9Oc0.	Attnds lipid disordr monitring
9Oc1.	Lipid disrdr monitring declind
C320.	Pure hypercholesterolaemia
C3200	Familial hypercholesterolaemia
C3201	Hyperbetalipoproteinaemia
C3202	Hyperlipidaemia
C3203	LDL hyperlipoproteinaemia
C3204	Fredrickson type IIa lipidaem
C3205	Fam defect apolipoprot B-100
C3206	Polygenic hypercholesterolemia
C320y	Pure hypercholesterolaemia OS
C320z	Pure hypercholesterolaemia NOS
C321.	Pure hyperglyceridaemia
C3210	Hypertriglyceridaemia
C322.	Mixed hyperlipidaemia
C3220	Familial comb hyperlipidaemia
C323.	Hyperchylomicronaemia
C324.	Hyperlipidaemia NOS
C325.	Lipoprotein deficiencies
C3250	High density lipoid deficiency
C3251	Hypo-alpha-lipoproteinaemia
C325z	Lipoprotein deficiency NOS
C328.	Dyslipidaemia
C329.	Hypercholesterolaemia

C32y4	Lipase deficiency
Cyu8D	[X]Other hyperlipidaemia
**Osteoporosis**	
58EG.	Hip DXA result osteoporotic
58EM.	Lumbar DXA result osteoporotic
58EV.	Femor nec DEXA scan osteoporot
66a..	Osteoporosis monitoring
66a2.	Osteoporosis treatment started
66a4.	Osteoporosis treatment changed
66a5.	Osteoporosis - no treatment
66a6.	Osteoporosis - dietary advice
66a7.	Osteoporosis - diet assessment
66a8.	Osteoporosis - exercise advice
66a9.	Osteoporosis-falls prevention
66aA.	Osteoporosis-treatmnt response
66aB.	Osteoporosis-no treat response
9hP1.	Exc osteo qual ind: infor diss
9kj0.	Bn spr drg trt ofr osteo - ESA
9Od9.	Osteoporosis monit check done
N330.	Osteoporosis
N3300	Osteoporosis
N3301	Senile osteoporosis
N3302	Postmenopausal osteoporosis
N3303	Idiopathic osteoporosis
N3304	Dissuse osteoporosis
N3305	Drug-induced osteoporosis
N3306	Postoophorectomy osteoporosis
N3307	Postsurg malabsorp osteoporos
N3309	Osteopor
N330A	Osteoporosis in endocr disord
N330B	Vertebral osteoporosis
N330C	Osteoporosis localized spine
N330D	Osteoporos due corticosteroid
N330z	Osteoporosis NOS
N3312	Postoophorc osteopor+path frct
N3313	Osteopor of disuse + path frct
N3314	Postsur malab osteop+path frct
N3315	Drug-ind osteopor + path fract
N3316	Idiopath osteopor + path fract
N3318	Osteopor path # lumb vertebrae
N3319	Osteopor path # thor vertebrae
N331A	Osteopor path # cerv vertebrae
N331B	Postmenop osteopor+path fract
N331H	Collap cerv vert due to osteop
N331J	Collap lumb vert due to osteo
N331K	Coll thorac vert due osteopor
N331L	Collap vert due osteopor NOS
N331M	Fragility # unsp osteoporosis
N3746	Osteoporotic kyphosis
NyuB0	[X]Oth osteoporosis+patholog #
NyuB1	[X]Other osteoporosis
NyuB2	[X]Osteoporosis/oth disords CE
NyuB8	[X]Unsp osteopor + pathol frac
**Cerebrovascular disease**	
14AK.	H/O: Stroke in last year
1M4..	Central post-stroke pain
661M7	Stroke self-manage plan agreed
661N7	Stroke self-manage plan review

662e.	Stroke/CVA annual review
662M.	Stroke monitoring
662M1	Stroke 6 month review
662M2	Stroke initial post disch revw
662o.	Haemorrhagic stroke monitoring
7P242	Delivery rehabilitation stroke
8CRB.	TIA clinical management plan
8HHM.	Ref to stroke func improv serv
8IEC.	Ref multidis strk fnc impv dcd
9h2..	Except report: stroke qual ind
9h21.	Except stroke qual ind: Pt uns
9h22.	Exc stroke qual ind: Infor dis
E004.	Arteriosclerotic dementia
E0040	Arterioscl.dementia-uncomplic.
E0041	Arterioscl.dementia+delirium
E0042	Arterioscl.dementia+paranoia
E0043	Arterioscl.dementia+depression
E004z	Arteriosclerotic dementia NOS
Eu01.	[X]Vascular dementia
Eu010	[X]Vascular dement acute onset
Eu011	[X]Multi-infarct dementia
Eu012	[X]Subcortical vascular dement
Eu013	[X]Mix cort/subcor vasc dement
Eu01y	[X]Other vascular dementia
Eu01z	[X]Vascular dementia unspecif
F11x2	Cerebral degen cerebrovasc dis
Fyu55	[X]Oth cerebral TIA's+rel synd
Fyu57	[X]O vasc syn/brain cer vasc d
G6...	Cerebrovascular disease
G61..	Intracerebral haemorrhage
G610.	Cortical haemorrhage
G611.	Internal capsule haemorrhage
G612.	Basal nucleus haemorrhage
G613.	Cerebellar haemorrhage
G614.	Pontine haemorrhage
G615.	Bulbar haemorrhage
G616.	External capsule haemorrhage
G618.	Intracerebrl haem
G619.	Lobar cerebral haemorrhage
G61X.	Intracer haem hemisph
G61X0	Left side intracereb haem unsp
G61X1	Right side intracer haem unsp
G61z.	Intracerebral haemorrhage NOS
G63..	Precerebral arterial occlusion
G630.	Basilar artery occlusion
G631.	Carotid artery occlusion
G632.	Vertebral artery occlusion
G633.	Multip/bilat.precereb.art.occ.
G634.	Carotid artery stenosis
G63y.	Other precerebral artery occl.
G63y0	Cerebr infct/throm/precere art
G63y1	Cerebr infct/embol/precere art
G63z.	Precerebral artery occlus. NOS
G64..	Cerebral arterial occlusion
G640.	Cerebral thrombosis
G6400	Cerebr infct/throm/cerebrl art
G641.	Cerebral embolism
G6410	Cerebr infct/embol/cerebrl art

G64z.	Cerebral infarction NOS
G64z0	Brainstem infarction
G64z1	Wallenberg syndrome
G64z2	Left sided cerebral infarction
G64z3	Right sided cerebral infarct
G64z4	Infarction of basal ganglia
G65..	Transient cerebral ischaemia
G650.	Basilar artery syndrome
G651.	Vertebral artery syndrome
G6510	Vertebro-basilar artery syndrm
G653.	Carotid artery syn hemispheric
G654.	Multi&bilat precerebrl art syn
G656.	Vertebrobasilar insufficiency
G657.	Carotid terr trans ischaem att
G65y.	Other transient cerebral isch.
G65z.	Transient cerebral ischaem.NOS
G65z0	Impending CVA
G65z1	Intermittent CVA
G65zz	Transient cerebral ischaem.NOS
G66..	Stroke/CVA unspecified
G660.	Middle cerebral artery syndrm
G661.	Anterior cerebral artery syn
G662.	Posterior cerebral artery syn
G663.	Brain stem stroke syndrome
G664.	Cerebellar stroke syndrome
G665.	Pure motor lacunar syndrome
G666.	Pure sensory lacunar syndrome
G667.	Left sided CVA
G668.	Right sided CVA
G67..	Other cerebrovascular disease
G670.	Cerebral atherosclerosis
G671.	Generalised isch.CV dis.NOS
G6710	Acute cerebrovasc.insuffic.NOS
G6711	Chronic cerebral ischaemia
G671z	Generalised isch.CV dis.NOS
G677.	Oc/st cere art
G6770	Occlusn+stenos/midl cerebr art
G6771	Occlusn+stenos/anter cereb art
G6772	Occlusn+stenos/post cerebr art
G6773	Occlusn+stenos/cerebellar art
G6774	Occl/sten/mult+bilat cereb art
G679.	Small vessel cerebrovas diseas
G67B.	Revrsb cerbrl vascnstrtn syndr
G67y.	Other cerebrovascular dis OS
G67z.	Other cerebrovasc.disease NOS
G68..	Cerebrovasc.dis.-late effects
G682.	Seq/oth nontraum intrcran haem
G683.	Sequelae/cerebral infarction
G68W.	Seql/o+unspcf cerebvasc dis
G68X.	Seql/strok
G6W..	Cer inf
G6X..	Cereb in/uns oc
G6y..	Cerebrovascular disease OS
G6z..	Cerebrovascular disease NOS
G70y0	Carotid artery atherosclerosis
Gyu6.	[X]Cerebrovascular diseases
Gyu63	[X]Cereb in/uns oc
Gyu64	[X]Other cerebral infarction

Gyu65	[X]Oc+steno/o precerebral artr
Gyu66	[X]Oc+sten/o cerebral arteries
Gyu67	[X]Other spcfd cerebrovasc dis
Gyu6A	[X]Oth cerebrovasc diso/dis CE
Gyu6B	[X]Seql/o n-traum intracrn h'm
Gyu6C	[X]Seql/strok
Gyu6D	[X]Seql/o+unspcf cerebvasc dis
Gyu6G	[X]Cer inf
ZV12D	[V]Pers hist trans isch attack
**Dementia**	
1461.	H/O: dementia
3AE3.	GDS level 4 - mod cog dec
3AE4.	GDS level 5 - mod sev cog dec
3AE5.	GDS level 6 - severe cog dec
3AE6.	GDS level 7 - very sev cog dec
66h..	Dementia monitoring
6AB..	Dementia annual review
8BPa.	Antipsyc drug therapy dementia
8CMG2	Review dementia adv care plan
8CMZ.	Dementia care plan
8CSA.	Dementia advnce cre pln agreed
8Hla.	Referral dementia care advisor
8IAe0	Dementia adv care plan declnd
9hD..	Excep report: demen qual indic
9hD0.	Exc demen qual ind: Pat unsuit
9hD1.	Exc demen qual ind: Inform dis
E000.	Senile dementia-uncomplicated
E001.	Presenile dementia
E0010	Presenile dementia - uncomplic
E0011	Presenile dementia + delirium
E0012	Presenile dementia + paranoia
E0013	Presenile dementia+depression
E001z	Presenile dementia NOS
E002.	Sen.dement.-depressed/paranoid
E0020	Senile dementia + paranoia
E0021	Senile dementia + depression
E002z	Sen.dement.-depr./paranoid NOS
E003.	Senile dementia + delirium
E004.	Arteriosclerotic dementia
E0040	Arterioscl.dementia-uncomplic.
E0041	Arterioscl.dementia+delirium
E0042	Arterioscl.dementia+paranoia
E0043	Arterioscl.dementia+depression
E004z	Arteriosclerotic dementia NOS
E012.	Other alcoholic dementia
E02y1	Drug-induced dementia
E041.	Dementia in conditions EC
Eu00.	[X]Dementia in Alzheimer's
Eu000	[X]Early onset Alzheim dement
Eu001	[X]Late onset Alzheim dementia
Eu002	[X]Atypical/mixed Alzheimer's
Eu00z	[X]Alzheimer's disease unspec
Eu01.	[X]Vascular dementia
Eu010	[X]Vascular dement acute onset
Eu011	[X]Multi-infarct dementia
Eu012	[X]Subcortical vascular dement
Eu013	[X]Mix cort/subcor vasc dement
Eu01y	[X]Other vascular dementia

Eu01z	[X]Vascular dementia unspecif
Eu02.	[X]Dementia in disease EC
Eu020	[X]Dementia in Pick's disease
Eu021	[X]Dement in Creutzfeld-Jakob
Eu022	[X]Dementia in Huntington's
Eu023	[X]Dementia in Parkinson's
Eu024	[X]Dementia in HIV disease
Eu025	[X]Lewy body dementia
Eu02y	[X]Dement
Eu02z	[X] Unspecified dementia
Eu041	[X]Delirium superimp dementia
Eu107	[X]Resid psychotic due alcohol
F110.	Alzheimer's disease
F1100	Alzheimer dis wth early onset
F1101	Alzheimer's dis wth late onset
F111.	Pick's disease
F112.	Senile degeneration of brain
F116.	Lewy body disease
F11x7	Cerebral degen.-Jakob-Creutzf.
Fyu30	[X]Other Alzheimer's disease
AROR23861NEMIS	Aricept Evess Orodispersible tablets 5 mg
AROR23862NEMIS	Aricept Evess Orodispersible tablets 10 mg
ARTA30542EMIS	Aricept Tablets 10 mg
ARTA30543EMIS	Aricept Tablets 5 mg
DOOR23858NEMIS	Donepezil Hydrochloride Orodispersible Tablets (Sugar Free) 5 mg
DOOR23859NEMIS	Donepezil Hydrochloride Orodispersible Tablets (Sugar Free) 10 mg
DOTA30546EMIS	Donepezil Hydrochloride Tablets 10 mg
DOTA30547EMIS	Donepezil Hydrochloride Tablets 5 mg
dy1..	DONEPEZIL HYDROCHLORIDE
dy11.	DONEPEZIL HYDROCHLORIDE 5mg tablets
dy12.	DONEPEZIL HYDROCHLORIDE 10mg tablets
dy13.	ARICEPT 5mg tablets
dy14.	ARICEPT 10mg tablets
dy15.	ARICEPT EVESS 5mg disp tabs
dy16.	ARICEPT EVESS 10mg disp tabs
dy1y.	DONEPEZIL HYDROCHLORIDE 10mg disp tabs
dy1z.	DONEPEZIL HYDROCHLORIDE 5mg disp tabs
**Gastro Oesophageal Reflux Disease**	
171J.	Reflux cough
1952.	Regurgitates food
1953.	Waterbrash
1957.	Gastric reflux
760L.	Antireflux operations
760L0	Antireflux fundoplic thor appr
760L1	Antireflux op thorac appr NEC
760L2	Antireflux fundoplic abd appr
760L3	Antireflux gastropexy
760L4	Antireflux proc & gastropl HFQ
760L5	Insertion Angelchick prosthes
760L6	Oesophagogastric fundoplasty
760L7	Endo Nissen fund thoracic appr
760L8	Lap Nissen fundoplic abdo appr
760Ly	Antireflux operation OS
760Lz	Antireflux operation NOS
760M.	Revision antireflux operations
760M0	Revision fundoplication stom
760M1	Adjustment Angelchick prosthes
760M2	Removal Angelchick prosthesis

760My	Revision antireflux op OS
760Mz	Revision antireflux op NOS
J1011	Reflux oesophagitis
J1020	Peptic ulcer of oesophagus
J1025	Barrett's ulcer of oesophagus
J1034	Peptic stricture of oesophagus
J10y4	Oesoph reflux no oesophagitis
J10y5	Laryngopharyngeal reflux
J10y6	Barrett's oesophagus
R0711	[D]Waterbrash
**Peripheral Vascular Disease**	
14F7.	H/O: arterial lower limb ulcer
1M110	Ischaemic foot pain at rest
1M111	Ischaemic ft pain when walking
2G63.	Ischaemic toe
662U.	Periph vasc disease monitoring
7A121	Bypass bifurc anast fem a NEC
7A123	Bypass bifurc anastom iliac a
7A41.	Other bypass of iliac artery
7A411	Bypass iliac anast il/fem NEC
7A413	Bypass iliac anast fem/fem NEC
7A419	Byp comm iliac anast aort/c il
7A41A	Byp iliac art anast aort/ex il
7A41B	Byp leg art anast aorta/c fem
7A41C	Byp leg art anast aorta/d fem
7A41D	Byp iliac art anast il/il NEC
7A41E	Em bypass iliac art-unsp anast
7A41F	Ilio-fem prosth X-over graft
7A41y	Other bypass of iliac art OS
7A41z	Other bypass of iliac art NOS
7A420	Endartect patch rep iliac art
7A421	Endarterectomy iliac art NEC
7A431	Open embolectomy iliac artery
7A433	Open insert iliac artery stent
7A44.	Transluminal ops on iliac art
7A440	PC transl angioplasty iliac a
7A441	PC transl embolectomy iliac a
7A443	Insertion iliac artery stent
7A444	Per trans ins iliac art stent
7A44y	Transluminal op iliac art OS
7A44z	Transluminal op iliac art NOS
7A48.	Oth bypass femoral/poplit art
7A480	Bypass fem art pr anas/pop NEC
7A481	Bypass pop art pr anas/pop NEC
7A482	Bypass fem art vein gr/pop NEC
7A483	Bypass pop art vein gr/pop NEC
7A484	Bypass fem art pr anas/tib NEC
7A485	Bypass pop art pr anas/tib NEC
7A486	Bypass fem art vein gr/tib NEC
7A487	Bypass pop art vein gr/tib NEC
7A488	Bypass fem art pr anas/per NEC
7A489	Bypass pop art pr anas/per NEC
7A48A	Bypass fem art vein gr/per NEC
7A48B	Bypass pop art vein gr/per NEC
7A48C	Byp fem art anast fem/fem NEC
7A48D	Byp pop art anast pop/fem NEC
7A48E	Fem-fem prosth X-over graft
7A48y	Other bypass fem/poplit art OS

7A48z	Oth bypass fem/pop artery NOS
7A49.	Reconstruction fem/pop artery
7A490	Endarterect patch rep feml art
7A491	Endarterect patch rep popl art
7A492	Endarterect femoral artery NEC
7A493	Endarterect poplit artery NEC
7A494	Profundoplast patch rep fem a
7A495	Profundoplast patch rep popl a
7A496	Profundoplasty femoral art NEC
7A497	Profundoplasty poplit art NEC
7A498	Recon femoral artery vein graf
7A499	Recon popliteal art vein graft
7A49y	Reconstruction fem/pop art OS
7A49z	Reconstruction fem/pop art NOS
7A4A2	Open embolectomy femoral art
7A4A3	Open embolectomy popliteal art
7A4B.	Translum ops fem/poplit artery
7A4B0	PC TL angioplasty femoral art
7A4B1	PC TL angioplasty poplit art
7A4B2	PC TL embolectomy femoral art
7A4B3	PC TL embolectomy poplit art
7A4B8	PC TL thromb fem gr streptokin
7A4B9	Perc trans ins stent fem art
7A4By	Translum op fem/poplit art OS
7A4Bz	Translum op fem/poplit art NOS
9hS1.	Ex f pr art dis qu ind inf dis
C107.	Diab.mell.+periph.circul.dis
C1070	Diab.+periph.circ.dis-juvenile
C1071	Diab.+periph.circ.dis.-adult
C1072	Diabetic gangrene - adult
C1073	IDDM periph circulatory disord
C1074	NIDDM periph circulat disord
C107y	Oth spcf diab mel+per circ cmp
C107z	Diab.+periph.circ.disease NOS
C1086	Insulin depen diab mel+gangren
C108G	IDDM with peripheral angiopath
C1095	Non-insulin dep diab mell+gang
C109F	NIDDM with periph angiopath
C10A5	Malnut-rlt diab mel+per circ c
C10E6	Type 1 diab mell with gangrene
C10EG	Type 1 diab mell+periph angiop
C10F5	Type 2 diab mell + gangrene
C10FF	Type 2 diab mell+perip angiop
G702.	Extremity artery atheroma
G702z	Extremity artery atheroma NOS
G73..	Other peripheral vascular dis.
G731.	Thromboangiitis obliterans
G7310	Buerger's disease
G7311	Presenile gangrene
G731z	Thromboangiitis obliterans NOS
G732.	Peripheral gangrene
G7320	Gangrene of toe
G7321	Gangrene of foot
G733.	Ischaemic foot
G734.	Peripheral arterial disease
G73y.	Other spec.periph.vasc.disease
G73y0	Diabetic peripheral angiopathy
G73y1	Periph.angiopathy

G73yz	Other spec.periph.vasc.dis.NOS
G73z.	Peripheral vascular dis. NOS
G73z0	Intermittent claudication
G73zz	Peripheral vasc.disease NOS
G7424	Embolus/thrombus femoral art.
G7425	Embolus/thromb.popliteal art.
G7426	Embolus/thromb.ant.tibial art.
G7427	Embolus/thromb.dors.pedis art.
G7428	Embolus/thromb.post.tibial art
G7429	Embolus/thromb.leg artery NOS
G74y0	Embolus/thromb.com.iliac art.
G74y1	Embolus/thromb.int.iliac art.
G74y2	Embolus/thromb.ext.iliac art.
G74y3	Embolus/thromb iliac art.unsp.
G76z0	Iliac artery occlusion
G76z1	Femoral artery occlusion
G76z2	Popliteal artery occlusion
G784.	Occlusion of artery lower limb
G7840	Occlusn dorsalis pedis artery
G7841	Occlusn anterior tibial artery
G7842	Occlusn posterior tibial artry
Gyu74	[X]Oth spcf periph vasculr dis
M2710	Ischaemic ulcer diabetic foot
M2713	Arterial leg ulcer
M2714	Mixed venous+artery leg ulcer
R0542	[D]Gangrene of toe in diabetic
R0543	[D]Widespread diab foot gangr
**Connective Tissue Disease**	
66c0.	DMARD monitoring
66HB0	Rheumatoid arthritis annul rev
7P203	Del rehab rheumatoid arthritis
9hR1.	Ex rheum arth qua ind: inf dis
9kN..	DMARD monitor - enh serv admin
9kN0.	Pt DMARD rec iss - enh ser adm
9kN1.	Date DMARD mon ap - enh ser ad
9kN2.	Pt DMARD rec upd - enh ser adm
9kN4.	Pt DMARD recor chec - en se ad
9kN5.	DMARD mn rf bk sc cr - en se a
9kN6.	DMARD monitor prmry care - ESA
9kN7.	DMARD monitor secnd care - ESA
9kN8.	DMARD therapy initiated
9NiK.	DNA hospital DMARD monitor clc
9NiL.	DNA GP DMARD monitoring clinic
9NkF.	Seen in GP DMARD monitor clinc
9NkG.	Seen in com DMARD monitor clnc
9NkH.	Seen in hosp DMARD monitr clnc
9Oe0.	Community DMARD monitor appt
9Oe3.	DNA community DMARD monitoring
9Oe4.	Co DMARD mo record retrn to pt
F3712	Polyneuropathy+rheumatoid arth
F3964	Myopathy+rheumatoid arthritis
F3966	Myopathy + scleroderma
F3967	Myopathy + Sjogren's disease
G5y8.	Rheumatoid myocarditis
G5yA.	Rheumatoid carditis
H570.	Rheumatoid lung
H572.	Lung dis.+ systemic sclerosis
H57y1	Lung dis.+ polymyositis

H57y3	Lung dis.+ Sjogren's disease
H57y4	Lung disease + SLE
H58y7	Interst lung dis conn tiss dis
K01x4	Nephrotic syndrome + SLE
K0B4.	Ren tb-in ds/sys con tis ds
K0B40	Renal tubul-interstit dis SLE
N00..	Diffuse connective tissue dis.
N000.	Systemic lupus erythematosus
N0000	Disseminated lupus erythemat.
N0003	Syst lup eryth + organ/sys inv
N0004	SLE with pericarditis
N0006	Cerebral lupus
N000z	Systemic lupus erythematos.NOS
N001.	Scleroderma
N0010	Progressive systemic sclerosis
N0011	CREST syndrome
N002.	Sicca (Sjogren's) syndrome
N003.	Dermatomyositis
N003X	Dermatopolymyositis
N004.	Polymyositis
N04..	Rheumatoid arthritis+similar
N040.	Rheumatoid arthritis
N0400	Rheumatoid arthritis-Cx spine
N0401	Oth rheumatoid arthritis-spine
N0402	Rheumatoid arthritis-shoulder
N0403	Rheumatoid arthr-sternoclav jt
N0404	Rheumatoid arthr-acromioclav j
N0405	Rheumatoid arthritis of elbow
N0406	Rheumatoid arthritis-dist RUJ
N0407	Rheumatoid arthritis of wrist
N0408	Rheumatoid arthritis-MCP joint
N0409	Rheumatoid arthritis-PIPJ-fing
N040A	Rheumatoid arthritis-DIPJ-fing
N040B	Rheumatoid arthritis of hip
N040C	Rheumatoid arthritis of SIJ
N040D	Rheumatoid arthritis of knee
N040E	Rheumatoid arthr of tib-fib jt
N040F	Rheumatoid arthritis of ankle
N040G	Rheumatoid arthr-subtalar jnt
N040H	Rheumatoid arthr-talonav joint
N040J	Rheumatoid arthr-oth tarsal jt
N040K	Rheumatoid arthr-1st MTP joint
N040L	Rheumatoid arthr-lesser MTP jt
N040M	Rheumatoid arthr-IP joint-toe
N040N	Rheumatoid vasculitis
N040P	Seronegative rheumat arthritis
N040Q	Rheumatoid bursitis
N040S	Rheumat arthr - multiple joint
N040T	Flare of rheumatoid arthritis
N041.	Felty's syndrome
N042.	Other rh.arthr.+visc/syst.dis.
N0421	Rheumatoid lung disease
N042z	Rh.arthr.+visc/syst.dis.NOS
N047.	Seropositive errosive RA
N04X.	Seroposit rheum arthr
N04y0	Rheumatoid lung
N2334	Antisynthetase syndrome
N33z5	Relapsing polychondritis

Nyu10	[X]Rheum arthrit+inv/o org/sys
Nyu11	[X]O sero+ve rheumat arthritis
Nyu12	[X]Oth spcf rheumatd arthritis
Nyu1G	[X]Seroposit rheum arthr
Nyu4.	[X]Systmc connctv tis disordrs
Nyu43	[X]Oth forms/sys lup erythemat
Nyu44	[X]Other dermatomyositis
Nyu45	[X]Oth forms/systemc sclerosis
Nyu46	[X]Other overlap syndromes
Nyu47	[X]Oth syst dis/connctv tissue
Nyu48	[X]Dermat(poly)myosit/neo d CE
Nyu4C	[X]Sys diso/connctv t/o dis CE
Nyu4E	[X]Dermatopolymyositis
Nyu4F	[X]Mixed connect tiss disease
**Anxiety/Depression**	
1465.	H/O: depression
1466.	H/O: anxiety state
173f.	Anxiety about breathlessness
1B13.	Anxiousness
1B17.	Depressed
1B1U.	Symptoms of depression
1B1V.	C/O - panic attack
1BT..	Depressed mood
1JJ..	Suspected depression
212S.	Depression resolved
2257.	O/E - depressed
38Dp.	HAMD-Hamil rating scal depress
62T1.	Puerperal depression
66590	Antidepress drug treat started
6G00.	Postnatal depression counsel
8BK0.	Depression management program
8CAa.	Pt given adv manag depression
8G94.	Anxiety management training
8HHp.	Ref guid self-help for anxiety
8HHq.	Ref guid self-help for depress
9H90.	Depression annual review
9H91.	Depression medication review
9H92.	Depression interim review
9HA0.	On depression register
9hC1.	Exc depressi qual ind: Inf dis
9k4..	Depression - enhanc ser admin
9k40.	Depression - enh serv complete
9kQ..	On fl ds lg trt dep - en se ad
E0013	Presenile dementia+depression
E002.	Sen.dement.-depressed/paranoid
E0021	Senile dementia + depression
E0043	Arterioscl.dementia+depression
E02y3	Drug-induced depressive state
E11..	Affective psychoses
E112.	Single major depressive episod
E1120	Single major depression-unspec
E1121	Single major depression-mild
E1122	Single major depress.-moderate
E1123	Single major depression-severe
E1124	Single maj.depress.severe+psyc
E1125	Single maj.depres.-part remiss
E1126	Single maj.depres.-full remiss
E112z	Single major depression NOS

E113.	Recurrent major depressive epi
E1130	Recurr.major depression-unspec
E1131	Recurr.major depression-mild
E1132	Recurr.major depress.-moderate
E1133	Recurr.major depression-severe
E1134	Recurr.maj.depres.-severe+psyc
E1135	Recurr.maj.depres.-part remiss
E1136	Recurr.maj.depres.-full remiss
E1137	Recurrent depression
E113z	Recurr. major depression NOS
E114.	Bipolar affective - now manic
E1140	Manic bipolar affective-unspec
E1141	Manic bipolar affective-mild
E1142	Manic bipolar affect.-moderate
E1143	Manic bipolar affect.-severe
E1144	Manic bipol.affect.severe+psyc
E1145	Manic bipol.affect.part remiss
E1146	Manic bipol.affect.full remiss
E114z	Manic bipolar affective NOS
E115.	Bipolar affective - now depres
E1150	Depressed bipolar affect.-unsp
E1151	Depress.bipolar affect.-mild
E1152	Depr.bipolar affect.-moderate
E1153	Depr.bipolar affect.-severe
E1154	Depr.bipol.affect.-severe+psyc
E1155	Depr.bipol.affect.-part remiss
E1156	Depr.bipol.affect.-full remiss
E115z	Depressed bipolar affect. NOS
E116.	Mixed bipolar affective disord
E1160	Mixed bipolar affective-unspec
E1161	Mixed bipolar affective-mild
E1162	Mixed bipolar affect.-moderate
E1163	Mixed bipolar affect.-severe
E1164	Mixed bipol.affect.severe+psyc
E1165	Mixed bipol.affect.part remiss
E1166	Mixed bipol.affect.full remiss
E116z	Mixed bipolar affective NOS
E117.	Unspec bipolar affect disord
E1170	Unspecified bipolar affective
E1171	Unsp.bipolar affective-mild
E1172	Unsp.bipolar affect.-moderate
E1173	Unsp.bipolar affect.-severe
E1174	Unsp.bipol.affect.-severe+psyc
E1175	Unsp.bipol.affect.-part remiss
E1176	Unsp.bipol.affect.-full remiss
E117z	Unspecif.bipolar affective NOS
E118.	Seasonal affective disorder
E11y.	Other manic-depressive psychos
E11y0	Unspec manic-depressive psycho
E11y2	Atypical depressive disorder
E11y3	Other mixed manic-depres psych
E11z2	Masked depression
E130.	Reactive depressive psychosis
E135.	Agitated depression
E200.	Anxiety states
E2000	Anxiety state unspecified
E2001	Panic disorder
E2002	Generalised anxiety disorder

E2003	Anxiety with depression
E2004	Chronic anxiety
E2005	Recurrent anxiety
E200z	Anxiety state NOS
E202.	Phobic disorders
E204.	Neurotic (reactive) depression
E2112	Depressive personality disord
E290.	Brief depressive reaction
E290z	Brief depressive reaction NOS
E291.	Prolonged depressive reaction
E2B..	Depressive disorder NEC
E2B1.	Chronic depression
E2D0.	Anxiety/fear child/adoles.dis.
E2D00	Child/adolesc.overanxious.dis.
E2D01	Child/adolesc.fearfulness dis.
E2D0z	Anxiety/fear child/adolesc.NOS
Eu054	[X]Organic anxiety disorder
Eu204	[X]Post-schizophrenic depressn
Eu251	[X]Schzaffctve dis depres type
Eu31.	[X]Bipolar affective disorder
Eu310	[X]Bipol affec current hypoman
Eu311	[X]Bipol aff
Eu312	[X]Bipol affect manic+psychos
Eu313	[X]Bipol aff mild/mod depress
Eu314	[X]Bipol AD
Eu315	[X]Bipol aff sev depress/psych
Eu316	[X]Bipol affective dis
Eu317	[X]Bipol affect dis remission
Eu318	[X]Bipol affect disord type I
Eu319	[X]Bipol affect disord type II
Eu31y	[X]Oth bipolar affectve disord
Eu32.	[X]Depressive episode
Eu320	[X]Mild depressive episode
Eu321	[X]Moderate depressve episode
Eu322	[X]Severe depressiv no psychot
Eu323	[X]Severe depressive + psychot
Eu324	[X]Mild depression
Eu325	[X]Major depression
Eu326	[X]Major depression
Eu327	[X]Maj dep
Eu328	[X]Maj dep
Eu329	[X]Sin ma dep ep sev ps ps rem
Eu32A	[X]Rec ma dep ep sev ps ps rem
Eu32B	[X]Antenatal depression
Eu32y	[X]Other depressive episodes
Eu32z	[X]Depressive episode
Eu33.	[X]Recurrent depressive disord
Eu330	[X]Recurr depress current mild
Eu331	[X]Recurr depress current mod
Eu332	[X]Recurr dep now sever no psy
Eu333	[X]Recurr dep now sever+psych
Eu334	[X]Recurr depress in remission
Eu33y	[X]Oth recurr depressve disord
Eu33z	[X]Recurrent depress dis unsp
Eu341	[X]Dysthymia
Eu3y1	[X]Oth recurr mood affect dis
Eu40.	[X]Phobic anxiety disorders
Eu40y	[X]Other phobic anxiety disord

Eu40z	[X]Phobic anxiety disordr unsp
Eu41.	[X]Other anxiety disorders
Eu410	[X]Panic episodic paroxysm anx
Eu411	[X]Generalized anxiety disord
Eu412	[X]Mixed anxiety/depressve dis
Eu413	[X]Other mixed anxiety disord
Eu41y	[X]Other specif anxiety disord
Eu41z	[X]Anxiety disord unspecified
Eu53.	[X]Puerperal mental disord NEC
Eu530	[X]Mild puerperal ment dis NEC
Eu531	[X]Sever puerper ment dis NEC
Eu606	[X]Anxious
Eu920	[X]Depressive conduct disorder
Eu931	[X]Phobic anxiet dis childhood
Eu932	[X]Social anx dis childhood
ZV111	[V]PH - Affective disorder
**Lung Cancer**	
B22..	Malig neop trachea/bronch/lung
B220.	Malig neop trachea
B2201	Malig neop mucosa of trachea
B220z	Malig neop trachea NOS
B221.	Malig neop main bronchus
B2210	Malig neop carina of bronchus
B2211	Malig neop hilus of lung
B221z	Malig neop main bronchus NOS
B222.	Malig neop upp lobe bronc/lung
B2220	Malig neop upper lobe bronchus
B2221	Malig neop upper lobe of lung
B222z	Malig neop upp bronc/lung NOS
B223.	Malig neop mid lobe bronc/lung
B2230	Malig neop mid lobe bronchus
B2231	Malig neop middle lobe of lung
B223z	Malig neop mid lobe bronc/lung
B224.	Malig neop low lobe bronc/lung
B2240	Malig neop lower bronchus
B2241	Malig neop lower lobe of lung
B224z	Malig neop low lobe bronc/lung
B225.	Mal neop
B22y.	Malig neop oth site bronc/lung
B22z.	Malig neop bronchus/lung NOS
BB5S2	[M]Bronchiolo-alveolar adenoca
BB5S4	[M]Alveolar adenocarcinoma
Byu20	[X]Mal neop/bronchus
**Chronic Kidney Disease**	
1Z1..	Chronic renal impairment
1Z10.	Chronic kidney disease stage 1
1Z11.	Chronic kidney disease stage 2
1Z12.	Chronic kidney disease stage 3
1Z13.	Chronic kidney disease stage 4
1Z14.	Chronic kidney disease stage 5
1Z15.	Chronic kidney diseas stage 3A
1Z16.	Chronic kidney diseas stage 3B
1Z17.	CKD stage 1 with proteinuria
1Z18.	CKD stage 1 wthout proteinuria
1Z19.	CKD stage 2 with proteinuria
1Z1A.	CKD stage 2 wthout proteinuria
1Z1B.	CKD stage 3 with proteinuria
1Z1C.	CKD stage 3 wthout proteinuria

1Z1D.	CKD stage 3A with proteinuria
1Z1E.	CKD stge 3A wthout proteinuria
1Z1F.	CKD stage 3B with proteinuria
1Z1G.	CKD stge 3B wthout proteinuria
1Z1H.	CKD stage 4 with proteinuria
1Z1J.	CKD stage 4 wthout proteinuria
1Z1K.	CKD stage 5 with proteinuria
1Z1L.	CKD stage 5 wthout proteinuria
661M2	CKD self-managemnt plan agreed
661N2	CKD self-manage plan review
66i..	CKD monitoring
6AA..	Chronic kid dis annual review
7A606	Creation graft fist dialysis
7A619	Ligat arterioven dialysis fist
7A61A	Ligat arterioven dialysis grft
7B00.	Transplantation of kidney
7B000	Autotransplant of kidney
7B001	Live donor kidney transplant
7B002	Cadaver donor kidney transplnt
7B003	Allotra kidney cad
7B004	Allot kid cad
7B005	Allotranspl kidney cadaver NEC
7B006	Xenograft renal transplant
7B00y	Transplantation of kidney OS
7B00z	Transplantation of kidney NOS
7B012	Bilateral nephrectomy
7B063	Exploratn of renal transplant
7B0F1	Pre-trans kid work-up
7B0F3	Post-tran kidney exam
7L1A.	Compensation for renal failure
7L1A0	Renal dialysis
7L1A1	Peritoneal dialysis
7L1A2	Haemodialysis NEC
7L1A4	Automated peritoneal dialysis
7L1A5	Cont ambulat periton dialysis
7L1A6	Peritoneal dialysis NEC
7L1Ay	Compensation for renal fail OS
7L1Az	Compensation renal failure NOS
7L1B.	Place ambu app comp renal fail
7L1B0	Insert ambul perit dial cathet
7L1B1	Remove ambul perit dial cathet
7L1B2	Flush peritoneal dialysis cath
7L1By	Pl amb app comp ren fail OS
7L1Bz	Pl amb app comp ren fail NOS
7L1C.	Place oth app comp renal fail
7L1Cy	Place app comp ren fail OS
7L1Cz	Place app comp ren fail NOS
8L50.	Renal transplant planned
9hE0.	Ex ch kid dis qu ind: Pat uns
9hE1.	Ex ch kid dis qua ind: Inf dis
C104.	Diab.mell. with nephropathy
C1040	Diab.mell.+nephrop - juvenile
C1041	Diab.mell.+nephropathy - adult
C104y	Oth specfd diab mel+renal comp
C104z	Diab.mell.+nephropathy NOS
C1080	Insuln-dep diab mel+renal comp
C1090	Non-ins-dp diab mel+renal comp
C109C	NIDDM with nephropathy

C10A2	Malnut-rlt diab mel+renal comp
C10E0	Type 1 d m with renal comps
C10ED	Type 1 diab mell + nephropathy
C10F0	Type 2 diab mell + renal compl
C10FC	Type 2 diab mell + nephropathy
D215.	Anaemia second renal failure
D2150	Anaemia secondary to CRF
G22..	Hypertensive renal disease
G220.	Malignant hypertens.renal dis.
G221.	Benign hypertensive renal dis.
G222.	Hypertens renal dis+renal fail
G22z.	Hypertensive renal disease NOS
G23..	Hypertensive heart+renal dis.
G230.	Malig.hypert.heart+renal dis.
G231.	Benign hypert.heart+renal dis.
G232.	Hypert ht&ren d+(congs)ht fail
G233.	Hypertn hrt&ren dis+renal fail
G234.	Hyp ht&ren d+both(con)h&r fail
G23z.	Hypertens.heart+renal dis.NOS
G72D.	Aneurysm dialysis AV fistula
G72D0	Anrym sprfls atry dlys AV fstl
G72D1	Anurysm nedl st dlys AV fistul
G72D2	Anurysm anst st dlys AV fistul
Gy10.	Stenosis dialysis AV graft
Gy11.	Stenosis dialysis AV shunt
Gy110	Stenosis art side dia AV shunt
Gy111	Stenosis ven side dial AV sunt
Gy20.	Thromb dialysis AV graft
Gy21.	Thromb dialysis AV fistula
Gy22.	Thromb dialysis AV shunt
Gy3..	Occlusion dialysis vasc access
Gy30.	Occl dialysis AV graft
Gy31.	Occl dialysis AV fistula
Gy32.	Occl dialysis AV shunt
Gy40.	Infect dialysis AV graft
Gy41.	Infect dialysis AV fistula
Gy42.	Infect dialysis AV shunt
Gy50.	Haemorrhage dialysis AV graft
Gy51.	Haemorrhge dialysis AV fistula
Gy52.	Haemorrhage dialysis AV shunt
Gy60.	Rupture dialysis AV graft
Gy61.	Rupture dialysis AV fistula
Gy62.	Rupture dialysis AV shunt
K05..	Chronic renal failure
K050.	End stage renal failure
K051.	Chronic kidney disease stage 1
K052.	Chronic kidney disease stage 2
K053.	Chronic kidney disease stage 3
K054.	Chronic kidney disease stage 4
K055.	Chronic kidney disease stage 5
K080.	Renal osteodystrophy
K080z	Renal osteodystrophy NOS
K08yA	Proteinuric diabetic nephrop
K0D..	End-stage renal disease
K0E..	Acute-on-chronic renal failure
K13C.	Chr drug-induced renal disease
Kyu03	[X]Glomerulr disordrs/diab mel
Kyu21	[X]Other chronic renal failure

SP06B	Contin amb per dial ass perit
SP07G	Sten arterioven dialysis fist
SP083	Kidney transplant fail+rejectn
SP08C	Accel reject renal transplant
SP08E	Acut reject renal trans grad I
SP08F	Acu reject renal trans grad II
SP08G	Acu reject rena trans grad III
SP08H	Acute reject renal transplant
SP08J	Chr rejection
SP08K	Chr rejec
SP08L	Chr rejec
SP08M	Chr rejec
SP08N	Unexplaind renal trans dysfunc
SP08R	Renal transplant rejection
SP08T	Urological complicatn renal Tx
SP08W	Vasc complictn renal transplnt
SP0E.	Disorders of PD
SP0E0	Bloodstained PD effluent
SP0E1	Thrombus in PD catheter
TB001	Kidney transplant+complication
ZV420	[V]Kidney transplanted
ZV451	[V]Renal dialysis status
ZV56.	[V]Aftercare+intermit.dialysis
ZV560	[V]Aftercare+extracorp.dialys.
ZV561	[V]Preparatory care/dialysis
ZV56y	[V]OS aftercare+intermit.dialy
ZV56z	[V]Uns.aftercare+intermit.dial
**Obstructive Sleep Apnoea**	
Fy03.	Sleep apnoea
H5B0.	Obstructive sleep apnoea
**Rhinosinusitis**	
2D33.	O/E - nasal polyp present
74029	Excision polyp nasal septum
74060	Nasal polypectomy
74116	Removal of antrochoanal polyp
7416D	FESS/Post op remov polyps (LA)
7416F	FESS - polypectomy nasal sinus
H11..	Nasal polyps
H110.	Polyp of nasal cavity
H1100	Choanal polyp
H110z	Polyp of nasal cavity NOS
H111.	Polypoid sinus degeneration
H1110	Woakes' ethmoiditis
H111z	Polypoid sinus degenerat.NOS
H11y.	Other polyp of sinus
H11y0	Polyp of frontal sinus
H11y1	Polyp of ethmoidal sinus
H11y2	Polyp of maxillary sinus
H11y3	Polyp of sphenoidal sinus
H11yz	Other polyp of sinus NOS
H11z.	Nasal polyp NOS
H120.	Chronic rhinitis
H1200	Chronic simple rhinitis
H1201	Chronic catarrhal rhinitis
H1202	Chronic hypertrophic rhinitis
H1203	Chronic atrophic rhinitis
H1204	Chronic infective rhinitis
H1205	Chronic ulcerative rhinitis

H1206	Chronic membranous rhinitis
H1207	Chronic fibrinous rhinitis
H120z	Chronic rhinitis NOS
H13..	Chronic sinusitis
H130.	Chronic maxillary sinusitis
H131.	Chronic frontal sinusitis
H132.	Chronic ethmoidal sinusitis
H133.	Chronic sphenoidal sinusitis
H135.	Recurrent sinusitis
H13y.	Other chronic sinusitis
H13y0	Chronic pansinusitis
H13yz	Other chronic sinusitis NOS
H13z.	Chronic sinusitis NOS
H17..	Allergic rhinitis
H170.	Allergic rhinitis - pollens
H171.	Allerg.rhinit.-other allergens
H172.	Allergic rhinitis-unsp allerg
H17z.	Allergic rhinitis NOS
Hyu21	[X]Other allergic rhinitis
Hyu22	[X]Other chronic sinusitis
Hyu23	[X]Other polyp of sinus
**Pulmonary Fibrosis**	
23E5.	O/E - fibrosis of lung present
A114.	Tuberculous fibrosis of lung
H41..	Asbestosis
H41z.	Asbestosis NOS
H423.	Massive silicotic fibrosis
H431.	Bauxite fibrosis of lung
H433.	Graphite fibrosis of lung
H4642	Chemical pulmonary fibrosis
H48..	Progressive massive fibrosis
H4y1.	Chronic pulm.radiation disease
H4y10	Radiation pulmonary fibrosis
H4y1z	Chronic pulm.radiation dis.NOS
H4y21	Chr drg-indc interst lung diso
H55..	Postinflammatory pulm.fibrosis
H563.	Idiopath.fibrosing alveolitis
H5631	Diffuse pulmonary fibrosis
H5632	Pulmonary fibrosis
H5633	Usual interstitial pneumonitis
H563z	Idiopath.fibrosing alveol.NOS
h8M..	PIRFENIDONE
h8M1.	ESBRIET 267mg capsules
h8M2.	PIRFENIDONE 267mg capsules
**Bronchiectasis**	
A115.	Tuberculous bronchiectasis
H34..	Bronchiectasis
H340.	Recurrent bronchiectasis
H341.	Post-infective bronchiectasis
H34z.	Bronchiectasis NOS
P861.	Congenital bronchiectasis
**Diabetes**	
2BBF.	Retina abnormal - diabet relat
2BBk.	O/E- R st treat prol diab ret
2BBl.	O/E- L st treat prol diab ret
2BBL.	O/E - diabet maculop both eyes
2BBo.	O/E - sight threat diab retin
2BBP.	O/E - right eye back diab ret

2BBQ.	O/E - left eye back diab ret
2BBr.	Impair vision due diab retinop
2BBR.	O/E - R eye preprolif diab ret
2BBS.	O/E - L eye preprolif diab ret
2BBT.	O/E - R eye prolif diab ret
2BBV.	O/E - L eye prolif diab ret
2BBW.	O/E - R eye diab maculopathy
2BBX.	O/E - L eye diab maculopathy
2G510	Foot abnormal-diabetes related
2G5A.	O/E-Right diabet foot at risk
2G5B.	O/E-Left diabet foot at risk
2G5C.	Foot abnormal-diabetes related
2G5d.	O/E - L diab foot at incre rsk
2G5e.	O/E - R diab foot at incre rsk
2G5E.	O/E - R diab foot at low risk
2G5F.	O/E - R diab foot at mod risk
2G5G.	O/E - R diab foot at high risk
2G5H.	O/E - R diab foot - ulcerated
2G5I.	O/E - L diab foot at low risk
2G5J.	O/E - L diab foot at mod risk
2G5K.	O/E - L diab foot at high risk
2G5L.	O/E - L diab foot - ulcerated
2G5V.	O/E - R chron diab foot ulcer
2G5W.	O/E - L chron diab foot ulcer
661M4	Diabet self-manage plan agreed
661N4	Diabetes self-manage pln revw
66A2.	Follow-up diabetic assessment
66A3.	Diabetic on diet only
66A4.	Diabetic on oral treatment
66A5.	Diabetic on insulin
66AH.	Diabetic treatment changed
66AH0	Conversion to insulin
66AH1	Convrsn to insulin in 2ry care
66AH2	Convrsn to insul diab spec nrs
66AH3	Conversn non-insulin injet med
66Ai.	Diabetic 6 month review
66AI.	Diabetic - good control
66AJ.	Diabetic - poor control
66AJ1	Brittle diabetes
66AJz	Diabetic - poor control NOS
66AK.	Diabetic - cooperative patient
66AL.	Diabetic-uncooperative patient
66Am.	Insulin dose changed
66An.	Diabetes type 1 review
66AN.	Date diabetic treatment start
66Ao.	Diabetes type 2 review
66Ap.	Insulin treatment initiated
66AP.	Diabetes: practice programme
66AQ.	Diabetes: shared care program.
66AQ1	Decl conse diab year care prog
66AR.	Diabetes management plan given
66As.	Diab on subcutaneous treatment
66AS.	Diabetic annual review
66AS0	Diabetes Yr of Cre annual revw
66At0	Type I diabetic dietary review
66At1	Type II diabetic dietary revie
66Au.	Diab erectile dysfunction rev
66AU.	Diabetes care by hospital only

66Av.	Diabetic ass erect dysfunction
66AV.	Diabetic on insulin+oral treat
66o0.	Incretin mimetic treatmnt strt
679c.	Insulin administratn education
679L0	Educa self management diabetes
679L2	Education diabetes and driving
679R.	Pt offered diab struct ed prog
67D8.	Provisn diab clin summary
7276.	Pan retinal photocoag diabetes
7L100	Contin subcut infusion insulin
7L198	Subcutaneous injection insulin
8A12.	Diabetic crisis monitoring
8A13.	Diabetic stabilisation
8B3l.	Diabetes medication review
8BAi.	Insulin passport completed
8BAj.	Inf dis not carry insulin pass
8BAm.	Insulin passport checked
8BL2.	Pt on max tol ther for diabet
8CE02	Insulin passport given
8CMW7	Diabetes clinical pathway
8CP2.	Transition DM care opt discuss
8CR2.	Diabetes clin management plan
8CS0.	Diabetes care plan agreed
8H2J.	Admit diabetic emergency
8H3O.	Non-urgent diabetic admission
8HBG.	Diab retinopathy 12 mth review
8HKE.	Diabetology D.V. requested
8HLE.	Diabetology D.V. done
8HME.	Listed for Diabetology admissn
8I3k.	Insulin therapy declined
9h42.	Excep diabet qual ind: Inf dis
9kL..	Insulin init - enh serv admin
9m07.	Exc diab ret scr undr ophthalm
9m08.	Exclu diab ret screen as blind
9N1Q.	Seen in diabetic clinic
9NN9.	Under care of diab spec nurse
9OL1.	Attends diabetes monitoring
9OL2.	Refuses diabetes monitoring
9OLA.	Diabetes monitor. check done
9OLD.	Diabet pt unsuit dig ret photo
C10..	Diabetes mellitus
C100.	Diab.mell. - no complication
C1000	Diab.mell.no comp. - juvenile
C1001	Diab.mell.no comp. - adult
C100z	Diab.mell.no comp. - onset NOS
C101.	Diab.mell.with ketoacidosis
C1010	Diab.mell.+ketoacid - juvenile
C1011	Diab.mell.+ketoacid - adult
C101y	Oth specfd diab mel+ketoacidos
C101z	Diab.mell.+ketoacid -onset NOS
C102.	Diab.mell. + hyperosmolar coma
C1020	Diab.mell+hyperosm.coma-juveni
C1021	Diab.mell.+hyperosm.coma-adult
C102z	Diabetes+hyperosmolar coma NOS
C103.	Diab.mell. + ketoacidotic coma
C1030	Diab.mell.+ketoac coma-juvenil
C1031	Diab.mell.+ketoac coma - adult
C103y	Oth specif diab mell with coma

C103z	Diab.mell.+ketoac coma NOS
C104.	Diab.mell. with nephropathy
C1040	Diab.mell.+nephrop - juvenile
C1041	Diab.mell.+nephropathy - adult
C104y	Oth specfd diab mel+renal comp
C104z	Diab.mell.+nephropathy NOS
C105.	Diab.mell.+ eye manifestation
C1050	Diab.mell.+eye manif -juvenile
C1051	Diab.mell.+eye manif - adult
C105y	Oth specfd diab mel+ophth comp
C105z	Diab.mell.+eye manif NOS
C106.	Diab.mell. with neuropathy
C1060	Diab.mell.+neuropathy-juvenile
C1061	Diab.mell.+neuropathy - adult
C106y	Oth specf diab mel+neuro comps
C106z	Diab.mell.+neuropathy NOS
C107.	Diab.mell.+periph.circul.dis
C1070	Diab.+periph.circ.dis-juvenile
C1071	Diab.+periph.circ.dis.-adult
C1072	Diabetic gangrene - adult
C1073	IDDM periph circulatory disord
C1074	NIDDM periph circulat disord
C107y	Oth spcf diab mel+per circ cmp
C107z	Diab.+periph.circ.disease NOS
C108.	Insulin depnd diabetes melitus
C1080	Insuln-dep diab mel+renal comp
C1081	Insul-dep diab mel+ophth comps
C1082	Insul-dep diab mel+neuro comps
C1083	Insul dep diab mel+multi comps
C1084	Unstab insul depend diab mell
C1085	Insul depen diab mel+ulcer
C1086	Insulin depen diab mel+gangren
C1087	Insul-depend diab mell+retinop
C1088	Insul dep diab mell-poor contr
C1089	Insulin dep diabet adult onset
C108A	Insulin-dependent dm no comp
C108B	IDDM with mononeuropathy
C108C	IDDM with polyneuropathy
C108D	IDDM with nephropathy
C108E	IDDM with hypoglycaemic coma
C108F	IDDM with diabetic cataract
C108G	IDDM with peripheral angiopath
C108H	IDDM with arthropathy
C108J	IDDM with neuropath arthropath
C108y	Oth specf diab mel+multip comp
C108z	Unspecifd diab mel+multip comp
C109.	Non-insulin depd diabetes mell
C1090	Non-ins-dp diab mel+renal comp
C1091	Non-ins-dp diab mel+ophth comp
C1092	Non-ins-dp diab mel+neuro comp
C1093	Non-ins-dp diab mel+multi comp
C1094	Non-insul depen diab mel+ulcer
C1095	Non-insulin dep diab mell+gang
C1096	Non-insul dep diab mel+retinop
C1097	Non-insul dep diab-poor contr
C1099	Non-insul-dep diab mel no comp
C109A	NIDDM with mononeuropathy
C109B	NIDDM with polyneuropathy

C109C	NIDDM with nephropathy
C109D	NIDDM with hypoglycaemic coma
C109E	NIDDM with diabetic cataract
C109F	NIDDM with periph angiopath
C109G	NIDDM with arthropathy
C109H	NIDDM with neuropath arthrop
C109J	Insul treated Type 2 diab mell
C109K	Hyperos non-ket stat typ 2 d m
C10A.	Malnutritn-relat diab mellitus
C10A0	Malnutrtn-reltd diab mell+coma
C10A1	Malnut-rlat diab mell+ketoacid
C10A2	Malnut-rlt diab mel+renal comp
C10A3	Malnut-rlt diab mel+ophth comp
C10A4	Malnut-rlt diab mel+neuro comp
C10A5	Malnut-rlt diab mel+per circ c
C10A6	Malnut-rlt diab mel+multi comp
C10A7	Malnut-rlt diab mel w'out comp
C10AW	Maln-rel diab m + unsp comp
C10AX	Maln-rel diab m+ot sp comps
C10B.	Diabet mel induced by steroids
C10B0	Sterod ind diab mel w/out comp
C10C.	Diab mell aut dom
C10D.	Diab mell aut dom type 2
C10E.	Type 1 diabetes mellitus
C10E0	Type 1 d m with renal comps
C10E1	Type 1 diab mell + ophth comps
C10E2	Type 1 diab mell + neuro comps
C10E3	Type 1 diab mell + mult comps
C10E4	Unstab type 1 diabet mellitus
C10E5	Type 1 diab mell with ulcer
C10E6	Type 1 diab mell with gangrene
C10E7	Type 1 diab mell + retinopathy
C10E8	Type 1 diab mell poor control
C10E9	Type 1 diab mell matur onset
C10EA	Type 1 diab mell without comp
C10EB	Type 1 diab mell + mononeurop
C10EC	Type 1 diab mell + polyneurop
C10ED	Type 1 diab mell + nephropathy
C10EE	Type 1 diab mell + hypo coma
C10EF	Type 1 diab mell + diab catar
C10EG	Type 1 diab mell+periph angiop
C10EH	Type 1 diab mell + arthropathy
C10EJ	Type 1 diab mell+neuro arthrop
C10EK	Type 1 d m + persist proteinur
C10EL	Type 1 d m + persist microalb
C10EM	Type 1 d m with ketoacidosis
C10EN	Type 1 d m+ketoacidotic coma
C10EP	Type 1 d m + exudat maculopath
C10EQ	Type 1 dm with gastroparesis
C10ER	Latent autoimm diab mell adult
C10F.	Type 2 diabetes mellitus
C10F0	Type 2 diab mell + renal compl
C10F1	Type 2 diab mell+ophthal comp
C10F2	Type 2 diab mell + neurol comp
C10F3	Type 2 diab mell + multip comp
C10F4	Type 2 diab mell with ulcer
C10F5	Type 2 diab mell + gangrene
C10F6	Type 2 diab mell + retinopathy

C10F7	Type 2 diab mell+poor control
C10F9	Type 2 diab mell without comp
C10FA	Type 2 diab mell mononeurop
C10FB	Type 2 diab mell + polyneurop
C10FC	Type 2 diab mell + nephropathy
C10FD	Type 2 diab mell+hypogly coma
C10FE	Type 2 diab mell+diab catarct
C10FF	Type 2 diab mell+perip angiop
C10FG	Type 2 diab mell + arthropathy
C10FH	Type 2 diab mell neurop+arthr
C10FJ	Insul treated Type 2 diab mell
C10FK	Hyperos non-ket stat typ 2 d m
C10FL	Type 2 d m + persist proteinur
C10FM	Type 2 d m + persist microalb
C10FN	Type 2 d m with ketoacidosis
C10FP	Type 2 d m+ketoacidotic coma
C10FQ	Type 2 d m + exudat maculopath
C10FR	Type 2 dm with gastroparesis
C10FS	Matern inherited diabetes mell
C10G.	Secondary pancreatic dm
C10G0	Second pancr dm without comp
C10H.	DM induced by non-steroid drug
C10H0	DM ind non-ster dru withou com
C10M.	Lipoatrophic diabetes mellitus
C10M0	Lipoatrophic dm without comp
C10N.	Secondary diabetes mellitus
C10N0	Secondary d m without comp
C10N1	Cyst fibro relat diab mellitus
C10y.	Diab.mell.+other manifestation
C10y0	Diab.mell.+oth manif.-juvenile
C10y1	Diab.mell.+other manif. -adult
C10yy	Oth spec diab mel+oth spec cmp
C10yz	Diab.mell.+other manifest NOS
C10z.	Diab.mell. + unspec comp
C10z0	Diab.mell.+comp NOS - juvenile
C10z1	Diab.mell.+comp NOS - adult
C10zy	Oth specf diab mel+unspec comp
C10zz	Diab.mell. + unspec comp NOS
C11y0	Steroid induced diabetes
Cyu2.	[X]Diabetes mellitus
Cyu20	[X]Oth specf diabetes mellitus
Cyu21	[X]Maln-rel diab m+ot sp comps
Cyu22	[X]Maln-rel diab m + unsp comp
Cyu23	[X]Unspec diab mel + ren compl
F1711	Autonomic neuropathy-diabetes
F3450	Diabet mononeuritis multiplex
F35z0	Diabetic mononeuritis NOS
F372.	Polyneuropathy in diabetes
F3720	Acute painful diab neuropathy
F3721	Chron painful diab neuropathy
F3722	Asymptomatic diab neuropathy
F3813	Myasthenic syndrome+diabetes
F3y0.	Diabetic mononeuropathy
F420.	Diabetic retinopathy
F4200	Background diabetic retinopath
F4201	Proliferative diabetic retinop
F4202	Preproliferative diabetic ret
F4203	Advanced diabetic maculopathy

F4204	Diabetic maculopathy
F4205	Advanced diabetic retinal dis
F4206	Non prolif diab retinop
F4207	High risk prolif diab retinop
F4208	High risk non prolif dia retin
F420z	Diabetic retinopathy NOS
F4213	Hypertensive retinopathy
F4407	Diabetic iritis
G73y0	Diabetic peripheral angiopathy
K01x1	Nephrotic syndrome+diabetes M.
K08yA	Proteinuric diabetic nephrop
Kyu03	[X]Glomerulr disordrs/diab mel
L1805	Pre-ex diab mell/insuln-depend
L1806	Pre-ex diab mel non insuln-dep
L1807	Pre-ex malnutrtn-rlat diab mel
L180X	Pre-existing diab mel
M0372	Cellulitis in diabetic foot
M21yC	Insulin lipohypertrophy
M2710	Ischaemic ulcer diabetic foot
M2711	Neuropathic diab ulcer - foot
M2712	Mixed diabetic ulcer - foot
N0300	Diabetic cheiroarthropathy
N0301	Diabetic Charcot arthropathy
PKyP.	Diab ins
R0542	[D]Gangrene of toe in diabetic
R0543	[D]Widespread diab foot gangr
ZV6DA	[V]Admitted commence insulin
ZV6DB	[V]Admitted conversion insulin

References

1 Benson T. The history of the Read codes: the inaugural James Read Memorial Lecture 2011. *Journal of Innovation in Health Informatics* **19**, 173-182, doi:10.14236/jhi.v19i3.811 (2011).

2 NHS Digital Technology Reference Data Update Distribution (TRUD). *READ Codes*, <https://isd.digital.nhs.uk/trud3/user/guest/group/0/pack/9> (2019).

3 NHS Digital. *Quality Outcomes Framework (QOF)*, <https://digital.nhs.uk/data-and-information/data-tools-and-services/data-services/general-practice-data-hub/quality-outcomes-framework-qof> (2019).

4 NHS Digital. *Quality and Outcomes Framework (QOF), enhanced services and core contract extraction specifications (business rules)*, <https://digital.nhs.uk/data-and-information/data-collections-and-data-sets/data-collections/quality-and-outcomes-framework-qof> (2019).

5 NHS Digital. *Secondary Uses Service (SUS)*, <http://content.digital.nhs.uk/sus> (2018).

6 World Health Organisation. *Classifications*, <http://www.who.int/classifications/icd/en/> (2018).

7 NHS Digital. *NHS Data Model and Dictionary Version 3: Admission Method*, <https://www.datadictionary.nhs.uk/data_dictionary/attributes/a/add/admission_method_de.asp> (2019).

8 NHS Digital. *NHS Data Model and Dictionary Version 3: Accident and Emergency Diagnosis Tables*, <http://www.datadictionary.nhs.uk/web_site_content/supporting_information/clinical_coding/accident_and_emergency_diagnosis_tables.asp> (2018).

9 Quanjer PH *et al.* Lung volumes and forced ventilatory flows. Official statement of the European Respiratory Society. *Eur Respir J* **6**, 5-40 (1993).

10 Jones RC *et al.* Derivation and validation of a composite index of severity in chronic obstructive pulmonary disease: the DOSE Index. *Am J Respir Crit Care Med* **180**, 1189-1195, doi:10.1164/rccm.200902-0271OC (2009).

11 Sundh J, Montgomery S, Stallberg B & Lisspers K. Assessment of COPD in primary care: new evidence supports use of the DOSE index. *Prim Care Respir J* **22**, 142-143, doi:10.4104/pcrj.2013.00050 (2013).
